# Harnessing large-scale genomics-based screening for the identification of expansin genes responsive to abiotic stress in *Peucedanum praeruptorum*

**DOI:** 10.3389/fpls.2026.1983847

**Published:** 2026-10-15

**Authors:** Canguang He, Modan Qi, Xueru Li, Jun Dai, Cheng Song

**Affiliations:** 1 School of Pharmacy, Anhui University of Chinese Medicine, Hefei, China; 2 Anhui Dabie Mountain Institute of Traditional Chinese Medicine, West Anhui University, Lu’an, China; 3 School of Life and Health, West Anhui University, Lu’an, China

**Keywords:** abiotic stress, expansin, expression patterns, Peucedanum praeruptorum, phylogenetic analysis

## Abstract

**Introduction:**

Expansins are crucial for cell wall relaxation and significantly impact plant growth and development. However, the expansin gene family in Peucedanum praeruptorum remains to be systematically characterized.

**Methods:**

Here, we systematically identified expansin genes in P. praeruptorum and analysed their molecular characteristics, evolutionary patterns, and expression profiles in response to methyl jasmonate (MeJA). Subcellular localization prediction, gene structure analysis, chromosomal localization, collinearity analysis, and cis-acting element analysis were performed. Based on cis-element screening, PpdEXPA01, PpdEXPA10, PpdEXPA13, PpdEXPB1, and PpdEXPB2 were selected to validate their expression under exogenous MeJA stress.

**Results:**

A total of 39 PpdEXP genes were identified and classified into four subfamilies: EXPA (24 genes), EXPB (9 genes), EXPLA (2 genes), and EXPLB (4 genes). Subcellular localization predictions indicated that all members are localized in the cell wall, and all expansin proteins are hydrophilic. Gene structural analysis showed that the number of introns ranged from one to four and that all genes contained two conserved domains: DPBB_EXPA_N and Expansin_C. Chromosomal localization analysis showed that the 39 PpdEXP genes were distributed across 11 chromosomes, with tandem duplication events occurring on chromosomes 3, 4, and 7. Collinearity analysis indicated that segmental and whole-genome duplication were the main driving forces behind the expansion of the expansin gene family. Analysis of cis-acting elements revealed 1,237 elements in total, which were primarily classified into three categories: hormone response, light response, and stress response. Expression profiling revealed that PpdEXP genes exhibit significant tissue specificity. The five selected genes had significant tissue- and time-specific expression patterns in roots, stems, and leaves under exogenous MeJA stress.

**Discussion:**

This study systematically elucidated the molecular basis and expression regulation patterns of the P. praeruptorum expansin family, providing a foundation for further functional characterization of PpdEXP genes and their potential roles in growth, development, and stress responses.

## 1 Introduction

Expansins are a category of primordial cell wall-loosening proteins prevalent in terrestrial plants and their algal progenitors (Chase et al., 2020). In the absence of hydrolase activity, plant expansins promote the passive stretching of the cell wall cellulose network under tensile stress. This unlocks the polysaccharide network, facilitating cell wall relaxation and expansion (Samalova et al., 2022). Plant expansins are small, two-domain proteins typically composed of around 270 amino acid residues. Most possess a cleavage signal peptide, including approximately 25 amino acid residues at the N-terminus, which facilitates secretion to the cell wall (Chen et al., 2024). Their standard configuration is torpedo-shaped, consisting of two functionally discrete domains: Domain I and Domain II (Zhang et al., 2025). Domain I, identified as DPBB_EXPA_N (129 amino acids), constitutes the N-terminal domain of the expansin protein. It encases a double Psi-β barrel (DPBB) core and features a conserved His-Phe-Asp (HFD) motif, which constitutes the fundamental structural basis for cell wall softening. This region exhibits similarity with the catalytic domain of glycosidase family 45 (GH45) proteins; however, it does not possess their catalytic function. Its primary function is to facilitate cell wall relaxation and non-enzymatic remodeling, thereby contributing to plant development and stress responses. Domain II is Expansin_C (75 amino acids), the C-terminal domain of expansins, analogous to Group 2 grass pollen allergens and exhibiting sequence characteristics of timothy grass allergens. It collaborates with Domain I to engage in non-enzymatic cell wall remodeling. This domain was recently designated as a family 63 carbohydrate-binding module (CBM63) (Monschein et al., 2023).

The plant expansin protein superfamily consists of four subfamilies: α-expansins (EXPA), β-expansins (EXPB), expansin-like A (EXPLA), and expansin-like B (EXPLB) (Han et al., 2019). The EXPA and EXPB subfamilies are the most extensive and have undergone significant research, whereas the EXPLA and EXPLB subfamilies are more preserved and have attracted less focus on their functionality. *Arabidopsis thaliana* possesses 26 *EXPA* genes and 6 *EXPB* genes, whereas *Oryza sativa* has 34 *EXPA* genes and 21 *EXPB* genes (Dabravolski and Isayenkov, 2025). *EXPA* predominantly targets cellulose-cellulose connections, facilitating relaxation in acidic environments, a vital characteristic for cell wall expansion prompted by auxin and other growth factors. EXPB predominantly targets xylan, contributing to cell wall remodeling and control (Cosgrove, 2024). Expansins play a significant role in numerous biological processes, such as cell elongation, organ development, fruit softening, and responses to abiotic stress (Sampedro and Cosgrove, 2005; Marowa et al., 2016; Jin et al., 2020). Their roles have been confirmed in tomato (Liu et al., 2024), potato (Cosgrove, 2015; Liu et al., 2025), soybean (Xing et al., 2025), rice (Zhang et al., 2024a), wheat (Mira et al., 2023), sweet potato (Zhang et al., 2024b), and tobacco (Marowa et al., 2020). For example, overexpression of the sweet potato *IbEXP1* gene in *Arabidopsis* significantly increased growth rate and seed yield, whereas overexpression of *NtEXPA11* in tobacco regulated plant growth and development while enhancing tolerance to abiotic stress (Marowa et al., 2020; Bae et al., 2014).

The traditional Chinese medicine ‘Qianhu’ is derived from the dried roots of *P. praeruptorum*, a species within the genus *Peucedanum* of the Apiaceae family. It possesses medicinal properties, including the regulation of qi, resolution of phlegm, dispelling of wind, and clearance of heat. It predominantly comprises coumarin-type active chemicals (Wang et al., 2024; Song et al., 2026). *P. praeruptorum* is harvested from winter to early spring, either when the stems and leaves have withered or before the flowering stems emerge (Song et al., 2021, 2024). It is predominantly located in the provinces of Shandong, Shaanxi, Anhui, Jiangsu, Zhejiang, Fujian, Guangxi, Jiangxi, Hunan, Hubei, and Sichuan (Liu et al., 2026). The yield and quality of the medicinal material are directly determined by the elongation and swelling of the *P. praeruptorum* root system, and cell wall expansion is a core process in root morphogenesis. As key cell wall-relaxing factors, proteins of the expansin family are extensively involved in the morphogenesis and development of plant vegetative organs (roots, stems and leaves). Their functional specialization is closely related to their tissue-specific expression. Members predominantly expressed in roots may contribute to root extension and swelling, whereas those preferentially expressed in stems and leaves may be linked to tissue expansion, aligning with the established function of expansins in cell wall loosening and growth (Cosgrove, 2024). Although prior research has established the pivotal regulatory function of expansin family genes in organ development among other plant species, comprehensive investigations on the tissue-specific expression patterns of these genes in the medicinal plant *P. praeruptorum* are absent. Given that the root is the principal medicinal organ of *P. praeruptorum*, expansin family genes are hypothesized to facilitate root elongation, radial swelling, stem elongation, and leaf expansion by regulating cell enlargement, thereby ultimately impacting medicinal yield and quality (Liu et al., 2026). This study systematically identified members of the expansin gene family and analysed their evolutionary conservation using bioinformatics methods, including phylogenetic analysis, conserved domains, gene structure, chromosomal mapping, and cis-acting elements in the promoter region, based on the *P. praeruptorum* genome data. The study focused on analyzing the tissue-specific expression patterns of expansin genes in roots, stems, and leaves, screening for candidate genes with organ-specific expression preferences, and preliminarily validating their expression regulatory characteristics through promoter hormone response element prediction and exogenous hormone treatment. The results provide candidate genes and theoretical support for regulating the vegetative organ development in *P. praeruptorum*, elucidating stress response mechanisms, and improving the quality of its medicinal parts.

## 2 Material and methods

### 2.1 Data sources and identification of *P. praeruptorum* expansin genes

This study utilised genomic data of *P. praeruptorum*, sequenced independently by our research group, as the core research material. Reference genomes were downloaded from the NCBI Genome Database (Accession No. PRJNA910498) and the FigShare database (https://figshare.com/articles/dataset/Peucedanum_praeruptorum_genome_assembly_and_gene_annotations/21743984/1). The sequence names of *Arabidopsis* Expansin proteins were retrieved from the TAIR database (https://www.arabidopsis.org/browse/gene_family/expansin), and their corresponding amino acid sequences were fully extracted via the TAIR batch sequence download platform (https://v2.arabidopsis.org/tools/bulk/sequences/index.jsp). To screen for family members of the *P. praeruptorum* expansin gene, the study employed a dual-screening strategy combining local BLAST searches with the Hidden Markov Model (HMMER): First, whole-genome BLASTp alignments were performed using known *Arabidopsis* Expansin protein sequences, with an e-value set at 10^-5^. Simultaneously, the *P. praeruptorum* genome was systematically scanned using Hidden Markov Model seed files (PF03330, PF01357, PF22514) for characteristic Expansin domains (Li et al., 2022). Candidate genes were validated using both the NCBI Conserved Domain Database (CDD; https://www.ncbi.nlm.nih.gov/cdd) and the InterPro functional annotation platform (https://www.ebi.ac.uk/interpro/). Strictly adhering to the definition criteria for the Expansin family, short fragments, truncated, and redundant sequences were removed, retaining only candidate genes that simultaneously contained the double-stranded β-barrel domain (DPBB_1, cd00204) and the Expansin-specific domain (EXPN, cl23733) (Blum et al., 2021, 2025; Lv et al., 2020).

### 2.2 Analysis of physical and chemical properties

This work employed various bioinformatics analytic platforms to ascertain the physicochemical properties and subcellular localization of the target proteins. Specifically, the online analytical system created by the Department of Health Technology at the Technical University of Denmark (DTU Health Tech, https://services.healthtech.dtu.dk/), we systematically characterized the full-length sequence features of the target proteins and their transmembrane domain parameters, including the number of transmembrane domains, their topological structure, and the corresponding amino acid spans. To analyse the physicochemical properties of the proteins, we utilised the ProtParam tool from the ExPASy bioinformatics resource portal to conduct exact calculations and extract essential values, including the theoretical isoelectric point (pI) and molecular weight (kDa) (Gasteiger et al., 2005). For subcellular localization prediction, this study utilised the Cell-PLoc 2.0 package. This tool integrates amino acid composition features with sequence motif information to enable multi-level prediction of the subcellular localization of proteins (Raza et al., 2022).

### 2.3 Construction of the phylogenetic tree of *P. praeruptorum* expansin proteins

In this study, the MEGA11 software was used to perform phylogenetic analysis on 63 Expansin protein sequences, including those from *Arabidopsis thaliana*, *Oryza sativa*, and *P. praeruptorum* (Arslan et al., 2021; Xi et al., 2025). The Neighbor-Joining (NJ) approach was used to create a phylogenetic tree following multiple sequence alignment using the ClustalW program. The p-distance method was selected as the genetic distance model. During the analysis, pairwise deletion was performed on ambiguous sites for each pair of sequences, and branch node confidence was assessed through 1,000 bootstrap repetitions; the support values for the clustering of relevant taxonomic units were directly annotated on the corresponding branches (Tamura et al., 2021).

### 2.4 Gene structure of *P. praeruptorum* expansin gene family

We conducted a visual study of the gene structures of *P. praeruptorum* members of the expansin gene family using the Gene Structure View module in the TBtools software (v. 2.2.3) (Chen et al., 2020). By aligning genomic sequences with their corresponding CDS (coding sequences), this module accurately annotates the distribution patterns of exons and introns, thereby systematically elucidating the structural composition of expansin genes.

### 2.5 Examination of the conserved motifs and domains within *P. praeruptorum* expansin proteins

In this study, we used the MEME Suite (https://meme-suite.org/meme/tools/meme) to predict conserved motifs in members of the expansin gene family from *P. praeruptorum* (Guo et al., 2023). This study systematically discovered conserved domains in the protein sequences of 39 members of the expansin gene family using the NCBI Batch Conserved Domain (CD) Search platform. The defining functional domains of Expansin proteins—DPBB_EXPA_N (N-terminal double Psi-β barrel domain) and Expansin_C (C-terminal cellulose-binding domain)—were identified and preserved as essential functional modules. The Visualise NCBI CDD Domain Pattern module of the TBtools software was utilised to depict the distribution patterns of DPBB_EXPA_N and Expansin_C domains, producing high-resolution topological maps that distinctly represent their linear organization and relative placements among various members.

### 2.6 Chromosomal mapping of *P. praeruptorum* expansin genes

The chromosomal coordinates of genes are precisely retrieved and visualised by the “Gene Location Visualise from GTF/GFF” module in TBtools by analysing genomic annotation files in GFF/GTF format (Chen et al., 2020).

### 2.7 Collinearity analysis of *P. praeruptorum* expansin genes

MCScanX was used to examine syntenic relationships among homologous genomic regions in *P. praeruptorum*, and a genome-wide collinearity map was generated to characterize duplication patterns and evolutionary relationships within the expansin gene family.

### 2.8 Examination of cis-acting regions inside the promoters of the expansin gene family

Based on the genomic sequence data of *P. praeruptorum*, candidate promoter regions were specifically extracted from genomic sequences within 2000 bp upstream of the start codon (ATG) of expansin gene family members. Additionally, we methodically identified cis-regulatory regions inside the specified promoter sequences utilising an online analytic platform based on the PlantCARE database to clarify the putative transcriptional regulation network of the expansin gene family (Lescot et al., 2002).

### 2.9 Expression characteristics of expansin gene family

HISAT2 software was used to align high-quality paired-end transcriptomic sequencing data—which had been filtered for quality control using Trimmomatic—the reference genome sequence published by Kim et al (Kim et al., 2019). Subsequently, transcript quantification was performed using the Stringtie tool to calculate the FPKM expression values for each gene across all samples. Heatmaps of tissue-specific expression were generated by hierarchical clustering analysis to systematically examine the expression patterns of the Expansin gene family in various tissues of *P. praeruptorum*. In this study, the pre-bolting and post-bolting stages were defined according to the sampling time points corresponding to the plant growth period, rather than morphological criteria. The pre-bolting samples were collected at the time point when plants were still in the vegetative growth stage and had not yet bolted, whereas the post-bolting samples were collected at the time point when plants had completed bolting and entered the reproductive growth stage. Root, stem, and leaf tissues were harvested synchronously at each time point to ensure comparability among samples.

### 2.10 Plant tissue culture and hormone treatment

The seeds were acquired from Zhongxi Town in Ningguo City, Anhui Province, and subsequently cultivated at the botanical garden at West Anhui University. Healthy seedlings that had grown consistently were selected and transplanted into 10 cm × 8 cm × 8 cm pots containing 300 g of soil and three seedlings each. Following a three-week acclimatisation period under natural light conditions, the seedlings underwent exogenous hormone administration. Following the identification of cis-acting elements, MeJA was chosen as an exogenous hormone to examine its impact on gene expression across several tissues of *P. praeruptorum*. The seedlings were treated with a 100 μmol/L MeJA solution by uniform spraying until the leaves were completely wet (Mao et al., 2023). Samples of root, stem, and leaf tissues were obtained at 1.5, 3, 9, and 12 hours following treatment. Control, blank, and MeJA-treated groups were established with three biological replicates for each time point and tissue type. After collection, the samples were immediately frozen in liquid nitrogen and stored at -80 °C for later use.

### 2.11 RNA extraction and real-time quantitative PCR analysis

Total RNA was extracted from each tissue sample using an RNA extraction kit (TIANGEN, Beijing, China) according to the manufacturer’s instructions. First-strand cDNA was synthesized using the FastSensi One-Step Genomic cDNA Synthesis Premix (TIANGEN, Beijing, China) with 500 ng of total RNA as a template. Real-time quantitative PCR was performed on a LightCycler^®^ 96 real-time quantitative PCR instrument (Roche, Germany). The reaction mixture (20 μL) was prepared according to the LightCycler^®^ recommended protocol: First, 3.0 μL of PCR-grade water, 2.0 μL of 10× PCR primer and 10.0 μL of 2× Master Mix (TIANGEN, Beijing, China) were added to a 1.5 mL reaction tube in sequence, and the mixture was mixed gently by pipette pipetting. Then, dispense 15 μL of the mixture into each well of a 96-well plate, followed by the addition of 5 μL of cDNA template to each well. The plate was sealed with a LightCycler^®^ 480 sealing film, placed in a centrifuge equipped with a basket rotor, balanced, and centrifuged at 1,500 × g for 2 min before being loaded into the instrument for reaction. Load the plate into the LightCycler^®^ 96 instrument for reaction. The reaction program is as follows: First, pre-denature at 95 °C for 10 min; then perform a three-step amplification reaction for 40 cycles, consisting of 10 s denaturation at 95 °C, 10 s reaction at 60 °C (annealing temperature), and 10 s extension at 72 °C. Following amplification, perform melting curve analysis: denature at 95 °C for 10 s, anneal at 65 °C for 60 s, then slowly heat from 65 °C to 97 °C at a rate of 0.2 °C/s. Finally, automatically cool to 40 °C and hold for 30 s. The *SAND* gene was used as a reference gene (forward primer: ACAGAAGAGCCTCATGAATCCC; reverse primer: CAAGCAAAGGCGTCATATCAAA) (**Table 1**). Each sample was tested in triplicate for both technical and biological replicates (Zhao et al., 2016). Data acquisition and analysis were performed using LightCycler^®^ 96 SW 1.1 software, and relative expression levels were calculated using the 2^^-^ΔΔCt^ method (Li et al., 2024a).

**Table 1:** Specific primer sequences used for qRT-PCR analysis.

Primer name	Primer sequence (5’–3’)
*SAND*-F	ACAGAAGAGCCTCATGAATCCC
*SAND*-R	CAAGCAAAGGCGTCATATCAAA
*PpdEXPA01*-F	TATTTGCTCAGCTTTTGGGCA
*PpdEXPA01*-R	GTTGCATGAGCATTGAGCCA
*PpdEXPA10*-F	AGTAGTTTGTGCGAGGAGACT
*PpdEXPA10*-R	CGCTTTACACATGGGACCCT
*PpdEXPA13*-F	TATTGGTCCGAGTCCCCTGT
*PpdEXPA13*-R	GGTTGTGGAATGAGGGGTGT
*PpdEXPB1*-F	TGTGTTTATGTGTCCGTGTGC
*PpdEXPB1*-R	TCCCTGGTCAACGTGGAATG
*PpdEXPB2*-F	TCTGGCAAGGAATGTGGAGC
*PpdEXPB2*-R	CCTGTCCCTTAATGAACACAAGC

## 3 Results

### 3.1 Identification, physicochemical characteristics, and prediction of subcellular localization of the PpdEXP genes

This study employed systematic screening utilising Hidden Markov Model (HMMER) and BLASTP methodologies to uncover 39 members of the expansin gene family within the *P. praeruptorum* genome. By aligning these genes with the identified expansin genes from *Arabidopsis* and rice, they were classified into four subfamilies: 24 EXPA (*PpdEXPA01*–*PpdEXPA24*), 9 EXPB (*PpdEXPB1*–*PpdEXPB9*), 2 EXPLA (*PpdEXPLA1*–*PpdEXPLA2*) and 4 EXPLB (*PpdEXPLB1*–*PpdEXPLB4*). The physicochemical properties of the proteins exhibited considerable diversity in the lengths of the amino acid sequences across PpdEXP family members (ranging from 146 to 387 amino acids), with an average length of 258 amino acids. The molecular weight (MW) varied from 15.85 to 43.26 kDa, with a mean molecular weight of 27.96 kDa. Isoelectric point (pI) study reveals that the proteins in this family display notable amphiphilic properties, with pI values spanning from 4.74 to 9.92. Among them, 7 proteins (17.9%) have a pI < 7 and exhibit acidic protein characteristics, while 32 proteins (82.1%) have a pI > 7 and exhibit basic protein characteristics. Subcellular localization predictions show that all PpdEXP proteins are localized to the cell wall. This outcome is strongly aligned with the traditional functional role of expansin proteins as cell wall relaxants, hence reinforcing the credibility of the identification results in this investigation (Men et al., 2026). A physicochemical investigation of the PpdEXP protein family was performed utilising the Protscale bioinformatics tool on the ExPASy online analysis platform. The results showed that all members of this family exhibit hydrophilic characteristics. According to the hydrophilicity analysis results and the principles of protein solubility, it can be deduced that PpdEXP proteins likely demonstrate stable solubility under physiological settings (**Table 2**).

**Table 2:** Basic information on PpdEXPs.

Gene name	Gene ID	Number of amino acids	MW	PI	Subcellular localization prediction
*PpdEXPA03*	BHQH00019681	262	28841.01	9.44	Cell wall
*PpdEXPA04*	BHQH00027553	257	28114.69	9.39	Cell wall
*PpdEXPA02*	BHQH00034476	264	28990.9	8.94	Cell wall
*PpdEXPA15*	BHQH00039178	260	28077.79	9.62	Cell wall
*PpdEXPA16*	BHQH00018453	262	28349.19	9.85	Cell wall
*PpdEXPA23*	BHQH00028191	280	30058.64	9.4	Cell wall
*PpdEXPA21*	BHQH00002570	246	26305.55	8.45	Cell wall
*PpdEXPA14*	BHQH00009467	292	32009.67	9.52	Cell wall
*PpdEXPA24*	BHQH00011998	249	26436.62	8.92	Cell wall
*PpdEXPA19*	BHQH00032937	249	26896.33	9.59	Cell wall
*PpdEXPA20*	BHQH00008677	250	26981.38	9.59	Cell wall
*PpdEXPA09*	BHQH00005568	256	27355.26	9.32	Cell wall
*PpdEXPA07*	BHQH00008492	287	31576.56	9.27	Cell wall
*PpdEXPA10*	BHQH00037746	254	27277.42	9.4	Cell wall
*PpdEXPA22*	BHQH00009931	243	25892.22	8.82	Cell wall
*PpdEXPA17*	BHQH00020504	266	28855.84	9.1	Cell wall
*PpdEXPA18*	BHQH00041736	244	26116.44	5.86	Cell wall
*PpdEXPA12*	BHQH00020772	210	23062.18	9.28	Cell wall
*PpdEXPA13*	BHQH00038741	387	43258.38	9.17	Cell wall
*PpdEXPA01*	BHQH00035146	250	27976.58	9.92	Cell wall
*PpdEXPA11*	BHQH00024901	226	24862.68	8.11	Cell wall
*PpdEXPA05*	BHQH00021440	250	27693.44	8.66	Cell wall
*PpdEXPA06*	BHQH00037422	265	28811.44	8.56	Cell wall
*PpdEXPB8*	BHQH00004129	261	27826.79	8.76	Cell wall
*PpdEXPB9*	BHQH00037469	264	28331.25	8.92	Cell wall
*PpdEXPA08*	BHQH00039587	146	15850.92	9.37	Cell wall
*PpdEXPB7*	BHQH00016472	264	28274.64	5.7	Cell wall
*PpdEXPB3*	BHQH00006132	260	27474.19	6.03	Cell wall
*PpdEXPB1*	BHQH00026384	292	31221.14	7.16	Cell wall
*PpdEXPB5*	BHQH00006137	234	24807.84	7.05	Cell wall
*PpdEXPB6*	BHQH00006136	258	27429.35	7.73	Cell wall
*PpdEXPB2*	BHQH00028630	293	31526.5	8.3	Cell wall
*PpdEXPB4*	BHQH00006133	251	26594.7	5.41	Cell wall
*PpdEXPLB3*	BHQH00019317	256	27415.11	4.74	Cell wall
*PpdEXPLB1*	BHQH00044221	249	27579.33	6.97	Cell wall
*PpdEXPLB4*	BHQH00032252	255	27907.43	5.04	Cell wall
*PpdEXPLA1*	BHQH00041144	262	28211.19	9.05	Cell wall
*PpdEXPLB2*	BHQH00019318	256	27787.66	7.48	Cell wall
*PpdEXPLA2*	BHQH00041143	263	28422.23	8.44	Cell wall

### 3.2 Collinearity analysis and chromosomal localization of PpdEXP genes

Analysis of chromosomal localization utilising GFF files indicated that the 39 PpdEXP genes are extensively dispersed over 11 chromosomes. Tandem duplication events occurred in specific regions of chromosomes 3, 4 and 7, resulting in multiple PpdEXP genes clustering densely in localized regions of these chromosomes (**Figure 1**). The MCScanX software was utilised to conduct an intra-species synteny analysis of the expansin protein family, clarifying the evolutionary relationships of the PpdEXP gene family. In the visual map, red connecting lines specifically indicate gene duplication events associated with segmental and whole-genome duplication (**Figure 2**).

**Figure 1:**
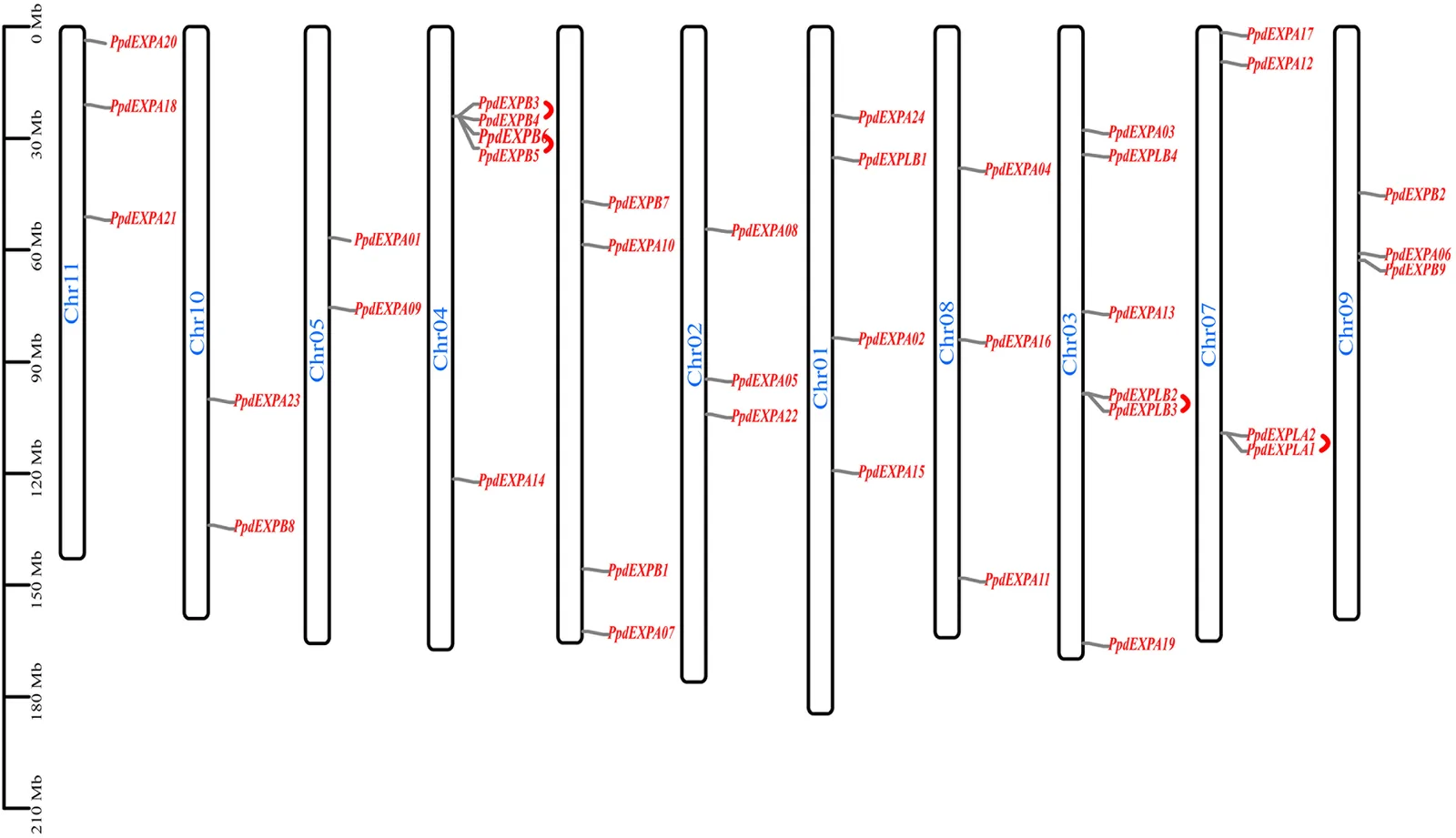
Chromosomal distribution of PpdEXP genes. Red connecting lines indicate tandem duplication events.

**Figure 2:**
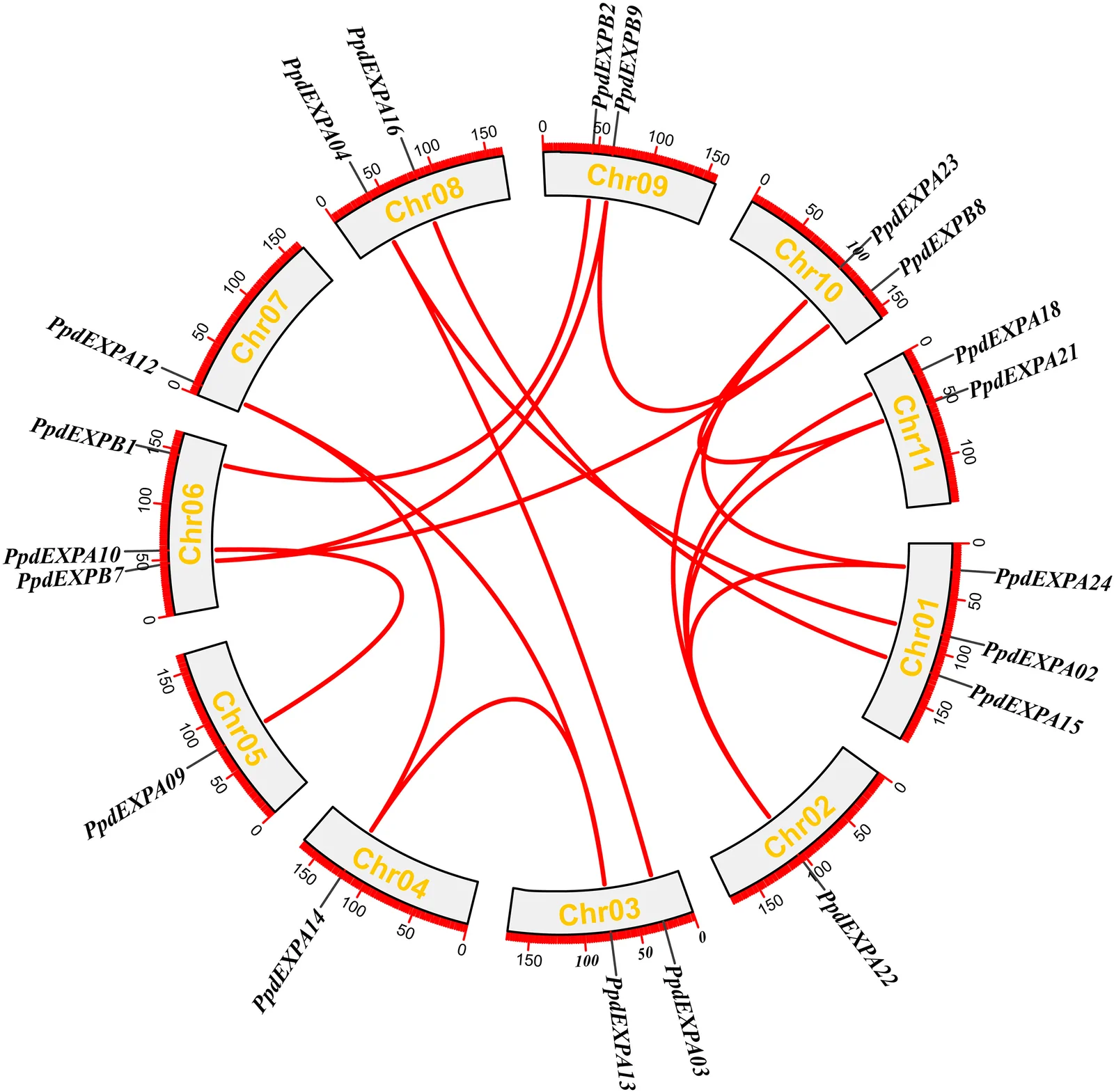
Analysis of synteny concerning PpdEXP genes in *P. praeruptorum*. Red connecting lines denote whole-genome duplication and segmental duplication events.

### 3.3 Phylogenetic analysis of the PpdEXP gene family

Phylogenetic research indicated that the 39 PpdEXP proteins may be categorised into four subfamilies: EXPA, EXPB, EXPLA, and EXPLB, designated as PpdEXPA, PpdEXPB, PpdEXPLA, and PpdEXPLB, respectively (**Figure 3**). The membership count in each subfamily exhibits considerable variation: the PpdEXPA subfamily comprises 24 members, whereas the PpdEXPLA subfamily contains merely two. This pattern of composition is highly consistent with the distribution characteristics of expansin protein families in *Arabidopsis* and rice (Cosgrove, 2024). The PpdEXPA subfamily is found on the same evolutionary branch as the AtEXPA and OsEXPA subfamilies. These genes primarily disrupt non-covalent bonds between polysaccharides, acting at the cellulose-to-cellulose interface and mediating pH-dependent cell wall creep via a non-enzymatic mechanism. They participate in processes like cell elongation, organ growth, fruit softening, and reactions to abiotic stress. It may be deduced that PpdEXPA members predominantly govern biological activities including cell multiplication in *P. praeruptorum*, the development of vegetative organs, and the capacity to adapt to unfavourable environments (Zhang et al., 2025). The PpdEXPB subfamily clusters into a distinct branch with AtEXPB and OsEXPB, suggesting that genes in this subfamily may primarily target cell wall matrix polysaccharides (Li et al., 2003). In grasses, EXPB proteins specifically act on arabinogalactan and are involved in processes such as pollen tube penetration, root elongation, and stress responses (Cosgrove, 2015). As a dicotyledon, members of the PpdEXPB subfamily in *P. praeruptorum* may retain functions that are conserved across species, such as the regulation of meristematic growth, pollen development and cell wall plasticity (Feng et al., 2022). The PpdEXPLA and PpdEXPLB subfamilies are distinct and highly conserved evolutionary clades that demonstrate close phylogenetic relationships with the EXPLA and EXPLB genes found in species such as *Arabidopsis* and rice. EXPLA and EXPLB belong to subfamilies that emerged late in the evolution of seed plants. They are chiefly engaged in the development of root architecture, the modulation of nutrient absorption, seed germination, and responses to abiotic stressors, including drought and salinity. As they lack direct cell wall creep activity, it is inferred that PpdEXPLA and PpdEXPLB members mainly regulate processes such as root development, seed germination and tolerance to abiotic stresses. The phylogenetic divergence and differences in member numbers observed within the same subfamily among PpdEXP, AtEXP, and OsEXP indicate that the expansin gene family exhibits distinct species-specific evolutionary characteristics in *P. praeruptorum*, *A. thaliana*, and *O. sativa*. This also demonstrates that the various subfamilies possess both conservation and divergence at both functional and evolutionary levels (Lee et al., 2001).

**Figure 3:**
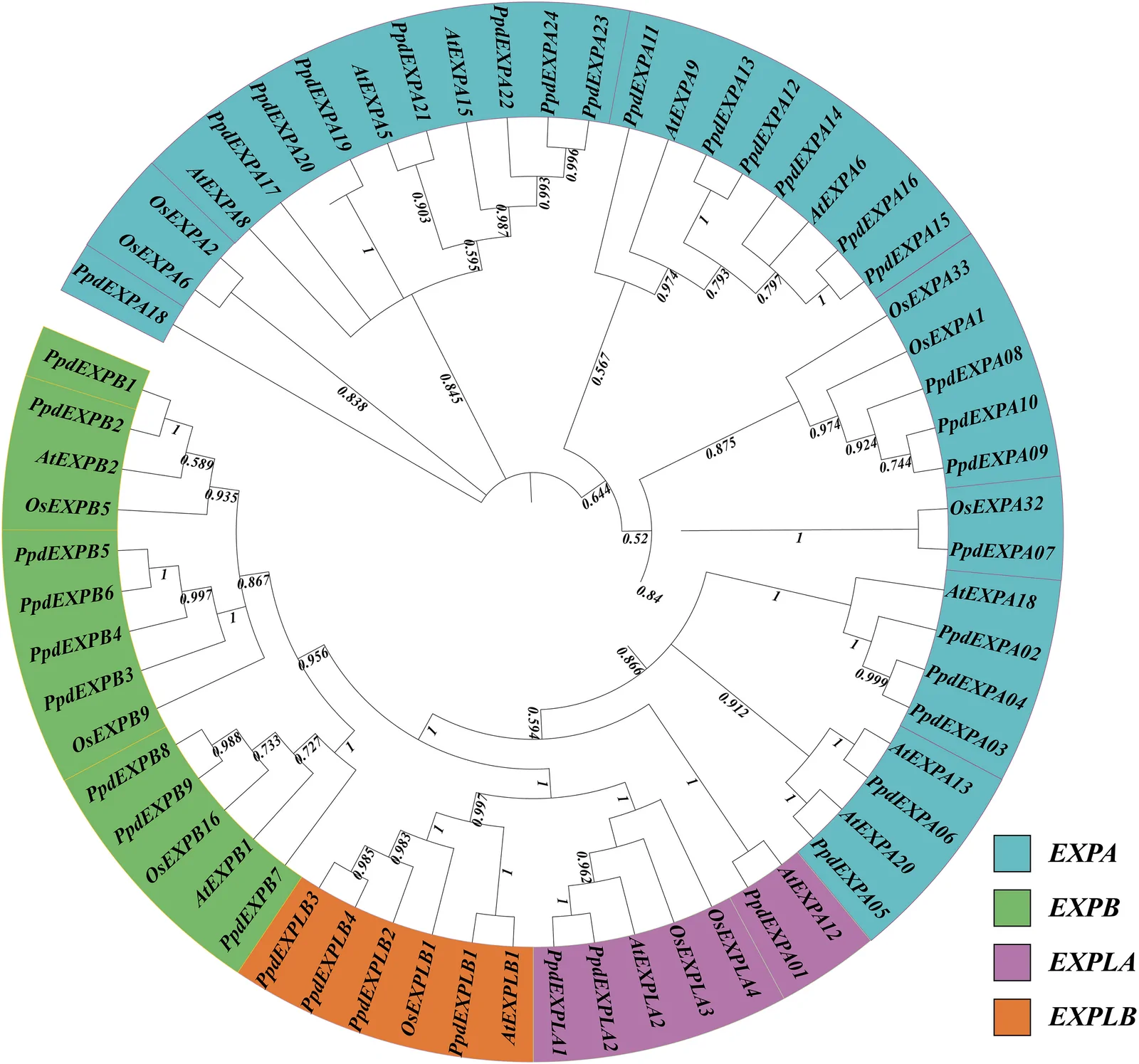
Phylogenetic tree of PpdEXP proteins among *A. thaliana*, *O. sativa* and *P. praeruptorum*.

### 3.4 Examination of the gene architecture of the PpdEXP gene family

Structural diversity is essential in the evolution of various gene families. Analysis of exon and intron structures typically reveals variations in gene composition, whereas the 5’ and 3’ untranslated regions (UTRs), crucial for controlling gene transcription, mRNA stability, and translation efficiency, are significant elements of gene structure. The 39 PpdEXPs exhibit significant variation in UTR distribution and the number of introns and exons, with all 39 containing 1–4 introns (**Figure 4**). Among them, *PpdEXPA13*, *PpdEXPB5*, *PpdEXPLA1*, *PpdEXPLA2*, *PpdEXPLB1*, *PpdEXPLB2*, *PpdEXPLB3*, and *PpdEXPLB4* each contain four introns, while *PpdEXPA05* and *PpdEXPA08* each contain one. The remaining genes contain 2 or 3 introns. Thirteen genes (*PpdEXPA02*, *PpdEXPA03*, *PpdEXPA06*, *PpdEXPA07*, *PpdEXPA10*, *PpdEXPA11*, *PpdEXPA12*, *PpdEXPA14*, *PpdEXPA15*, *PpdEXPA16*, *PpdEXPA17*, *PpdEXPA20*, *PpdEXPA21*) contain 2 introns, while the rest contain 3 introns. Regarding UTR structure, there is significant heterogeneity in the distribution of 5’ and 3’ UTRs among the 39 PpdEXPs. Most genes possess both complete 5’ and 3’ UTRs, such as *PpdEXPA06* and *PpdEXPA09*, while a few genes lack a 5’ UTR and possess only a 3’ UTR, such as *PpdEXPA23*, *PpdEXPA05*. These structural differences reflect the functional differentiation of different expansin genes at the post-transcriptional level and may be closely related to regulatory patterns such as tissue-specific gene expression and responses to environmental stress.

**Figure 4:**
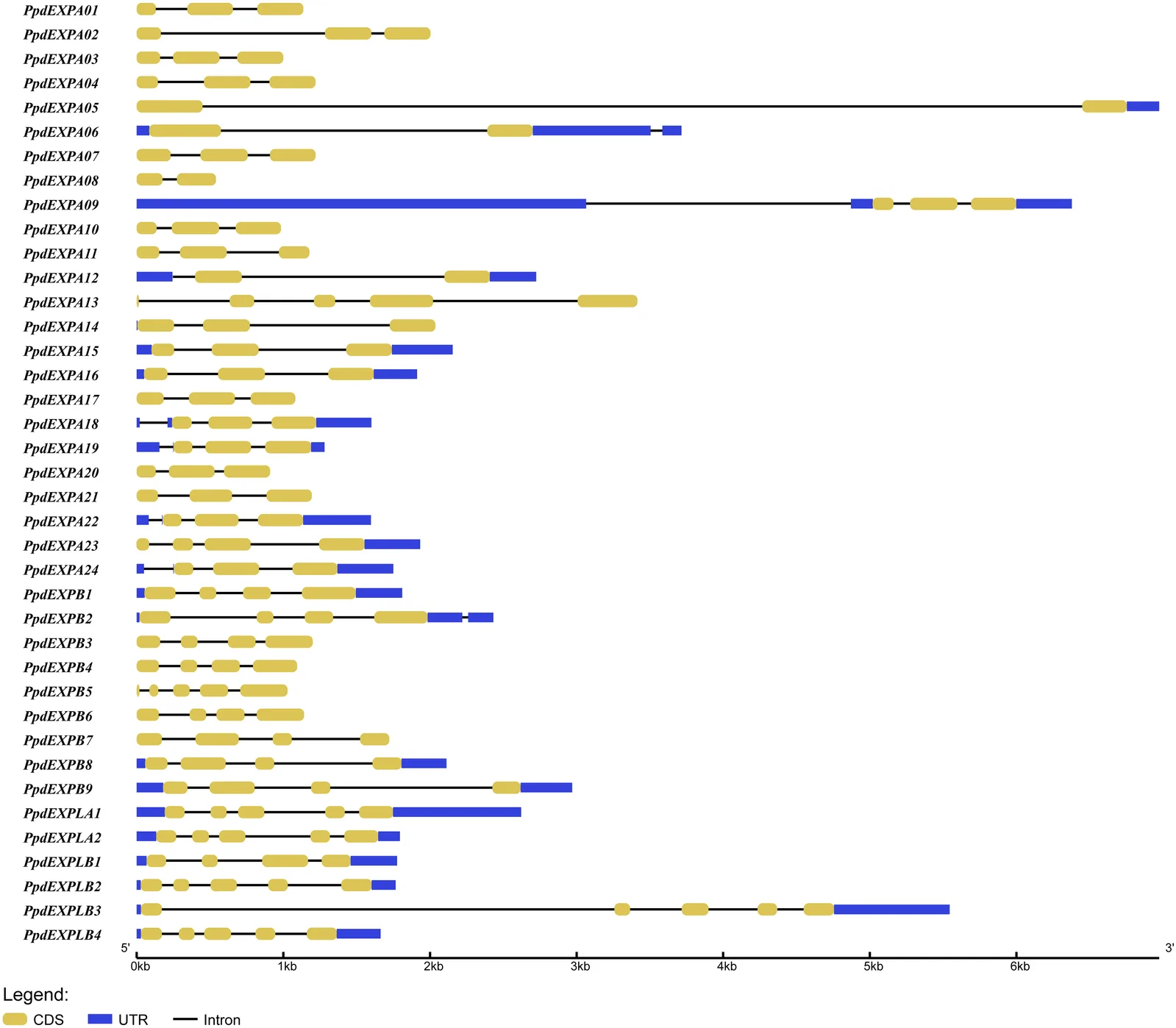
Structural features of PpdEXP genes in *P. praeruptorum*.

### 3.5 Examination of conserved motifs and domains within the PpdEXP Gene Family

To further assess the sequence properties of *P. praeruptorum* PpdEXPs, we examined their conserved motif composition. The findings indicated that 15 unique motifs were discovered from genomes of *Arabidopsis* and rice. PpdEXPs within the same subclade generally exhibit analogous motif compositions, hence reinforcing our subclade classification (**Figure 5A**). Motifs 3, 4 and 6 are present in the majority of PpdEXP proteins, indicating their ubiquity. In contrast, motifs 12, 13 and 15 only appear in certain specific PpdEXP proteins, suggesting that they are specific to these proteins. While almost all PpdEXP members within the same subfamily contain multiple motifs, there are compositional differences between subfamilies. For instance, all PpdEXPA subfamilies contain motifs 3, 2, 4, 9 and 6. All PpdEXPB subfamilies contain motifs 3, 8, 10 and 4, while all PpdEXPLA and PpdEXPLB subfamilies contain motifs 3, 11, 15, 8, 10, 4 and 6. The DPBB_EXPA_N domain and the Expansin_C domain are present in every gene. These domains are hallmarks of this gene family (**Figure 5B**).

**Figure 5:**
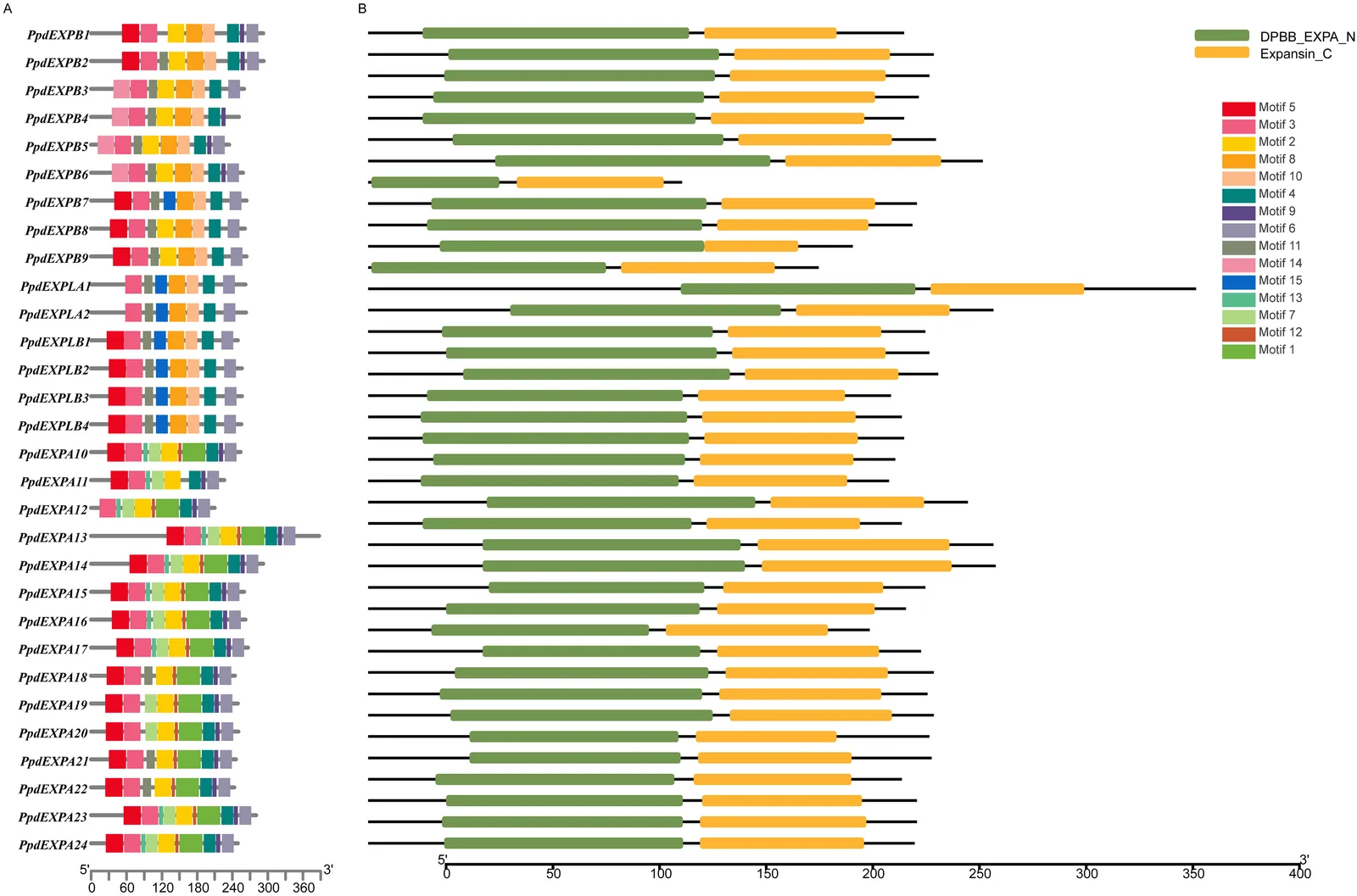
*P. praeruptorum*’s conserved PpdEXP protein motifs and domains. **(A)** Protein sequences with conserved motifs; **(B)** PpdEXP proteins with major domains.

### 3.6 Examination of cis-acting elements in PpdEXP Genes

Transcription factors situated in gene promoter regions are regulatory sequences that are essential for gene expression and the modulation of numerous biological processes (Schmitz et al., 2022). To investigate the regulatory mechanisms by which PpdEXP genes govern plant development and stress responses, we utilized the PlantCARE database to discover transcription factors located within the 2,000 bp area upstream of the ATG start codon of each PpdEXP gene. A total of 1,237 cis-regulatory elements were found within the promoter regions of PpdEXP genes (**Supplementary Table S1**). These elements are linked to 27 biological functions. *PpdEXPA13* had the greatest quantity of cis-elements (61), with notable diversity among subfamilies. All cis-elements identified in this investigation can be categorised into three groups (**Figure 6**). Category I involves hormone responses (395), including ABRE response elements (85; 6.9%), ARE response elements (69; 5.6%), CGTCA-motif (53; 4.3%), TGACG-motif (53; 4.3%), ERE response elements (52; 4.2%), TCA-element response elements (19; 1.5%), TGA-element response elements (19; 1.5%), ABRE3a response elements (16; 1.3%), ABRE4 response elements (16; 1.3%), and TCA response elements (13; 1.1%). The promoter regions of all PpdEXP gene subfamilies are abundant in ABA response elements (ABRE and ARE), methyl jasmonate response elements (CGTCA-motif and TGACG-motif), ethylene response elements (ERE), and auxin response elements (TGA-element). In comparison to the other three subfamilies, members of the PpdEXPA subfamily possess a greater number of ABA response elements, ERE response elements, MeJA response elements, and auxin response elements, whereas members of the PpdEXPLA subfamily exhibit the least of these four hormone-related elements. Among the four hormone response elements, the ARE response element is the most prevalent, identified in 33 PpdEXP genes, followed by the ABRE response element, present in 31 PpdEXP genes. We found that some gene members share multiple hormone response elements, suggesting that they may exhibit a faster and more intense response to certain hormones. Genes such as *PpdEXPA13*, *PpdEXPA14*, *PpdEXPA02*, *PpdEXPA20*, *PpdEXPB1*, *PpdEXPB2* and *PpdEXPLA2* exhibit a higher number of these elements. For example, *PpdEXPLA2* contains many CGTCA- and TGACG-motif elements, whereas *PpdEXPB2* contains many ARE elements. This suggests that they may have a greater capacity to respond to hormone regulation processes. Several PpdEXP genes, including *PpdEXPA01*, *PpdEXPA02*, *PpdEXPA07*, *PpdEXPA12*, *PpdEXPA13*, *PpdEXPB1*, *PpdEXPB2*, *PpdEXPB8*, *PpdEXPLA2*, *PpdEXPLB3* and *PpdEXPLB4*, share multiple hormonal response elements. This suggests that these genes may produce faster and more intense responses to certain hormones. The second category is associated with light response (551), including Myb (172, 13.9%). Notably, MYB-related cis-elements are multifunctional; besides light-signal regulation, they also participate in plant stress-response pathways. Box 4 (141, 11.4%), G-box (84, 6.8%), GT1-motif (43, 3.5%), TCT-motif (39, 3.2%), AE-box (22, 1.8%), GATA-motif (20, 1.6%), MYB recognition site (16, 1.3%), and I-box (14, 1.1%). The PpdEXP gene contains the highest number of Myb-associated cis-elements (172), followed by Box 4-associated elements (141). In contrast to the other three subfamilies, the EXPLA subfamily lacks several of the nine elements, whereas the EXPA subfamily possesses the highest quantity of light-response-related elements, particularly those linked to Myb, Box 4, and G-box expression. The results indicate that members of the EXPA subfamily may serve as significant regulatory elements in plant light response mechanisms. The third category is related to stress responses (291 elements), including stress response elements (125, 10.1%), drought response elements (73, 5.9%), wound response elements (59, 4.7%), cold response elements (34, 2.7%), and specific wound response elements (1, 0.13%). Like the aforementioned categories of response elements, the EXPA subfamily also encompasses the greatest quantity of cis-elements linked to stress response, drought response, wound response, and cold response elements. MBS response elements and TC-rich repeat response elements are lacking in the EXPLA and EXPLB subfamilies. Members of the EXPA subfamily possess the greatest quantity of stress response components, indicating their potential role as principal regulatory factors in stress responses (Cosgrove, 2024). Furthermore, the greatest quantity of stress response elements was identified in PpdEXP genes, signifying that these genes are pivotal in stress responses. The quantity and structure of cis-regulatory elements in the promoter sequences of several PpdEXP genes demonstrate considerable variability both intra- and inter-subfamilially. The results indicate that the expression levels of PpdEXP genes in *P. praeruptorum* are synergistically modulated by various cis-regulatory elements, primarily associated with the regulation of light, hormone, and stress-related biological processes. The PpdEXPA subfamily contained the highest number of hormone-, light-, and stress-responsive cis-elements, whereas the PpdEXPLA subfamily had the fewest. This distribution was consistent with the qRT-PCR results, in which *PpdEXPA01*, *PpdEXPA10*, and *PpdEXPA13* showed the strongest MeJA-induced expression in roots and stems. These results suggest that the enrichment of hormone- and stress-related cis-elements in the PpdEXPA subfamily may underlie its stronger transcriptional response to MeJA signaling.

**Figure 6:**
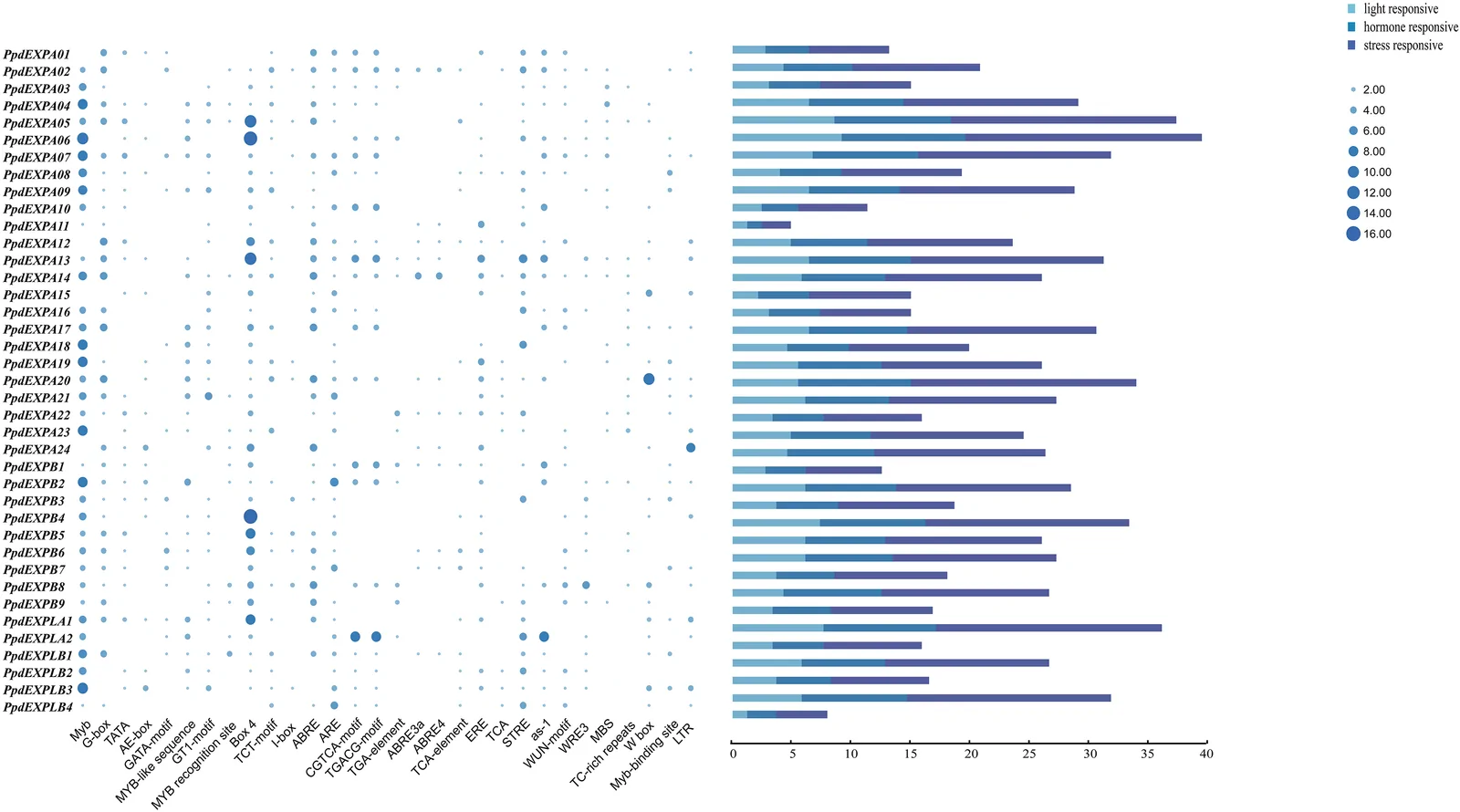
Analysis of cis-acting elements in the promoter regions of PpdEXP genes. Some MYB-type cis-elements exert dual functions in both light and stress responses.

### 3.7 Gene expression analysis of expansin genes involved in different tissues

We conducted a heatmap analysis to examine the expression patterns of the expansin gene family in *P. praeruptorum* across several tissues, as illustrated in **Figure 7**. *PpdEXPA10*, *PpdEXPA04*, and *PpdEXPB1* demonstrated elevated expression levels across many tissues, notably in post-bolting root and stem tissues, where their expression markedly increased, indicating that these genes may be crucial in these contexts (Dong et al., 2022). *PpdEXPA03* and *PpdEXPA20* demonstrated elevated expression levels in pre-booting leaves and stems, likely associated with the distinct activities of these tissues. By contrast, *PpdEXPA02*, *PpdEXPA06* and *PpdEXPA23* showed relatively uniform expression, with no significant up- or downregulation in any specific tissue. This suggests that these genes may have broad functions that are not strongly influenced by tissue specificity.

**Figure 7:**
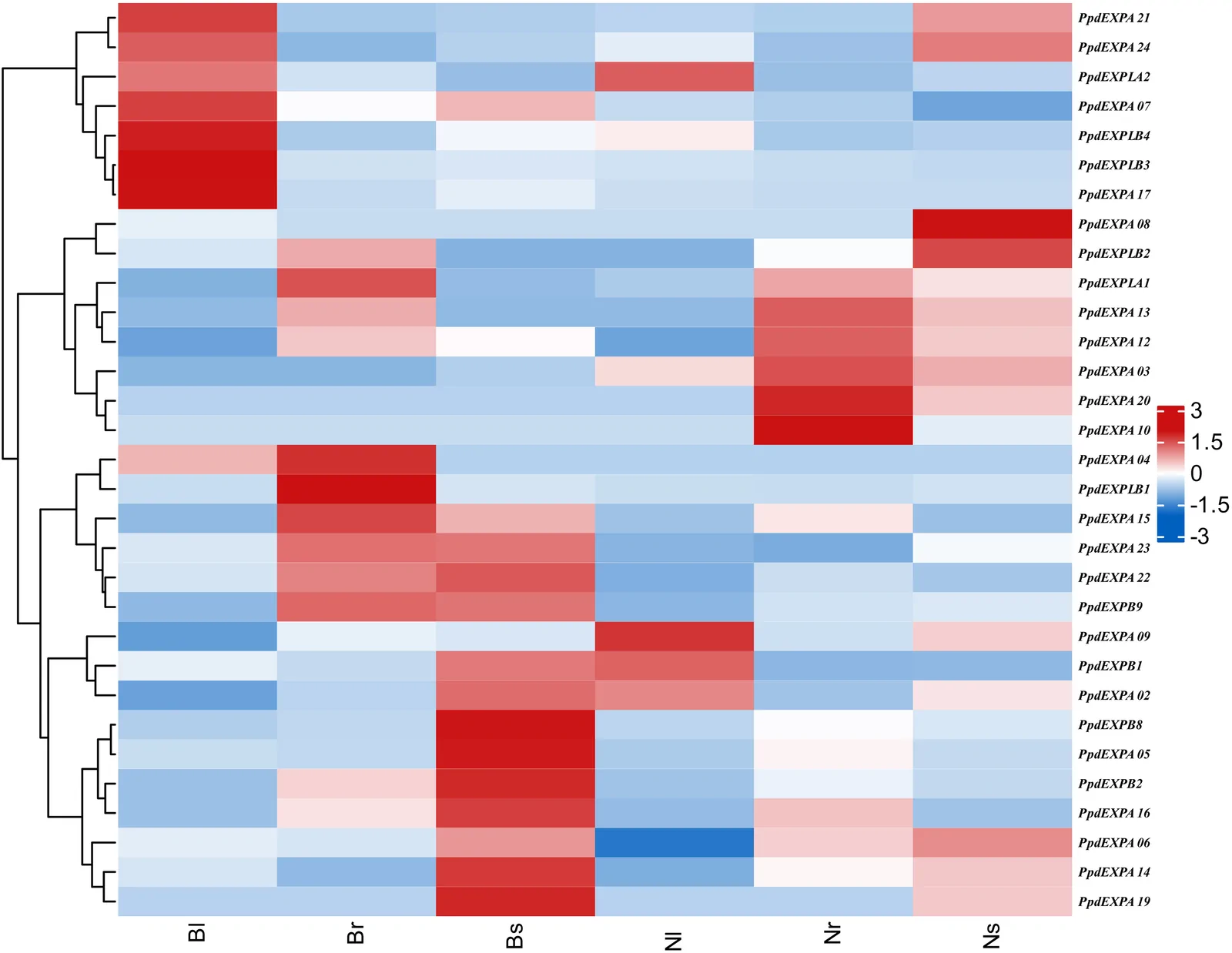
Expression profiles of PpdEXP family members across various tissues. Br, Bs, and Bl denote the root, stem, and leaf subsequent to bolting, respectively. Nr, Ns, and Nl denote the root, stem, and leaf before to bolting, respectively.

### 3.8 Gene expression analysis of PpdEXP genes under hormone treatment

To examine the regulatory influence of exogenous MeJA on the expression of five potential expansin genes in *P. praeruptorum* (*PpdEXPA01*, *PpdEXPA10*, *PpdEXPA13*, *PpdEXPB1*, and *PpdEXPB2*), qRT-PCR analysis was performed to assess alterations in their transcriptional levels in roots, stems, and leaves. Results are expressed as the mean ± SE of three biological replicates; distinct lowercase letters denote significant differences between groups (p < 0.05). At 0 h, the baseline expression of all genes was consistent across all tissues, with no significant differences. Based on the expression profiles following MeJA treatment, the five genes can be classified into three categories according to induction intensity, time-response phase, and tissue-response preference. Based on induction intensity, they can be classified as: highly induced genes (*PpdEXPA01*, *PpdEXPA10*, *PpdEXPA13*), which exhibited significant and strong upregulation in both roots and stems with a high response amplitude; and weakly responsive/early-repressed genes (*PpdEXPB2*), which were significantly repressed in the early stages of treatment but gradually recovered and were induced in the middle and late stages, with a relatively weak overall response intensity. Based on the time of response, genes can be classified as: early-response genes (*PpdEXPA01*, *PpdEXPA10*), which exhibit significant changes in expression as early as 1.5 h; and mid-to-late-response genes (*PpdEXPA13*, *PpdEXPB1*), which reach peak expression at 3–9 h. Based on tissue-specific response patterns, they can be classified as: genes showing strong responses in roots and stems but weak or suppressed expression in leaves (*PpdEXPA01*, *PpdEXPB1*), and genes exhibiting consistent expression trends across all tissues (*PpdEXPB2*).

*PpdEXPA01* demonstrated a notable pattern of substantial induction during the intermediate phase, succeeded by a decrease and suppression in the later phase in roots; conversely, in stems, it exhibited initial suppression, pronounced induction in the intermediate phase, and recovery in the later phase, with the peak response in stems significantly exceeding that in roots. In leaves, it exhibited an induction-followed-by-inhibition pattern, with significant upregulation at 1.5–3 h and marked downregulation at 9–12 h; *PpdEXPA10* showed significant early induction and significant late inhibition in both roots and leaves; in stems, it reached a peak of strong induction at 3 h, followed by a marked downregulation. *PpdEXPA13* showed significant induction in the middle phase and a decline to inhibition in the late phase in both roots and stems, with response intensities in roots and stems far higher than in leaves. In leaves, however, it showed significant induction in the early phase and gradual recovery in the late phase; *PpdEXPB1* showed early induction followed by significant inhibition in roots, intense induction at 3 h in stems, and overall sustained inhibition in leaves. *PpdEXPB2* exhibited highly consistent trends across roots, stems, and leaves: significant inhibition at 1.5 h, sustained induction reaching a peak between 3 and 9 h, followed by a notable drop in roots at 12 hours, but stems and leaves essentially returned to control levels.

Based on a comprehensive evaluation of three criteria—induction intensity, response speed, and tissue specificity—*PpdEXPA01*, *PpdEXPA10*, and *PpdEXPA13*, which exhibited high induction levels and a strong preference for roots and stems, as well as *PpdEXPB1*, which showed strong induction with stem-specificity, were collectively identified as the core MeJA-responsive expansin genes in *P. praeruptorum*. Overall, the response patterns of these five candidate genes to MeJA in the roots, stems, and leaves of *P. praeruptorum* demonstrated notable tissue specificity and temporal dependence. Most genes showed stronger induction in roots and stems, whereas in leaves, they tended to be repressed or exhibited a pattern of initial repression followed by upregulation, suggesting that these genes may have unique and significant functions in the tissue-specific modulation of MeJA signalling in *P. praeruptorum* (**Figure 8**).

**Figure 8:**
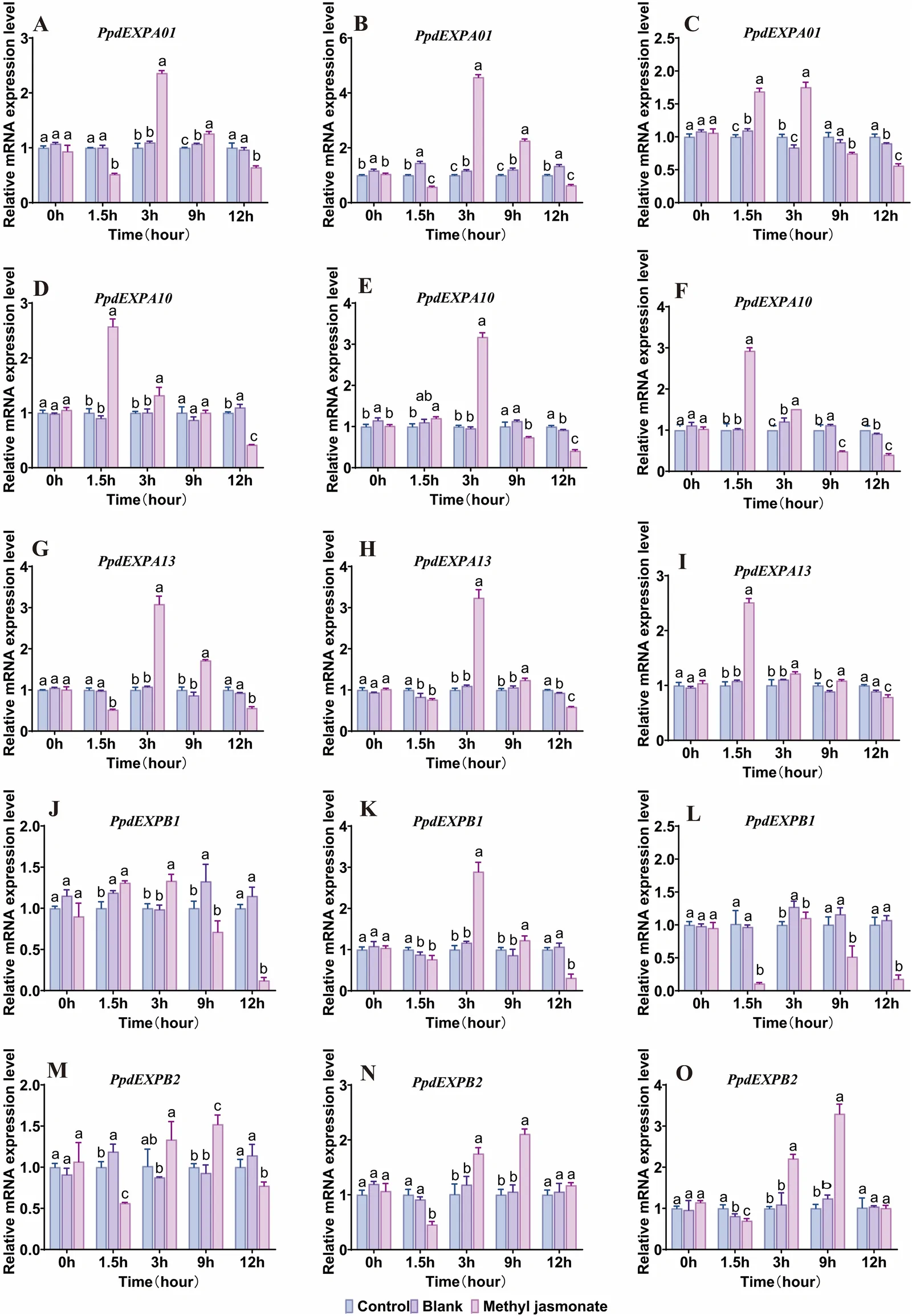
Quantification of certain PpdEXP genes with qRT-PCR. **(A, D, G, J, M)** denote the root. **((B, E, H, K, N)** denote the stem. **(C, F, I, L, O)** denote the leaf. Lowercase letters "a", "b" and "c" represent the results of Tukey's multiple comparison test after one-way ANOVA. Within each subgraph, bars with different letters are significantly different (P < 0.05), while bars with identical letters indicate no significant difference (Comparisons are only valid within each individual subplot and cannot be applied across different subgraphs).

## 4 Discussion

As an important medicinal plant in the Apiaceae family, *P. praeruptorum* contains active coumarin compounds in its dried roots, demonstrating clear medicinal value; the morphogenesis of its vegetative organs directly influences the yield and quality of the medicinal parts. Expansins, as non-enzymatic proteins that facilitate cell wall loosening, promote cell wall relaxation and cellular expansion. They are deeply engaged in plant growth and development, hormonal signal transduction, and stress responses. Although systematic characterisation of expansins has been accomplished in species like rice, sweet potato, and *Salvia miltiorrhiza*, pertinent research in the medicinal Apiaceae plant remains unclarified (Yin et al., 2023). This study, utilising the whole-genome data of *P. praeruptorum* subsp*. sibiricum*, found 39 members of the expansin gene family for the first time, categorised into four subfamilies: PpdEXPA (24), PpdEXPB (9), PpdEXPLA (2), and PpdEXPLB (4). The distribution of subfamily sizes aligns with that observed in species like *Arabidopsis*, sweet potato, and cotton, with the EXPA subfamily being the most extensive and the EXPLA subfamily containing the fewest members, indicating the conservation of this gene family in the evolution of terrestrial plants (Lv et al., 2020). Compared to crops such as sweet potato (59 members), the number of PpdEXP genes in *P. praeruptorum* is relatively low, but similar to that in *Arabidopsis* (36 members) and potato (36 members). This difference may be related to species-specific growth and development strategies and environmental adaptation requirements. The examination of the physicochemical characteristics of the PpdEXP protein family indicated that the amino acid lengths vary from 146 to 387 residues, molecular weights range from 15.85 to 43.26 kDa, and isoelectric points span from 4.74 to 9.92. 82.1% of these proteins are basic, and all members are localised to the cell wall, aligning with the traditional functional localization of Expansins; concurrently, all members have hydrophilic characteristics, establishing a structural foundation for their participation in cell wall relaxing. The examination of gene structure and conserved characteristics reveals that the 39 PpdEXP genes possess 1 to 4 introns and all encompass the two fundamental conserved domains, DPBB_EXPA_N and Expansin_C. Members of the same subfamily exhibit analogous motif compositions; this structural conservation substantiates the dependability of the subfamily classification, although nuanced variations may be essential in driving functional diversity.

Chromosomal mapping analysis revealed that the 39 PpdEXP genes are widely distributed across 11 chromosomes, with tandem duplication events occurring on chromosomes 4, 3, and 7. Collinearity analysis confirmed that segmental duplication and whole-genome duplication are major drivers of the expansion of the PpdEXP gene family. This resembles the evolutionary patterns seen in crops like sweet potato, indicating that gene duplication events offer a biological foundation for the functional diversification of PpdEXP genes (Yu et al., 2024; Li et al., 2024b). Notably, both tandem-duplicated and segmental-duplicated PpdEXP genes belong to the same subfamily, consistent with the duplication patterns observed in sweet potato, suggesting that gene expansion within specific subfamilies may facilitate its adaptation to specific growth environments or developmental requirements. Phylogenetic analysis reveals that PpdEXP genes are grouped with expansin genes from *Arabidopsis* and rice based on subfamilies, with homologous genes within the same subfamily closely clustered across species, indicating that the diversification of the expansin gene family occurred prior to the separation of monocotyledons and dicotyledons. Members of the EXPA and EXPB subfamilies constitute 84.6% of the total PpdEXP genes, aligning with the characteristic that these subfamilies function as the principal functional subfamilies in plants like soybean. This indicates their pivotal involvement in plant growth and development, and it is hypothesised that members of the PpdEXPA and PpdEXPB subfamilies in *P. praeruptorum* may participate in the morphogenesis of the medicinal component (Xiao et al., 2023).

An examination of promoter cis-regulatory elements identified 1,237 elements inside the promoter region of the PpdEXP gene, categorised into three primary types: hormone response, light response, and stress response. Among these, the predominant hormone response elements were abscisic acid response elements (ABRE), methyl jasmonate response elements (CGTCA-motif/TGACG-motif), and ethylene response elements (ERE); members of the PpdEXPA subfamily had a markedly greater quantity of hormone response elements compared to other subfamilies. The PpdEXPA subfamily exhibited enrichment in stress response elements and light response elements, suggesting that the expression of PpdEXP genes is collaboratively regulated by several circumstances, and that distinct subfamilies demonstrate regulatory inclinations. Importantly, the subfamily-specific distribution of cis-acting elements was mirrored by the gene expression responses. The PpdEXPA subfamily, which contained a greater number of ABA-, MeJA-, ethylene-, and auxin-responsive elements than the other subfamilies, also exhibited the strongest induction of representative members (*PpdEXPA01*, *PpdEXPA10*, and *PpdEXPA13*) under MeJA treatment. In contrast, the PpdEXPLA subfamily, with the fewest of these four hormone-related elements, showed relatively weak or limited responses. This correlation suggests that differences in the density and composition of promoter cis-elements may contribute to the differential transcriptional responsiveness among expansin subfamilies, and further indicates that PpdEXPA members are likely to play a central role in MeJA-mediated regulation of vegetative organ development and stress adaptation in *P. praeruptorum.* Analysis of tissue expression profile heatmaps revealed that the expression of the PpdEXP gene family exhibits diversity and tissue specificity, with significant differences in gene expression among different vegetative organs before and after booting. *PpdEXPA10*, *PpdEXPA04*, and *PpdEXPB1* demonstrate elevated basal expression levels and are markedly increased in roots and stems post-booting, indicating their potential involvement in the regulation of secondary growth and development in these tissues following booting; *PpdEXPA03* and *PpdEXPA20* are highly expressed in pre-booting leaves and stems, implying their potential role in cell expansion and organ elongation during the vegetative growth stage; Genes such as *PpdEXPA06*, *PpdEXPA14*, and *PpdEXPA19* exhibit constitutive expression, providing fundamental regulation for normal plant growth and development. Furthermore, hierarchical clustering revealed that some genes share similar expression patterns, potentially forming regulatory modules that collaboratively control tissue growth and development, while the differential expression of genes lays the foundation for their functional specialization.

Analysis of expression patterns under exogenous MeJA treatment revealed that five key PpdEXP genes (*PpdEXPA01*, *PpdEXPA10*, *PpdEXPA13*, *PpdEXPB1*, *PpdEXPB2*) exhibited distinct tissue specificity and time-dependent expression in roots, stems, and leaves. Furthermore, the response patterns in root and stem tissues correlated with the expression characteristics of these tissues in the tissue expression profiles following bolting: *PpdEXPA10* and *PpdEXPB1* exhibited significant induced expression patterns in the roots and stem tissues under MeJA treatment, consistent with their naturally high expression levels in roots and stems after bolting. The expression of these genes is hypothesised to be regulated by the developmental stage of the vegetative organs post-bolting in *P. praeruptorum* and by the activation of the methyl jasmonate hormone signalling pathway; collectively, these elements influence their functional roles in root and stem growth and development. Most other genes demonstrated significant induction in roots and stems but suppression in leaves, or an initial induction followed by suppression, under MeJA treatment. *PpdEXPB2* showed highly consistent response patterns across all three tissues: significant suppression at 1.5 h, followed by sustained induction reaching a peak between 3 and 9 h, with only a clear difference in recovery at 12 h — — with roots showing a significant decline at 12 h, while stems and leaves largely returned to control levels. This expression pattern resembles the response characteristics of expansin genes to MeJA in sweet potato and cotton, suggesting that these genes participate in the MeJA-mediated signal transduction pathway in *P. praeruptorum* and exert differential regulatory roles in the growth, development, and stress responses of different vegetative organs. The functional variety of the expansin gene family has been confirmed in multiple plant species; for instance, the sweet potato IbEXP gene participates in tuber development and stress response, whereas *Arabidopsis AtEXPA10* facilitates the growth of leaves and floral organs. In this study, the PpdEXP gene of *P. praeruptorum* showed high structural conservation with homologous genes from other species; its promoter region was rich in stress and hormone response elements, and it demonstrated considerable tissue-specific expression following MeJA treatment, indicating its potential role in the elongation and swelling of *P. praeruptorum* roots, stem thickening, and adaptability to unfavourable conditions. The EXPA subfamily, being the biggest, possesses the highest member count and the greatest concentration of response elements, exhibiting the most pronounced induction effects in roots and stems. It is hypothesized to be the core functional subfamily involved in regulating the morphogenesis of the medicinal part of *P. praeruptorum*, providing candidate targets for subsequent key gene identification.

## 5 Conclusions

This research is the inaugural effort to comprehensively identify 39 expansin genes in *P. praeruptorum* utilising whole-genome data, categorising them into four subfamilies: EXPA, EXPB, EXPLA, and EXPLB, EXPA constitutes the largest subfamily, whilst EXPLA comprises the fewest members. This aligns with the evolutionary conservation of this gene family in terrestrial flora. All PpdEXP proteins are situated in the cell wall and exhibit hydrophilicity. Each contains two conserved domains, DPBB_EXPA_N and Expansin_C, and possesses one to four introns. The gene architectures and conserved motif compositions within the same subfamily exhibit significant similarity, hence affirming the validity of the subfamily classification and the evolutionary conservatism of the family. The 39 genes are distributed across 11 chromosomes, with tandem repeats, segmental duplications, and genome-wide duplication serving as the principal catalysts for the family’s proliferation. The promoter regions are abundant in cis-elements that respond to hormones, light, and stress, with the *EXPA* subfamily exhibiting the greatest quantity of elements. This suggests that their transcription is synergistically regulated by multiple environmental and hormonal signals. The expression profile indicates that PpdEXP genes demonstrate notable tissue specialisation, with certain members exhibiting high expression levels in roots, stems, and leaves, respectively, and marked variations in expression patterns prior to and post-bolting. The exogenous application of MeJA demonstrated that the five candidate genes displayed unique tissue-specific and temporal responses in roots, stems, and leaves, with the majority of genes exhibiting heightened induction in roots and stems. *PpdEXPA01*, *PpdEXPA10*, *PpdEXPA13*, and *PpdEXPB1* were recognised as essential MeJA-responsive genes. These findings systematically elucidate the molecular characteristics, evolutionary patterns, and regulatory mechanisms of the expansin gene family, clarify their pivotal roles in the development of vegetative organs and hormonal responses, and identify essential candidate genes for understanding the growth, development, and stress resistance mechanisms of *P. praeruptorum*.

## Data Availability

The datasets presented in this study can be found in online repositories. The names of the repository/repositories and accession number(s) can be found in the article/**Supplementary Material**.
