# Supplementary material for: Harnessing large-scale genomics-based screening for the identification of expansin genes responsive to abiotic stress in Peucedanum praeruptorum
Source: Front Plant Sci. 2026 Oct 15;17:1983847. doi: 10.3389/fpls.2026.1983847 (PMC13716867; doi:10.3389/fpls.2026.1983847)
Supplement: Supplementary file 1 [file Table1.docx]

**Table S1 Prediction of cis-elements in promoter regions of PpdEXPs**

| **Gene Name** | **Element Name** | **Sequence** | **Starting Point** | **Element Length** | **Function Description** |
| --- | --- | --- | --- | --- | --- |
| PpdEXPA01 | ABRE | CACGTG | 860 | 6 | Abscisic acid responsive element |
| PpdEXPA01 | ABRE | ACGTG | 861 | 5 | Abscisic acid responsive element |
| PpdEXPA01 | ABRE | ACGTG | 869 | 5 | Abscisic acid responsive element |
| PpdEXPA01 | ABRE | ACGTG | 1229 | 5 | Abscisic acid responsive element |
| PpdEXPA01 | AE-box | AGAAACAA | 1098 | 8 | Photoresponsive element |
| PpdEXPA01 | ARE | AAACCA | 1025 | 6 | Abscisic acid responsive element |
| PpdEXPA01 | ARE | AAACCA | 1083 | 6 | Abscisic acid responsive element |
| PpdEXPA01 | ARE | AAACCA | 1378 | 6 | Abscisic acid responsive element |
| PpdEXPA01 | as-1 | TGACG | 321 | 5 | Drought responsive element |
| PpdEXPA01 | as-1 | TGACG | 867 | 5 | Drought responsive element |
| PpdEXPA01 | as-1 | TGACG | 1238 | 5 | Drought responsive element |
| PpdEXPA01 | CGTCA-motif | CGTCA | 321 | 5 | MeJA responsive element |
| PpdEXPA01 | CGTCA-motif | CGTCA | 867 | 5 | MeJA responsive element |
| PpdEXPA01 | CGTCA-motif | CGTCA | 1238 | 5 | MeJA responsive element |
| PpdEXPA01 | ERE | ATTTTAAA | 793 | 8 | Ethylene responsive element |
| PpdEXPA01 | ERE | ATTTTAAA | 1281 | 8 | Ethylene responsive element |
| PpdEXPA01 | GATA-motif | GATAGGA | 393 | 7 | Photoresponsive element |
| PpdEXPA01 | G-box | CACGTG | 860 | 6 | Photoresponsive element |
| PpdEXPA01 | G-box | CACGTC | 868 | 6 | Photoresponsive element |
| PpdEXPA01 | G-box | CACGTC | 1228 | 6 | Photoresponsive element |
| PpdEXPA01 | G-Box | CACGTG | 860 | 6 | Photoresponsive element |
| PpdEXPA01 | LTR | CCGAAA | 1719 | 6 | Low temperature responsive element |
| PpdEXPA01 | STRE | AGGGG | 769 | 5 | Stress Response Element |
| PpdEXPA01 | STRE | AGGGG | 953 | 5 | Stress Response Element |
| PpdEXPA01 | STRE | AGGGG | 1154 | 5 | Stress Response Element |
| PpdEXPA01 | TCT-motif | TCTTAC | 1069 | 6 | Photoresponsive element |
| PpdEXPA01 | TGACG-motif | TGACG | 321 | 5 | MeJA responsive element |
| PpdEXPA01 | TGACG-motif | TGACG | 867 | 5 | MeJA responsive element |
| PpdEXPA01 | TGACG-motif | TGACG | 1238 | 5 | MeJA responsive element |
| PpdEXPA01 | WUN-motif | AAATTACT | 884 | 8 | Wound responsive element |
| PpdEXPA01 | WUN-motif | AAATTACTA | 1496 | 9 | Wound responsive element |
| PpdEXPA02 | ABRE | ACGTG | 1237 | 5 | Abscisic acid responsive element |
| PpdEXPA02 | ABRE | ACGTG | 1756 | 5 | Abscisic acid responsive element |
| PpdEXPA02 | ABRE | ACGTG | 1824 | 5 | Abscisic acid responsive element |
| PpdEXPA02 | ABRE3a | TACGTG | 1236 | 6 | Abscisic acid responsive element |
| PpdEXPA02 | ABRE3a | TACGTG | 1823 | 6 | Abscisic acid responsive element |
| PpdEXPA02 | ABRE4 | CACGTA | 1236 | 6 | Abscisic acid responsive element |
| PpdEXPA02 | ABRE4 | CACGTA | 1823 | 6 | Abscisic acid responsive element |
| PpdEXPA02 | ARE | AAACCA | 1681 | 6 | Abscisic acid responsive element |
| PpdEXPA02 | ARE | AAACCA | 1842 | 6 | Abscisic acid responsive element |
| PpdEXPA02 | as-1 | TGACG | 302 | 5 | Drought responsive element |
| PpdEXPA02 | as-1 | TGACG | 536 | 5 | Drought responsive element |
| PpdEXPA02 | as-1 | TGACG | 891 | 5 | Drought responsive element |
| PpdEXPA02 | Box 4 | ATTAAT | 126 | 6 | Photoresponsive element |
| PpdEXPA02 | CGTCA-motif | CGTCA | 302 | 5 | MeJA responsive element |
| PpdEXPA02 | CGTCA-motif | CGTCA | 536 | 5 | MeJA responsive element |
| PpdEXPA02 | CGTCA-motif | CGTCA | 891 | 5 | MeJA responsive element |
| PpdEXPA02 | GATA-motif | GATAGGG | 487 | 7 | Photoresponsive element |
| PpdEXPA02 | GATA-motif | GATAGGA | 731 | 7 | Photoresponsive element |
| PpdEXPA02 | G-Box | CACGTT | 1756 | 6 | Photoresponsive element |
| PpdEXPA02 | G-box | CCACGTAA | 1235 | 8 | Photoresponsive element |
| PpdEXPA02 | G-box | TACGTG | 1236 | 6 | Photoresponsive element |
| PpdEXPA02 | G-box | TACGTG | 1823 | 6 | Photoresponsive element |
| PpdEXPA02 | I-box | AGATAAGG | 30 | 8 | Photoresponsive element |
| PpdEXPA02 | LTR | CCGAAA | 8 | 6 | Low temperature responsive element |
| PpdEXPA02 | MBS | CAACTG | 1147 | 6 | Drought responsive element |
| PpdEXPA02 | Myb | TAACTG | 37 | 6 | Photoresponsive element |
| PpdEXPA02 | Myb | CAACTG | 1147 | 6 | Photoresponsive element |
| PpdEXPA02 | MYB | CAACAG | 1929 | 6 | Photoresponsive element |
| PpdEXPA02 | MYB recognition site | CCGTTG | 269 | 6 | Photoresponsive element |
| PpdEXPA02 | STRE | AGGGG | 225 | 5 | Stress Response Element |
| PpdEXPA02 | STRE | AGGGG | 486 | 5 | Stress Response Element |
| PpdEXPA02 | STRE | AGGGG | 1368 | 5 | Stress Response Element |
| PpdEXPA02 | STRE | AGGGG | 1449 | 5 | Stress Response Element |
| PpdEXPA02 | TCA | TCATCTTCAT | 773 | 9 | Salicylic acid responsive element |
| PpdEXPA02 | TCA-element | CCATCTTTTT | 976 | 9 | Salicylic acid responsive element |
| PpdEXPA02 | TCT-motif | TCTTAC | 81 | 6 | Photoresponsive element |
| PpdEXPA02 | TCT-motif | TCTTAC | 607 | 6 | Photoresponsive element |
| PpdEXPA02 | TCT-motif | TCTTAC | 1251 | 6 | Photoresponsive element |
| PpdEXPA02 | TGACG-motif | TGACG | 302 | 5 | MeJA responsive element |
| PpdEXPA02 | TGACG-motif | TGACG | 536 | 5 | MeJA responsive element |
| PpdEXPA02 | TGACG-motif | TGACG | 891 | 5 | MeJA responsive element |
| PpdEXPA02 | TGA-element | AACGAC | 881 | 6 | Auxin responsive element |
| PpdEXPA02 | TGA-element | AACGAC | 1210 | 6 | Auxin responsive element |
| PpdEXPA02 | WRE3 | CCACCT | 1387 | 6 | Wound response，Drought responsive element |
| PpdEXPA02 | WUN-motif | CAATTACAT | 1314 | 9 | Wound responsive element |
| PpdEXPA03 | ABRE | ACGTG | 1660 | 5 | Abscisic acid responsive element |
| PpdEXPA03 | ARE | AAACCA | 282 | 6 | Abscisic acid responsive element |
| PpdEXPA03 | as-1 | TGACG | 1634 | 5 | Drought responsive element |
| PpdEXPA03 | Box 4 | ATTAAT | 264 | 6 | Photoresponsive element |
| PpdEXPA03 | Box 4 | ATTAAT | 1805 | 6 | Photoresponsive element |
| PpdEXPA03 | CGTCA-motif | CGTCA | 1634 | 5 | MeJA responsive element |
| PpdEXPA03 | G-Box | CACGTT | 1660 | 6 | Photoresponsive element |
| PpdEXPA03 | GT1-motif | GGTTAA | 1676 | 6 | Photoresponsive element |
| PpdEXPA03 | MBS | CAACTG | 1131 | 6 | Drought responsive element |
| PpdEXPA03 | MBS | CAACTG | 1441 | 6 | Drought responsive element |
| PpdEXPA03 | MYB | CAACCA | 660 | 6 | Photoresponsive element |
| PpdEXPA03 | MYB | CAACCA | 1749 | 6 | Photoresponsive element |
| PpdEXPA03 | Myb | TAACTG | 877 | 6 | Photoresponsive element |
| PpdEXPA03 | Myb | CAACTG | 1131 | 6 | Photoresponsive element |
| PpdEXPA03 | Myb | CAACTG | 1441 | 6 | Photoresponsive element |
| PpdEXPA03 | STRE | AGGGG | 1297 | 5 | Stress Response Element |
| PpdEXPA03 | TC-rich repeats | ATTCTCTAAC | 1371 | 9 | Defense and stress responsive element |
| PpdEXPA03 | TCT-motif | TCTTAC | 1081 | 6 | Photoresponsive element |
| PpdEXPA03 | TGACG-motif | TGACG | 1634 | 5 | MeJA responsive element |
| PpdEXPA03 | TGA-element | AACGAC | 654 | 6 | Auxin responsive element |
| PpdEXPA03 | WUN-motif | AAATTTCTT | 575 | 9 | Wound responsive element |
| PpdEXPA04 | ABRE | CACGTG | 1557 | 6 | Abscisic acid responsive element |
| PpdEXPA04 | ABRE | ACGTG | 1558 | 5 | Abscisic acid responsive element |
| PpdEXPA04 | ABRE | ACGTG | 1722 | 5 | Abscisic acid responsive element |
| PpdEXPA04 | AE-box | AGAAACTT | 983 | 8 | Photoresponsive element |
| PpdEXPA04 | ARE | AAACCA | 268 | 6 | Abscisic acid responsive element |
| PpdEXPA04 | as-1 | TGACG | 910 | 5 | Drought responsive element |
| PpdEXPA04 | Box 4 | ATTAAT | 1862 | 6 | Photoresponsive element |
| PpdEXPA04 | CGTCA-motif | CGTCA | 910 | 5 | MeJA responsive element |
| PpdEXPA04 | ERE | ATTTTAAA | 1473 | 8 | Ethylene responsive element |
| PpdEXPA04 | G-box | CACGTG | 1557 | 6 | Photoresponsive element |
| PpdEXPA04 | G-Box | CACGTG | 1557 | 6 | Photoresponsive element |
| PpdEXPA04 | G-Box | CACGTT | 1722 | 6 | Photoresponsive element |
| PpdEXPA04 | GT1-motif | GGTTAA | 1852 | 6 | Photoresponsive element |
| PpdEXPA04 | GT1-motif | GGTTAA | 1854 | 6 | Photoresponsive element |
| PpdEXPA04 | MBS | CAACTG | 614 | 6 | Drought responsive element |
| PpdEXPA04 | MBS | CAACTG | 1250 | 6 | Drought responsive element |
| PpdEXPA04 | MBS | CAACTG | 1910 | 6 | Drought responsive element |
| PpdEXPA04 | MYB | CAACCA | 1655 | 6 | Photoresponsive element |
| PpdEXPA04 | MYB | TAACCA | 1851 | 6 | Photoresponsive element |
| PpdEXPA04 | MYB | TAACCA | 1855 | 6 | Photoresponsive element |
| PpdEXPA04 | Myb | TAACTG | 102 | 6 | Photoresponsive element |
| PpdEXPA04 | Myb | TAACTG | 137 | 6 | Photoresponsive element |
| PpdEXPA04 | Myb | CAACTG | 614 | 6 | Photoresponsive element |
| PpdEXPA04 | Myb | CAACTG | 1250 | 6 | Photoresponsive element |
| PpdEXPA04 | Myb | CAACTG | 1910 | 6 | Photoresponsive element |
| PpdEXPA04 | MYB recognition site | CCGTTG | 1524 | 6 | Photoresponsive element |
| PpdEXPA04 | STRE | AGGGG | 1571 | 5 | Stress Response Element |
| PpdEXPA04 | TCT-motif | TCTTAC | 373 | 6 | Photoresponsive element |
| PpdEXPA04 | TCT-motif | TCTTAC | 1231 | 6 | Photoresponsive element |
| PpdEXPA04 | TGACG-motif | TGACG | 910 | 5 | MeJA responsive element |
| PpdEXPA04 | W box | TTGACC | 1083 | 6 | Adversity response component，developmental regulation |
| PpdEXPA04 | WUN-motif | AAATTTCTT | 194 | 9 | Wound responsive element |
| PpdEXPA05 | ABRE | ACGTG | 487 | 5 | Abscisic acid responsive element |
| PpdEXPA05 | ABRE | ACGTG | 742 | 5 | Abscisic acid responsive element |
| PpdEXPA05 | ABRE | ACGTG | 1104 | 5 | Abscisic acid responsive element |
| PpdEXPA05 | ABRE | ACGTG | 1309 | 5 | Abscisic acid responsive element |
| PpdEXPA05 | ARE | AAACCA | 807 | 6 | Abscisic acid responsive element |
| PpdEXPA05 | Box 4 | ATTAAT | 272 | 6 | Photoresponsive element |
| PpdEXPA05 | Box 4 | ATTAAT | 363 | 6 | Photoresponsive element |
| PpdEXPA05 | Box 4 | ATTAAT | 673 | 6 | Photoresponsive element |
| PpdEXPA05 | Box 4 | ATTAAT | 829 | 6 | Photoresponsive element |
| PpdEXPA05 | Box 4 | ATTAAT | 853 | 6 | Photoresponsive element |
| PpdEXPA05 | Box 4 | ATTAAT | 1079 | 6 | Photoresponsive element |
| PpdEXPA05 | Box 4 | ATTAAT | 1141 | 6 | Photoresponsive element |
| PpdEXPA05 | Box 4 | ATTAAT | 1181 | 6 | Photoresponsive element |
| PpdEXPA05 | Box 4 | ATTAAT | 1284 | 6 | Photoresponsive element |
| PpdEXPA05 | Box 4 | ATTAAT | 1380 | 6 | Photoresponsive element |
| PpdEXPA05 | Box 4 | ATTAAT | 1429 | 6 | Photoresponsive element |
| PpdEXPA05 | G-Box | CACGTT | 487 | 6 | Photoresponsive element |
| PpdEXPA05 | G-Box | CACGTT | 1104 | 6 | Photoresponsive element |
| PpdEXPA05 | G-Box | CACGTT | 1309 | 6 | Photoresponsive element |
| PpdEXPA05 | G-box | CACGTC | 742 | 6 | Photoresponsive element |
| PpdEXPA05 | GT1-motif | GGTTAAT | 276 | 7 | Photoresponsive element |
| PpdEXPA05 | GT1-motif | GGTTAA | 277 | 6 | Photoresponsive element |
| PpdEXPA05 | I-box | TGATAATGT | 1058 | 9 | Photoresponsive element |
| PpdEXPA05 | MBS | CAACTG | 999 | 6 | Drought responsive element |
| PpdEXPA05 | Myb | CAACTG | 999 | 6 | Photoresponsive element |
| PpdEXPA05 | MYB | CAACCA | 160 | 6 | Photoresponsive element |
| PpdEXPA05 | MYB | TAACCA | 756 | 6 | Photoresponsive element |
| PpdEXPA05 | MYB | TAACCA | 943 | 6 | Photoresponsive element |
| PpdEXPA05 | MYB recognition site | CCGTTG | 1612 | 6 | Photoresponsive element |
| PpdEXPA05 | STRE | AGGGG | 1892 | 5 | Stress Response Element |
| PpdEXPA05 | TCA-element | CCATCTTTTT | 309 | 9 | Salicylic acid responsive element |
| PpdEXPA05 | TCA-element | CCATCTTTTT | 1365 | 9 | Salicylic acid responsive element |
| PpdEXPA05 | TC-rich repeats | ATTCTCTAAC | 188 | 9 | Defense and stress responsive element |
| PpdEXPA05 | TCT-motif | TCTTAC | 1336 | 6 | Photoresponsive element |
| PpdEXPA05 | W box | TTGACC | 1883 | 6 | Adversity response component，developmental regulation |
| PpdEXPA05 | WRE3 | CCACCT | 753 | 6 | Wound response，Drought responsive element |
| PpdEXPA06 | AE-box | AGAAACAA | 1869 | 8 | Photoresponsive element |
| PpdEXPA06 | as-1 | TGACG | 519 | 5 | Drought responsive element |
| PpdEXPA06 | as-1 | TGACG | 1083 | 5 | Drought responsive element |
| PpdEXPA06 | Box 4 | ATTAAT | 1120 | 6 | Photoresponsive element |
| PpdEXPA06 | Box 4 | ATTAAT | 1139 | 6 | Photoresponsive element |
| PpdEXPA06 | Box 4 | ATTAAT | 1151 | 6 | Photoresponsive element |
| PpdEXPA06 | Box 4 | ATTAAT | 1229 | 6 | Photoresponsive element |
| PpdEXPA06 | Box 4 | ATTAAT | 1256 | 6 | Photoresponsive element |
| PpdEXPA06 | Box 4 | ATTAAT | 1275 | 6 | Photoresponsive element |
| PpdEXPA06 | Box 4 | ATTAAT | 1286 | 6 | Photoresponsive element |
| PpdEXPA06 | Box 4 | ATTAAT | 1482 | 6 | Photoresponsive element |
| PpdEXPA06 | Box 4 | ATTAAT | 1492 | 6 | Photoresponsive element |
| PpdEXPA06 | Box 4 | ATTAAT | 1539 | 6 | Photoresponsive element |
| PpdEXPA06 | Box 4 | ATTAAT | 1618 | 6 | Photoresponsive element |
| PpdEXPA06 | Box 4 | ATTAAT | 1630 | 6 | Photoresponsive element |
| PpdEXPA06 | Box 4 | ATTAAT | 1643 | 6 | Photoresponsive element |
| PpdEXPA06 | Box 4 | ATTAAT | 1660 | 6 | Photoresponsive element |
| PpdEXPA06 | CGTCA-motif | CGTCA | 519 | 5 | MeJA responsive element |
| PpdEXPA06 | CGTCA-motif | CGTCA | 1083 | 5 | MeJA responsive element |
| PpdEXPA06 | ERE | ATTTTAAA | 1557 | 8 | Ethylene responsive element |
| PpdEXPA06 | MBS | CAACTG | 1371 | 6 | Drought responsive element |
| PpdEXPA06 | Myb | CAACTG | 1371 | 6 | Photoresponsive element |
| PpdEXPA06 | Myb | TAACTG | 1384 | 6 | Photoresponsive element |
| PpdEXPA06 | MYB | CAACCA | 705 | 6 | Photoresponsive element |
| PpdEXPA06 | MYB | CAACCA | 840 | 6 | Photoresponsive element |
| PpdEXPA06 | MYB | TAACCA | 1064 | 6 | Photoresponsive element |
| PpdEXPA06 | MYB | CAACCA | 1070 | 6 | Photoresponsive element |
| PpdEXPA06 | MYB | TAACCA | 1455 | 6 | Photoresponsive element |
| PpdEXPA06 | MYB | TAACCA | 1510 | 6 | Photoresponsive element |
| PpdEXPA06 | MYB | CAACCA | 1710 | 6 | Photoresponsive element |
| PpdEXPA06 | MYB | CAACAG | 1941 | 6 | Photoresponsive element |
| PpdEXPA06 | STRE | AGGGG | 533 | 5 | Stress Response Element |
| PpdEXPA06 | STRE | AGGGG | 866 | 5 | Stress Response Element |
| PpdEXPA06 | STRE | AGGGG | 1390 | 5 | Stress Response Element |
| PpdEXPA06 | TCT-motif | TCTTAC | 1971 | 6 | Photoresponsive element |
| PpdEXPA06 | TGACG-motif | TGACG | 519 | 5 | MeJA responsive element |
| PpdEXPA06 | TGACG-motif | TGACG | 1083 | 5 | MeJA responsive element |
| PpdEXPA06 | TGA-element | AACGAC | 41 | 6 | Auxin responsive element |
| PpdEXPA06 | TGA-element | AACGAC | 65 | 6 | Auxin responsive element |
| PpdEXPA06 | WRE3 | CCACCT | 1935 | 6 | Wound response，Drought responsive element |
| PpdEXPA06 | WUN-motif | AAATTACTA | 1436 | 9 | Wound responsive element |
| PpdEXPA07 | ABRE | ACGTG | 1310 | 5 | Abscisic acid responsive element |
| PpdEXPA07 | ABRE | CACGTG | 1319 | 6 | Abscisic acid responsive element |
| PpdEXPA07 | ABRE | ACGTG | 1320 | 5 | Abscisic acid responsive element |
| PpdEXPA07 | ARE | AAACCA | 1561 | 6 | Abscisic acid responsive element |
| PpdEXPA07 | ARE | AAACCA | 1692 | 6 | Abscisic acid responsive element |
| PpdEXPA07 | ARE | AAACCA | 1920 | 6 | Abscisic acid responsive element |
| PpdEXPA07 | as-1 | TGACG | 443 | 5 | Drought responsive element |
| PpdEXPA07 | as-1 | TGACG | 1290 | 5 | Drought responsive element |
| PpdEXPA07 | as-1 | TGACG | 1941 | 5 | Drought responsive element |
| PpdEXPA07 | Box 4 | ATTAAT | 519 | 6 | Photoresponsive element |
| PpdEXPA07 | Box 4 | ATTAAT | 1726 | 6 | Photoresponsive element |
| PpdEXPA07 | CGTCA-motif | CGTCA | 443 | 5 | MeJA responsive element |
| PpdEXPA07 | CGTCA-motif | CGTCA | 1290 | 5 | MeJA responsive element |
| PpdEXPA07 | CGTCA-motif | CGTCA | 1941 | 5 | MeJA responsive element |
| PpdEXPA07 | ERE | ATTTTAAA | 378 | 8 | Ethylene responsive element |
| PpdEXPA07 | GATA-motif | GATAGGG | 896 | 7 | Photoresponsive element |
| PpdEXPA07 | GATA-motif | GATAGGA | 1215 | 7 | Photoresponsive element |
| PpdEXPA07 | G-Box | CACGTT | 1310 | 6 | Photoresponsive element |
| PpdEXPA07 | G-Box | CACGTG | 1319 | 6 | Photoresponsive element |
| PpdEXPA07 | G-box | CACGTG | 1319 | 6 | Photoresponsive element |
| PpdEXPA07 | GT1-motif | GGTTAAT | 1007 | 7 | Photoresponsive element |
| PpdEXPA07 | GT1-motif | GGTTAA | 1344 | 6 | Photoresponsive element |
| PpdEXPA07 | I-box | gGATAAGGTG | 1924 | 9 | Photoresponsive element |
| PpdEXPA07 | LTR | CCGAAA | 495 | 6 | Low temperature responsive element |
| PpdEXPA07 | MBS | CAACTG | 769 | 6 | Drought responsive element |
| PpdEXPA07 | MBS | CAACTG | 846 | 6 | Drought responsive element |
| PpdEXPA07 | Myb | CAACTG | 769 | 6 | Photoresponsive element |
| PpdEXPA07 | Myb | TAACTG | 797 | 6 | Photoresponsive element |
| PpdEXPA07 | Myb | CAACTG | 846 | 6 | Photoresponsive element |
| PpdEXPA07 | MYB | CAACCA | 985 | 6 | Photoresponsive element |
| PpdEXPA07 | MYB | TAACCA | 1006 | 6 | Photoresponsive element |
| PpdEXPA07 | MYB | CAACCA | 1162 | 6 | Photoresponsive element |
| PpdEXPA07 | MYB | TAACCA | 1343 | 6 | Photoresponsive element |
| PpdEXPA07 | MYB | CAACAG | 1826 | 6 | Photoresponsive element |
| PpdEXPA07 | TGACG-motif | TGACG | 443 | 5 | MeJA responsive element |
| PpdEXPA07 | TGACG-motif | TGACG | 1290 | 5 | MeJA responsive element |
| PpdEXPA07 | TGACG-motif | TGACG | 1941 | 5 | MeJA responsive element |
| PpdEXPA07 | WRE3 | CCACCT | 1923 | 6 | Wound response，Drought responsive element |
| PpdEXPA07 | WUN-motif | AAATTTCTT | 1274 | 9 | Wound responsive element |
| PpdEXPA07 | WUN-motif | AAATTTCTT | 1768 | 9 | Wound responsive element |
| PpdEXPA08 | ABRE | ACGTG | 1895 | 5 | Abscisic acid responsive element |
| PpdEXPA08 | AE-box | AGAAACAA | 1454 | 8 | Photoresponsive element |
| PpdEXPA08 | ARE | AAACCA | 642 | 6 | Abscisic acid responsive element |
| PpdEXPA08 | ARE | AAACCA | 1277 | 6 | Abscisic acid responsive element |
| PpdEXPA08 | ARE | AAACCA | 1888 | 6 | Abscisic acid responsive element |
| PpdEXPA08 | as-1 | TGACG | 686 | 5 | Drought responsive element |
| PpdEXPA08 | Box 4 | ATTAAT | 104 | 6 | Photoresponsive element |
| PpdEXPA08 | Box 4 | ATTAAT | 1840 | 6 | Photoresponsive element |
| PpdEXPA08 | CGTCA-motif | CGTCA | 686 | 5 | MeJA responsive element |
| PpdEXPA08 | ERE | ATTTTAAA | 144 | 8 | Ethylene responsive element |
| PpdEXPA08 | G-Box | CACGTT | 1895 | 6 | Photoresponsive element |
| PpdEXPA08 | GT1-motif | GTGTGTGAA | 1104 | 9 | Photoresponsive element |
| PpdEXPA08 | MYB | CAACAG | 882 | 6 | Photoresponsive element |
| PpdEXPA08 | MYB | CAACAG | 1176 | 6 | Photoresponsive element |
| PpdEXPA08 | MYB | CAACCA | 1228 | 6 | Photoresponsive element |
| PpdEXPA08 | MYB | CAACAG | 1868 | 6 | Photoresponsive element |
| PpdEXPA08 | Myb | TAACTG | 1009 | 6 | Photoresponsive element |
| PpdEXPA08 | Myb | TAACTG | 1209 | 6 | Photoresponsive element |
| PpdEXPA08 | STRE | AGGGG | 634 | 5 | Stress Response Element |
| PpdEXPA08 | STRE | AGGGG | 1446 | 5 | Stress Response Element |
| PpdEXPA08 | TCA | TCATCTTCAT | 613 | 10 | Salicylic acid responsive element |
| PpdEXPA08 | TCA-element | CCATCTTTTT | 559 | 9 | Salicylic acid responsive element |
| PpdEXPA08 | TCT-motif | TCTTAC | 77 | 6 | Photoresponsive element |
| PpdEXPA08 | TGACG-motif | TGACG | 686 | 5 | MeJA responsive element |
| PpdEXPA08 | WUN-motif | AAATTTCTT | 1668 | 9 | Wound responsive element |
| PpdEXPA09 | ABRE | ACGTG | 1763 | 5 | Abscisic acid responsive element |
| PpdEXPA09 | Box 4 | ATTAAT | 181 | 6 | Photoresponsive element |
| PpdEXPA09 | Box 4 | ATTAAT | 663 | 6 | Photoresponsive element |
| PpdEXPA09 | Box 4 | ATTAAT | 901 | 6 | Photoresponsive element |
| PpdEXPA09 | GATA-motif | AAGATAAGATT | 947 | 10 | Photoresponsive element |
| PpdEXPA09 | G-Box | CACGTT | 1762 | 6 | Photoresponsive element |
| PpdEXPA09 | GT1-motif | GGTTAA | 1251 | 6 | Photoresponsive element |
| PpdEXPA09 | GT1-motif | GGTTAA | 1537 | 6 | Photoresponsive element |
| PpdEXPA09 | GT1-motif | GGTTAAT | 1549 | 7 | Photoresponsive element |
| PpdEXPA09 | MBS | CAACTG | 1379 | 6 | Drought responsive element |
| PpdEXPA09 | MYB | CAACAG | 687 | 6 | Photoresponsive element |
| PpdEXPA09 | MYB | CAACAG | 1217 | 6 | Photoresponsive element |
| PpdEXPA09 | MYB | TAACCA | 1252 | 6 | Photoresponsive element |
| PpdEXPA09 | MYB | TAACCA | 1538 | 6 | Photoresponsive element |
| PpdEXPA09 | MYB | CAACCA | 1875 | 6 | Photoresponsive element |
| PpdEXPA09 | Myb | TAACTG | 1373 | 6 | Photoresponsive element |
| PpdEXPA09 | Myb | CAACTG | 1379 | 6 | Photoresponsive element |
| PpdEXPA09 | STRE | AGGGG | 1247 | 5 | Stress Response Element |
| PpdEXPA09 | STRE | AGGGG | 1604 | 5 | Stress Response Element |
| PpdEXPA09 | TCA-element | CCATCTTTTT | 605 | 9 | Salicylic acid responsive element |
| PpdEXPA09 | TCT-motif | TCTTAC | 81 | 6 | Photoresponsive element |
| PpdEXPA09 | TCT-motif | TCTTAC | 752 | 6 | Photoresponsive element |
| PpdEXPA09 | TCT-motif | TCTTAC | 1757 | 6 | Photoresponsive element |
| PpdEXPA09 | WRE3 | CCACCT | 1872 | 6 | Wound response，Drought responsive element |
| PpdEXPA10 | ABRE | ACGTG | 1771 | 5 | Abscisic acid responsive element |
| PpdEXPA10 | ARE | AAACCA | 60 | 6 | Abscisic acid responsive element |
| PpdEXPA10 | ARE | AAACCA | 434 | 6 | Abscisic acid responsive element |
| PpdEXPA10 | ARE | AAACCA | 1504 | 6 | Abscisic acid responsive element |
| PpdEXPA10 | as-1 | TGACG | 4 | 5 | Drought responsive element |
| PpdEXPA10 | as-1 | TGACG | 624 | 5 | Drought responsive element |
| PpdEXPA10 | as-1 | TGACG | 1002 | 5 | Drought responsive element |
| PpdEXPA10 | as-1 | TGACG | 1483 | 5 | Drought responsive element |
| PpdEXPA10 | Box 4 | ATTAAT | 713 | 6 | Photoresponsive element |
| PpdEXPA10 | Box 4 | ATTAAT | 1375 | 6 | Photoresponsive element |
| PpdEXPA10 | CGTCA-motif | CGTCA | 4 | 5 | MeJA responsive element |
| PpdEXPA10 | CGTCA-motif | CGTCA | 624 | 5 | MeJA responsive element |
| PpdEXPA10 | CGTCA-motif | CGTCA | 1002 | 5 | MeJA responsive element |
| PpdEXPA10 | CGTCA-motif | CGTCA | 1483 | 5 | MeJA responsive element |
| PpdEXPA10 | G-Box | CACGTT | 1770 | 6 | Photoresponsive element |
| PpdEXPA10 | I-box | AAGATAAGGCT | 785 | 10 | Photoresponsive element |
| PpdEXPA10 | MBS | CAACTG | 564 | 6 | Drought responsive element |
| PpdEXPA10 | Myb | CAACTG | 564 | 6 | Photoresponsive element |
| PpdEXPA10 | MYB | CAACCA | 240 | 6 | Photoresponsive element |
| PpdEXPA10 | MYB | CAACCA | 503 | 6 | Photoresponsive element |
| PpdEXPA10 | MYB | CAACCA | 1689 | 6 | Photoresponsive element |
| PpdEXPA10 | STRE | AGGGG | 1673 | 5 | Stress Response Element |
| PpdEXPA10 | TGACG-motif | TGACG | 4 | 5 | MeJA responsive element |
| PpdEXPA10 | TGACG-motif | TGACG | 624 | 5 | MeJA responsive element |
| PpdEXPA10 | TGACG-motif | TGACG | 1002 | 5 | MeJA responsive element |
| PpdEXPA10 | TGACG-motif | TGACG | 1483 | 5 | MeJA responsive element |
| PpdEXPA10 | W box | TTGACC | 872 | 6 | Adversity response component，developmental regulation |
| PpdEXPA11 | ABRE | TACGTGTC | 699 | 8 | Abscisic acid responsive element |
| PpdEXPA11 | ABRE | ACGTG | 701 | 5 | Abscisic acid responsive element |
| PpdEXPA11 | ABRE3a | TACGTG | 701 | 6 | Abscisic acid responsive element |
| PpdEXPA11 | ABRE4 | CACGTA | 701 | 6 | Abscisic acid responsive element |
| PpdEXPA11 | Box 4 | ATTAAT | 864 | 6 | Photoresponsive element |
| PpdEXPA11 | ERE | ATTTTAAA | 93 | 8 | Ethylene responsive element |
| PpdEXPA11 | ERE | ATTTTAAA | 231 | 8 | Ethylene responsive element |
| PpdEXPA11 | ERE | ATTTTAAA | 320 | 8 | Ethylene responsive element |
| PpdEXPA11 | ERE | ATTTTAAA | 1260 | 8 | Ethylene responsive element |
| PpdEXPA11 | G-box | TACGTG | 701 | 6 | Photoresponsive element |
| PpdEXPA11 | GT1-motif | GGTTAA | 1950 | 6 | Photoresponsive element |
| PpdEXPA11 | Myb | TAACTG | 617 | 6 | Photoresponsive element |
| PpdEXPA11 | STRE | AGGGG | 249 | 5 | Stress Response Element |
| PpdEXPA11 | STRE | AGGGG | 1036 | 5 | Stress Response Element |
| PpdEXPA12 | ABRE | ACGTG | 113 | 5 | Abscisic acid responsive element |
| PpdEXPA12 | ABRE | ACGTG | 545 | 5 | Abscisic acid responsive element |
| PpdEXPA12 | ABRE | CACGTG | 1149 | 6 | Abscisic acid responsive element |
| PpdEXPA12 | ABRE | ACGTG | 1150 | 5 | Abscisic acid responsive element |
| PpdEXPA12 | ABRE3a | TACGTG | 113 | 6 | Abscisic acid responsive element |
| PpdEXPA12 | ABRE4 | CACGTA | 113 | 6 | Abscisic acid responsive element |
| PpdEXPA12 | ARE | AAACCA | 44 | 6 | Abscisic acid responsive element |
| PpdEXPA12 | ARE | AAACCA | 87 | 6 | Abscisic acid responsive element |
| PpdEXPA12 | as-1 | TGACG | 400 | 5 | Drought responsive element |
| PpdEXPA12 | Box 4 | ATTAAT | 177 | 6 | Photoresponsive element |
| PpdEXPA12 | Box 4 | ATTAAT | 630 | 6 | Photoresponsive element |
| PpdEXPA12 | Box 4 | ATTAAT | 634 | 6 | Photoresponsive element |
| PpdEXPA12 | Box 4 | ATTAAT | 715 | 6 | Photoresponsive element |
| PpdEXPA12 | Box 4 | ATTAAT | 1140 | 6 | Photoresponsive element |
| PpdEXPA12 | Box 4 | ATTAAT | 1194 | 6 | Photoresponsive element |
| PpdEXPA12 | CGTCA-motif | CGTCA | 400 | 5 | MeJA responsive element |
| PpdEXPA12 | ERE | ATTTTAAA | 1505 | 8 | Ethylene responsive element |
| PpdEXPA12 | G-box | TACGTG | 113 | 6 | Photoresponsive element |
| PpdEXPA12 | G-box | CACGTG | 1149 | 6 | Photoresponsive element |
| PpdEXPA12 | G-box | CACGAC | 1631 | 6 | Photoresponsive element |
| PpdEXPA12 | G-Box | CACGTT | 544 | 6 | Photoresponsive element |
| PpdEXPA12 | G-Box | CACGTG | 1149 | 6 | Photoresponsive element |
| PpdEXPA12 | GT1-motif | GTGTGTGAA | 1850 | 9 | Photoresponsive element |
| PpdEXPA12 | LTR | CCGAAA | 1547 | 6 | Low temperature responsive element |
| PpdEXPA12 | LTR | CCGAAA | 1614 | 6 | Low temperature responsive element |
| PpdEXPA12 | TCA | TCATCTTCAT | 518 | 9 | Salicylic acid responsive element |
| PpdEXPA12 | TCA-element | CCATCTTTTT | 912 | 9 | Salicylic acid responsive element |
| PpdEXPA12 | TCT-motif | TCTTAC | 160 | 6 | Photoresponsive element |
| PpdEXPA12 | TCT-motif | TCTTAC | 1519 | 6 | Photoresponsive element |
| PpdEXPA12 | TGACG-motif | TGACG | 400 | 5 | MeJA responsive element |
| PpdEXPA12 | W box | TTGACC | 1705 | 6 | Adversity response component，developmental regulation |
| PpdEXPA12 | WUN-motif | AAATTACT | 798 | 8 | Wound responsive element |
| PpdEXPA12 | WUN-motif | TTATTACAT | 1715 | 9 | Wound responsive element |
| PpdEXPA13 | ABRE | ACGTG | 88 | 5 | Abscisic acid responsive element |
| PpdEXPA13 | ABRE | ACGTG | 111 | 5 | Abscisic acid responsive element |
| PpdEXPA13 | ABRE | ACGTG | 798 | 5 | Abscisic acid responsive element |
| PpdEXPA13 | ABRE | ACGTG | 1261 | 5 | Abscisic acid responsive element |
| PpdEXPA13 | ABRE3a | TACGTG | 1260 | 6 | Abscisic acid responsive element |
| PpdEXPA13 | ABRE4 | CACGTA | 1260 | 6 | Abscisic acid responsive element |
| PpdEXPA13 | AE-box | AGAAACAA | 1047 | 8 | Photoresponsive element |
| PpdEXPA13 | ARE | AAACCA | 923 | 6 | Abscisic acid responsive element |
| PpdEXPA13 | ARE | AAACCA | 1457 | 6 | Abscisic acid responsive element |
| PpdEXPA13 | as-1 | TGACG | 86 | 5 | Drought responsive element |
| PpdEXPA13 | as-1 | TGACG | 109 | 5 | Drought responsive element |
| PpdEXPA13 | as-1 | TGACG | 787 | 5 | Drought responsive element |
| PpdEXPA13 | as-1 | TGACG | 790 | 5 | Drought responsive element |
| PpdEXPA13 | as-1 | TGACG | 800 | 5 | Drought responsive element |
| PpdEXPA13 | Box 4 | ATTAAT | 32 | 6 | Photoresponsive element |
| PpdEXPA13 | Box 4 | ATTAAT | 338 | 6 | Photoresponsive element |
| PpdEXPA13 | Box 4 | ATTAAT | 363 | 6 | Photoresponsive element |
| PpdEXPA13 | Box 4 | ATTAAT | 415 | 6 | Photoresponsive element |
| PpdEXPA13 | Box 4 | ATTAAT | 431 | 6 | Photoresponsive element |
| PpdEXPA13 | Box 4 | ATTAAT | 584 | 6 | Photoresponsive element |
| PpdEXPA13 | Box 4 | ATTAAT | 588 | 6 | Photoresponsive element |
| PpdEXPA13 | Box 4 | ATTAAT | 592 | 6 | Photoresponsive element |
| PpdEXPA13 | Box 4 | ATTAAT | 596 | 6 | Photoresponsive element |
| PpdEXPA13 | Box 4 | ATTAAT | 1589 | 6 | Photoresponsive element |
| PpdEXPA13 | Box 4 | ATTAAT | 1708 | 6 | Photoresponsive element |
| PpdEXPA13 | CGTCA-motif | CGTCA | 86 | 5 | MeJA responsive element |
| PpdEXPA13 | CGTCA-motif | CGTCA | 109 | 5 | MeJA responsive element |
| PpdEXPA13 | CGTCA-motif | CGTCA | 787 | 5 | MeJA responsive element |
| PpdEXPA13 | CGTCA-motif | CGTCA | 790 | 5 | MeJA responsive element |
| PpdEXPA13 | CGTCA-motif | CGTCA | 800 | 5 | MeJA responsive element |
| PpdEXPA13 | ERE | ATTTTAAA | 45 | 8 | Ethylene responsive element |
| PpdEXPA13 | ERE | ATTTTAAA | 450 | 8 | Ethylene responsive element |
| PpdEXPA13 | ERE | ATTTTAAA | 1030 | 8 | Ethylene responsive element |
| PpdEXPA13 | ERE | ATTTTAAA | 1637 | 8 | Ethylene responsive element |
| PpdEXPA13 | ERE | ATTTTAAA | 1684 | 8 | Ethylene responsive element |
| PpdEXPA13 | G-box | CACGTC | 87 | 6 | Photoresponsive element |
| PpdEXPA13 | G-box | CACGTC | 110 | 6 | Photoresponsive element |
| PpdEXPA13 | G-box | CACGTC | 798 | 6 | Photoresponsive element |
| PpdEXPA13 | G-box | TACGTG | 1260 | 6 | Photoresponsive element |
| PpdEXPA13 | LTR | CCGAAA | 1172 | 6 | Low temperature responsive element |
| PpdEXPA13 | LTR | CCGAAA | 1408 | 6 | Low temperature responsive element |
| PpdEXPA13 | MBS | CAACTG | 654 | 6 | Drought responsive element |
| PpdEXPA13 | Myb | CAACTG | 654 | 6 | Photoresponsive element |
| PpdEXPA13 | MYB | TAACCA | 1264 | 6 | Photoresponsive element |
| PpdEXPA13 | STRE | AGGGG | 973 | 5 | Stress Response Element |
| PpdEXPA13 | STRE | AGGGG | 1356 | 5 | Stress Response Element |
| PpdEXPA13 | STRE | AGGGG | 1508 | 5 | Stress Response Element |
| PpdEXPA13 | STRE | AGGGG | 1795 | 5 | Stress Response Element |
| PpdEXPA13 | STRE | AGGGG | 1856 | 5 | Stress Response Element |
| PpdEXPA13 | STRE | AGGGG | 1908 | 5 | Stress Response Element |
| PpdEXPA13 | TC-rich repeats | GTTTTCTTAC | 1574 | 9 | Defense and stress responsive element |
| PpdEXPA13 | TCT-motif | TCTTAC | 556 | 6 | Photoresponsive element |
| PpdEXPA13 | TGACG-motif | TGACG | 86 | 5 | MeJA responsive element |
| PpdEXPA13 | TGACG-motif | TGACG | 109 | 5 | MeJA responsive element |
| PpdEXPA13 | TGACG-motif | TGACG | 787 | 5 | MeJA responsive element |
| PpdEXPA13 | TGACG-motif | TGACG | 790 | 5 | MeJA responsive element |
| PpdEXPA13 | TGACG-motif | TGACG | 800 | 5 | MeJA responsive element |
| PpdEXPA13 | TGA-element | AACGAC | 1160 | 6 | Auxin responsive element |
| PpdEXPA13 | W box | TTGACC | 1148 | 6 | Adversity response component，developmental regulation |
| PpdEXPA13 | WRE3 | CCACCT | 99 | 6 | Wound response，Drought responsive element |
| PpdEXPA13 | WRE3 | CCACCT | 885 | 6 | Wound response，Drought responsive element |
| PpdEXPA14 | ABRE | ACGTG | 58 | 5 | Abscisic acid responsive element |
| PpdEXPA14 | ABRE | ACGTG | 334 | 5 | Abscisic acid responsive element |
| PpdEXPA14 | ABRE | ACGTG | 356 | 5 | Abscisic acid responsive element |
| PpdEXPA14 | ABRE | ACGTG | 563 | 5 | Abscisic acid responsive element |
| PpdEXPA14 | ABRE | ACGTG | 848 | 5 | Abscisic acid responsive element |
| PpdEXPA14 | ABRE3a | TACGTG | 58 | 6 | Abscisic acid responsive element |
| PpdEXPA14 | ABRE3a | TACGTG | 356 | 6 | Abscisic acid responsive element |
| PpdEXPA14 | ABRE3a | TACGTG | 562 | 6 | Abscisic acid responsive element |
| PpdEXPA14 | ABRE3a | TACGTG | 848 | 6 | Abscisic acid responsive element |
| PpdEXPA14 | ABRE4 | CACGTA | 58 | 6 | Abscisic acid responsive element |
| PpdEXPA14 | ABRE4 | CACGTA | 356 | 6 | Abscisic acid responsive element |
| PpdEXPA14 | ABRE4 | CACGTA | 562 | 6 | Abscisic acid responsive element |
| PpdEXPA14 | ABRE4 | CACGTA | 848 | 6 | Abscisic acid responsive element |
| PpdEXPA14 | ARE | AAACCA | 306 | 6 | Abscisic acid responsive element |
| PpdEXPA14 | as-1 | TGACG | 368 | 5 | Drought responsive element |
| PpdEXPA14 | as-1 | TGACG | 1452 | 5 | Drought responsive element |
| PpdEXPA14 | Box 4 | ATTAAT | 216 | 6 | Photoresponsive element |
| PpdEXPA14 | Box 4 | ATTAAT | 1611 | 6 | Photoresponsive element |
| PpdEXPA14 | CGTCA-motif | CGTCA | 368 | 5 | MeJA responsive element |
| PpdEXPA14 | CGTCA-motif | CGTCA | 1452 | 5 | MeJA responsive element |
| PpdEXPA14 | ERE | ATTTCATA | 1241 | 8 | Ethylene responsive element |
| PpdEXPA14 | ERE | ATTTCATA | 1434 | 8 | Ethylene responsive element |
| PpdEXPA14 | ERE | ATTTTAAA | 1581 | 8 | Ethylene responsive element |
| PpdEXPA14 | G-box | TACGTG | 58 | 6 | Photoresponsive element |
| PpdEXPA14 | G-box | TACGTG | 356 | 6 | Photoresponsive element |
| PpdEXPA14 | G-box | TACGTG | 562 | 6 | Photoresponsive element |
| PpdEXPA14 | G-box | TACGTG | 848 | 6 | Photoresponsive element |
| PpdEXPA14 | G-Box | CACGTT | 334 | 6 | Photoresponsive element |
| PpdEXPA14 | GT1-motif | GGTTAA | 1038 | 6 | Photoresponsive element |
| PpdEXPA14 | MBS | CAACTG | 752 | 6 | Drought responsive element |
| PpdEXPA14 | Myb | TAACTG | 688 | 6 | Photoresponsive element |
| PpdEXPA14 | Myb | CAACTG | 752 | 6 | Photoresponsive element |
| PpdEXPA14 | Myb | TAACTG | 891 | 6 | Photoresponsive element |
| PpdEXPA14 | MYB | TAACCA | 326 | 6 | Photoresponsive element |
| PpdEXPA14 | MYB | TAACCA | 1039 | 6 | Photoresponsive element |
| PpdEXPA14 | MYB | CAACCA | 1346 | 6 | Photoresponsive element |
| PpdEXPA14 | MYB recognition site | CCGTTG | 1148 | 6 | Photoresponsive element |
| PpdEXPA14 | STRE | AGGGG | 47 | 5 | Stress Response Element |
| PpdEXPA14 | STRE | AGGGG | 398 | 5 | Stress Response Element |
| PpdEXPA14 | STRE | AGGGG | 779 | 5 | Stress Response Element |
| PpdEXPA14 | TCA | TCATCTTCAT | 1526 | 9 | Salicylic acid responsive element |
| PpdEXPA14 | TC-rich repeats | GTTTTCTTAC | 841 | 9 | Defense and stress responsive element |
| PpdEXPA14 | TCT-motif | TCTTAC | 824 | 6 | Photoresponsive element |
| PpdEXPA14 | TCT-motif | TCTTAC | 841 | 6 | Photoresponsive element |
| PpdEXPA14 | TGACG-motif | TGACG | 368 | 5 | MeJA responsive element |
| PpdEXPA14 | TGACG-motif | TGACG | 1452 | 5 | MeJA responsive element |
| PpdEXPA14 | TGA-element | AACGAC | 1126 | 6 | Auxin responsive element |
| PpdEXPA14 | WRE3 | CCACCT | 323 | 6 | Wound response，Drought responsive element |
| PpdEXPA14 | WUN-motif | AAATTACT | 946 | 8 | Wound responsive element |
| PpdEXPA15 | ABRE | AACCCGG | 1094 | 7 | Abscisic acid responsive element |
| PpdEXPA15 | AE-box | AGAAACAA | 1965 | 8 | Photoresponsive element |
| PpdEXPA15 | ARE | AAACCA | 160 | 6 | Abscisic acid responsive element |
| PpdEXPA15 | ARE | AAACCA | 170 | 6 | Abscisic acid responsive element |
| PpdEXPA15 | ARE | AAACCA | 1010 | 6 | Abscisic acid responsive element |
| PpdEXPA15 | Box 4 | ATTAAT | 585 | 6 | Photoresponsive element |
| PpdEXPA15 | Box 4 | ATTAAT | 885 | 6 | Photoresponsive element |
| PpdEXPA15 | Box 4 | ATTAAT | 1706 | 6 | Photoresponsive element |
| PpdEXPA15 | ERE | ATTTCATA | 1243 | 8 | Ethylene responsive element |
| PpdEXPA15 | ERE | ATTTTAAA | 1679 | 8 | Ethylene responsive element |
| PpdEXPA15 | GT1-motif | GGTTAAT | 1211 | 7 | Photoresponsive element |
| PpdEXPA15 | GT1-motif | GGTTAA | 1458 | 6 | Photoresponsive element |
| PpdEXPA15 | LTR | CCGAAA | 389 | 6 | Low temperature responsive element |
| PpdEXPA15 | LTR | CCGAAA | 1848 | 6 | Low temperature responsive element |
| PpdEXPA15 | STRE | AGGGG | 1590 | 5 | Stress Response Element |
| PpdEXPA15 | STRE | AGGGG | 1892 | 5 | Stress Response Element |
| PpdEXPA15 | TC-rich repeats | GTTTTCTTAC | 689 | 9 | Defense and stress responsive element |
| PpdEXPA15 | W box | TTGACC | 1327 | 6 | Adversity response component，developmental regulation |
| PpdEXPA15 | W box | TTGACC | 1347 | 6 | Adversity response component，developmental regulation |
| PpdEXPA15 | W box | TTGACC | 1619 | 6 | Adversity response component，developmental regulation |
| PpdEXPA15 | W box | TTGACC | 1774 | 6 | Adversity response component，developmental regulation |
| PpdEXPA16 | ABRE | CACGTG | 578 | 6 | Abscisic acid responsive element |
| PpdEXPA16 | ABRE | ACGTG | 579 | 5 | Abscisic acid responsive element |
| PpdEXPA16 | ABRE | ACGTG | 740 | 5 | Abscisic acid responsive element |
| PpdEXPA16 | ARE | AAACCA | 596 | 6 | Abscisic acid responsive element |
| PpdEXPA16 | ARE | AAACCA | 1179 | 6 | Abscisic acid responsive element |
| PpdEXPA16 | as-1 | TGACG | 738 | 5 | Drought responsive element |
| PpdEXPA16 | Box 4 | ATTAAT | 931 | 6 | Photoresponsive element |
| PpdEXPA16 | CGTCA-motif | CGTCA | 738 | 5 | MeJA responsive element |
| PpdEXPA16 | G-box | CACGTG | 578 | 6 | Photoresponsive element |
| PpdEXPA16 | G-box | CACGTC | 739 | 6 | Photoresponsive element |
| PpdEXPA16 | G-Box | CACGTG | 578 | 6 | Photoresponsive element |
| PpdEXPA16 | GT1-motif | GGTTAA | 39 | 6 | Photoresponsive element |
| PpdEXPA16 | GT1-motif | GGTTAA | 395 | 6 | Photoresponsive element |
| PpdEXPA16 | MYB | CAACCA | 248 | 6 | Photoresponsive element |
| PpdEXPA16 | MYB | CAACCA | 603 | 6 | Photoresponsive element |
| PpdEXPA16 | MYB | CAACCA | 1491 | 6 | Photoresponsive element |
| PpdEXPA16 | MYB | CAACCA | 1734 | 6 | Photoresponsive element |
| PpdEXPA16 | STRE | AGGGG | 786 | 5 | Stress Response Element |
| PpdEXPA16 | STRE | AGGGG | 1873 | 5 | Stress Response Element |
| PpdEXPA16 | STRE | AGGGG | 1879 | 5 | Stress Response Element |
| PpdEXPA16 | STRE | AGGGG | 1885 | 5 | Stress Response Element |
| PpdEXPA16 | TC-rich repeats | GTTTTCTTAC | 1815 | 9 | Defense and stress responsive element |
| PpdEXPA16 | TGACG-motif | TGACG | 738 | 5 | MeJA responsive element |
| PpdEXPA16 | WRE3 | CCACCT | 1521 | 6 | Wound response，Drought responsive element |
| PpdEXPA16 | WUN-motif | AAATTACTA | 1742 | 9 | Wound responsive element |
| PpdEXPA16 | WUN-motif | AAATTACT | 1743 | 8 | Wound responsive element |
| PpdEXPA17 | ABRE | AACCCGG | 1473 | 7 | Abscisic acid responsive element |
| PpdEXPA17 | ABRE | ACGTG | 1506 | 5 | Abscisic acid responsive element |
| PpdEXPA17 | ABRE | ACGTG | 1824 | 5 | Abscisic acid responsive element |
| PpdEXPA17 | ABRE | CACGTG | 1887 | 6 | Abscisic acid responsive element |
| PpdEXPA17 | ABRE | ACGTG | 1888 | 5 | Abscisic acid responsive element |
| PpdEXPA17 | as-1 | TGACG | 825 | 5 | Drought responsive element |
| PpdEXPA17 | as-1 | TGACG | 1508 | 5 | Drought responsive element |
| PpdEXPA17 | as-1 | TGACG | 1934 | 5 | Drought responsive element |
| PpdEXPA17 | Box 4 | ATTAAT | 67 | 6 | Photoresponsive element |
| PpdEXPA17 | Box 4 | ATTAAT | 324 | 6 | Photoresponsive element |
| PpdEXPA17 | Box 4 | ATTAAT | 741 | 6 | Photoresponsive element |
| PpdEXPA17 | Box 4 | ATTAAT | 750 | 6 | Photoresponsive element |
| PpdEXPA17 | CGTCA-motif | CGTCA | 825 | 5 | MeJA responsive element |
| PpdEXPA17 | CGTCA-motif | CGTCA | 1508 | 5 | MeJA responsive element |
| PpdEXPA17 | CGTCA-motif | CGTCA | 1934 | 5 | MeJA responsive element |
| PpdEXPA17 | G-Box | CACGTT | 1823 | 6 | Photoresponsive element |
| PpdEXPA17 | G-Box | CACGTG | 1887 | 6 | Photoresponsive element |
| PpdEXPA17 | G-box | CACGTC | 1506 | 6 | Photoresponsive element |
| PpdEXPA17 | G-box | ACACGTGT | 1886 | 8 | Photoresponsive element |
| PpdEXPA17 | G-box | CACGTG | 1887 | 6 | Photoresponsive element |
| PpdEXPA17 | GT1-motif | GGTTAA | 394 | 6 | Photoresponsive element |
| PpdEXPA17 | GT1-motif | GGTTAA | 429 | 6 | Photoresponsive element |
| PpdEXPA17 | LTR | CCGAAA | 1113 | 6 | Low temperature responsive element |
| PpdEXPA17 | MYB | TAACCA | 395 | 6 | Photoresponsive element |
| PpdEXPA17 | MYB | TAACCA | 430 | 6 | Photoresponsive element |
| PpdEXPA17 | MYB | TAACCA | 1406 | 6 | Photoresponsive element |
| PpdEXPA17 | MYB | CAACAG | 1511 | 6 | Photoresponsive element |
| PpdEXPA17 | MYB | CAACCA | 1542 | 6 | Photoresponsive element |
| PpdEXPA17 | TC-rich repeats | ATTCTCTAAC | 424 | 9 | Defense and stress responsive element |
| PpdEXPA17 | TCT-motif | TCTTAC | 560 | 6 | Photoresponsive element |
| PpdEXPA17 | TCT-motif | TCTTAC | 1703 | 6 | Photoresponsive element |
| PpdEXPA17 | TGACG-motif | TGACG | 825 | 5 | MeJA responsive element |
| PpdEXPA17 | TGACG-motif | TGACG | 1508 | 5 | MeJA responsive element |
| PpdEXPA17 | TGACG-motif | TGACG | 1934 | 5 | MeJA responsive element |
| PpdEXPA17 | W box | TTGACC | 1646 | 6 | Adversity response component，developmental regulation |
| PpdEXPA17 | WUN-motif | AAATTACTA | 248 | 9 | Wound responsive element |
| PpdEXPA17 | WUN-motif | AAATTACT | 249 | 8 | Wound responsive element |
| PpdEXPA18 | ARE | AAACCA | 591 | 6 | Abscisic acid responsive element |
| PpdEXPA18 | Box 4 | ATTAAT | 1371 | 6 | Photoresponsive element |
| PpdEXPA18 | Box 4 | ATTAAT | 1718 | 6 | Photoresponsive element |
| PpdEXPA18 | ERE | ATTTTAAA | 1985 | 8 | Ethylene responsive element |
| PpdEXPA18 | GATA-motif | GATAGGA | 1170 | 7 | Photoresponsive element |
| PpdEXPA18 | GT1-motif | GGTTAA | 1242 | 6 | Photoresponsive element |
| PpdEXPA18 | MBS | CAACTG | 1901 | 6 | Drought responsive element |
| PpdEXPA18 | MYB | CAACCA | 539 | 6 | Photoresponsive element |
| PpdEXPA18 | MYB | TAACCA | 810 | 6 | Photoresponsive element |
| PpdEXPA18 | MYB | TAACCA | 968 | 6 | Photoresponsive element |
| PpdEXPA18 | MYB | TAACCA | 1255 | 6 | Photoresponsive element |
| PpdEXPA18 | MYB | CAACCA | 1956 | 6 | Photoresponsive element |
| PpdEXPA18 | Myb | TAACTG | 348 | 6 | Photoresponsive element |
| PpdEXPA18 | Myb | TAACTG | 481 | 6 | Photoresponsive element |
| PpdEXPA18 | Myb | CAACTG | 1901 | 6 | Photoresponsive element |
| PpdEXPA18 | STRE | AGGGG | 2 | 5 | Stress Response Element |
| PpdEXPA18 | STRE | AGGGG | 468 | 5 | Stress Response Element |
| PpdEXPA18 | STRE | AGGGG | 715 | 5 | Stress Response Element |
| PpdEXPA18 | STRE | AGGGG | 1697 | 5 | Stress Response Element |
| PpdEXPA18 | STRE | AGGGG | 1731 | 5 | Stress Response Element |
| PpdEXPA18 | TC-rich repeats | ATTCTCTAAC | 1210 | 9 | Defense and stress responsive element |
| PpdEXPA19 | AE-box | AGAAACAA | 680 | 8 | Photoresponsive element |
| PpdEXPA19 | ARE | AAACCA | 1484 | 6 | Abscisic acid responsive element |
| PpdEXPA19 | Box 4 | ATTAAT | 1011 | 6 | Photoresponsive element |
| PpdEXPA19 | Box 4 | ATTAAT | 1596 | 6 | Photoresponsive element |
| PpdEXPA19 | ERE | ATTTTAAA | 252 | 8 | Ethylene responsive element |
| PpdEXPA19 | ERE | ATTTCATA | 738 | 8 | Ethylene responsive element |
| PpdEXPA19 | ERE | ATTTTAAA | 1371 | 8 | Ethylene responsive element |
| PpdEXPA19 | ERE | ATTTCATA | 1419 | 8 | Ethylene responsive element |
| PpdEXPA19 | G-box | TAACACGTAG | 709 | 9 | Photoresponsive element |
| PpdEXPA19 | GT1-motif | GGTTAAT | 348 | 7 | Photoresponsive element |
| PpdEXPA19 | GT1-motif | GGTTAA | 1932 | 6 | Photoresponsive element |
| PpdEXPA19 | I-box | GTATAAGGCC | 98 | 9 | Photoresponsive element |
| PpdEXPA19 | MBS | CAACTG | 467 | 6 | Drought responsive element |
| PpdEXPA19 | MYB | CAACAG | 72 | 6 | Photoresponsive element |
| PpdEXPA19 | MYB | CAACCA | 85 | 6 | Photoresponsive element |
| PpdEXPA19 | MYB | CAACAG | 1586 | 6 | Photoresponsive element |
| PpdEXPA19 | MYB | TAACCA | 1883 | 6 | Photoresponsive element |
| PpdEXPA19 | MYB | TAACCA | 1933 | 6 | Photoresponsive element |
| PpdEXPA19 | Myb | CAACTG | 467 | 6 | Photoresponsive element |
| PpdEXPA19 | Myb | TAACTG | 720 | 6 | Photoresponsive element |
| PpdEXPA19 | Myb | TAACTG | 930 | 6 | Photoresponsive element |
| PpdEXPA19 | TCA | TCATCTTCAT | 1865 | 9 | Salicylic acid responsive element |
| PpdEXPA19 | TCA-element | CCATCTTTTT | 1669 | 9 | Salicylic acid responsive element |
| PpdEXPA19 | TCT-motif | TCTTAC | 879 | 6 | Photoresponsive element |
| PpdEXPA19 | TCT-motif | TCTTAC | 1303 | 6 | Photoresponsive element |
| PpdEXPA19 | W box | TTGACC | 88 | 6 | Adversity response component，developmental regulation |
| PpdEXPA19 | WUN-motif | TAATTACTC | 1537 | 9 | Wound responsive element |
| PpdEXPA20 | ABRE | ACGTG | 1051 | 5 | Abscisic acid responsive element |
| PpdEXPA20 | ABRE | ACGTG | 1227 | 5 | Abscisic acid responsive element |
| PpdEXPA20 | ABRE | ACGTG | 1240 | 5 | Abscisic acid responsive element |
| PpdEXPA20 | ABRE | ACGTG | 1253 | 5 | Abscisic acid responsive element |
| PpdEXPA20 | ABRE | ACGTG | 1761 | 5 | Abscisic acid responsive element |
| PpdEXPA20 | ABRE3a | TACGTG | 1240 | 6 | Abscisic acid responsive element |
| PpdEXPA20 | ABRE4 | CACGTA | 1240 | 6 | Abscisic acid responsive element |
| PpdEXPA20 | AE-box | AGAAACAA | 429 | 8 | Photoresponsive element |
| PpdEXPA20 | ARE | AAACCA | 149 | 6 | Abscisic acid responsive element |
| PpdEXPA20 | ARE | AAACCA | 1103 | 6 | Abscisic acid responsive element |
| PpdEXPA20 | as-1 | TGACG | 1049 | 5 | Drought responsive element |
| PpdEXPA20 | as-1 | TGACG | 1157 | 5 | Drought responsive element |
| PpdEXPA20 | Box 4 | ATTAAT | 500 | 6 | Photoresponsive element |
| PpdEXPA20 | CGTCA-motif | CGTCA | 1049 | 5 | MeJA responsive element |
| PpdEXPA20 | CGTCA-motif | CGTCA | 1157 | 5 | MeJA responsive element |
| PpdEXPA20 | ERE | ATTTTAAA | 440 | 8 | Ethylene responsive element |
| PpdEXPA20 | ERE | ATTTCATA | 589 | 8 | Ethylene responsive element |
| PpdEXPA20 | ERE | ATTTTAAA | 733 | 8 | Ethylene responsive element |
| PpdEXPA20 | G-box | CACGTC | 1050 | 6 | Photoresponsive element |
| PpdEXPA20 | G-box | TACGTG | 1240 | 6 | Photoresponsive element |
| PpdEXPA20 | G-Box | CACGTT | 1227 | 6 | Photoresponsive element |
| PpdEXPA20 | G-Box | CACGTT | 1252 | 6 | Photoresponsive element |
| PpdEXPA20 | G-Box | CACGTT | 1761 | 6 | Photoresponsive element |
| PpdEXPA20 | GT1-motif | GGTTAA | 1933 | 6 | Photoresponsive element |
| PpdEXPA20 | I-box | gGATAAGGTG | 508 | 9 | Photoresponsive element |
| PpdEXPA20 | LTR | CCGAAA | 322 | 6 | Low temperature responsive element |
| PpdEXPA20 | MYB | CAACAG | 622 | 6 | Photoresponsive element |
| PpdEXPA20 | MYB | TAACCA | 936 | 6 | Photoresponsive element |
| PpdEXPA20 | MYB | TAACCA | 1884 | 6 | Photoresponsive element |
| PpdEXPA20 | MYB | TAACCA | 1934 | 6 | Photoresponsive element |
| PpdEXPA20 | STRE | AGGGG | 1071 | 5 | Stress Response Element |
| PpdEXPA20 | TCA | TCATCTTCAT | 1866 | 9 | Salicylic acid responsive element |
| PpdEXPA20 | TC-rich repeats | ATTCTCTAAC | 460 | 9 | Defense and stress responsive element |
| PpdEXPA20 | TCT-motif | TCTTAC | 1257 | 6 | Photoresponsive element |
| PpdEXPA20 | TCT-motif | TCTTAC | 1369 | 6 | Photoresponsive element |
| PpdEXPA20 | TCT-motif | TCTTAC | 1844 | 6 | Photoresponsive element |
| PpdEXPA20 | TGACG-motif | TGACG | 1049 | 5 | MeJA responsive element |
| PpdEXPA20 | TGACG-motif | TGACG | 1157 | 5 | MeJA responsive element |
| PpdEXPA20 | W box | TTGACC | 73 | 6 | Adversity response component，developmental regulation |
| PpdEXPA20 | W box | TTGACC | 144 | 6 | Adversity response component，developmental regulation |
| PpdEXPA20 | W box | TTGACC | 301 | 6 | Adversity response component，developmental regulation |
| PpdEXPA20 | W box | TTGACC | 318 | 6 | Adversity response component，developmental regulation |
| PpdEXPA20 | W box | TTGACC | 453 | 6 | Adversity response component，developmental regulation |
| PpdEXPA20 | W box | TTGACC | 1208 | 6 | Adversity response component，developmental regulation |
| PpdEXPA20 | W box | TTGACC | 1235 | 6 | Adversity response component，developmental regulation |
| PpdEXPA20 | W box | TTGACC | 1273 | 6 | Adversity response component，developmental regulation |
| PpdEXPA20 | W box | TTGACC | 1322 | 6 | Adversity response component，developmental regulation |
| PpdEXPA20 | W box | TTGACC | 1803 | 6 | Adversity response component，developmental regulation |
| PpdEXPA21 | ABRE | CACGTG | 888 | 6 | Abscisic acid responsive element |
| PpdEXPA21 | ABRE | ACGTG | 889 | 5 | Abscisic acid responsive element |
| PpdEXPA21 | ABRE | GCCGCGTGGC | 1608 | 9 | Abscisic acid responsive element |
| PpdEXPA21 | ARE | AAACCA | 133 | 6 | Abscisic acid responsive element |
| PpdEXPA21 | ARE | AAACCA | 380 | 6 | Abscisic acid responsive element |
| PpdEXPA21 | ARE | AAACCA | 1270 | 6 | Abscisic acid responsive element |
| PpdEXPA21 | ARE | AAACCA | 1606 | 6 | Abscisic acid responsive element |
| PpdEXPA21 | Box 4 | ATTAAT | 557 | 6 | Photoresponsive element |
| PpdEXPA21 | Box 4 | ATTAAT | 1486 | 6 | Photoresponsive element |
| PpdEXPA21 | ERE | ATTTTAAA | 511 | 8 | Ethylene responsive element |
| PpdEXPA21 | ERE | ATTTTAAA | 696 | 8 | Ethylene responsive element |
| PpdEXPA21 | G-box | CACGAC | 755 | 6 | Photoresponsive element |
| PpdEXPA21 | G-box | CACGTG | 888 | 6 | Photoresponsive element |
| PpdEXPA21 | G-Box | CACGTG | 888 | 6 | Photoresponsive element |
| PpdEXPA21 | GT1-motif | GGTTAAT | 184 | 7 | Photoresponsive element |
| PpdEXPA21 | GT1-motif | GGTTAA | 185 | 6 | Photoresponsive element |
| PpdEXPA21 | GT1-motif | GGTTAA | 1037 | 6 | Photoresponsive element |
| PpdEXPA21 | GT1-motif | GGTTAA | 1203 | 6 | Photoresponsive element |
| PpdEXPA21 | GT1-motif | GGTTAA | 1257 | 6 | Photoresponsive element |
| PpdEXPA21 | LTR | CCGAAA | 245 | 6 | Low temperature responsive element |
| PpdEXPA21 | MYB | CAACAG | 342 | 6 | Photoresponsive element |
| PpdEXPA21 | MYB | TAACCA | 544 | 6 | Photoresponsive element |
| PpdEXPA21 | MYB | TAACCA | 1036 | 6 | Photoresponsive element |
| PpdEXPA21 | MYB | TAACCA | 1256 | 6 | Photoresponsive element |
| PpdEXPA21 | Myb | TAACTG | 1858 | 6 | Photoresponsive element |
| PpdEXPA21 | MYB recognition site | CCGTTG | 1408 | 6 | Photoresponsive element |
| PpdEXPA21 | TCA | TCATCTTCAT | 231 | 9 | Salicylic acid responsive element |
| PpdEXPA21 | TC-rich repeats | GTTTTCTTAC | 1600 | 9 | Defense and stress responsive element |
| PpdEXPA22 | ABRE | ACGTG | 978 | 5 | Abscisic acid responsive element |
| PpdEXPA22 | ABRE3a | TACGTG | 978 | 6 | Abscisic acid responsive element |
| PpdEXPA22 | ABRE4 | CACGTA | 978 | 6 | Abscisic acid responsive element |
| PpdEXPA22 | AE-box | AGAAACAA | 38 | 8 | Photoresponsive element |
| PpdEXPA22 | ARE | AAACCA | 192 | 6 | Abscisic acid responsive element |
| PpdEXPA22 | Box 4 | ATTAAT | 24 | 6 | Photoresponsive element |
| PpdEXPA22 | Box 4 | ATTAAT | 895 | 6 | Photoresponsive element |
| PpdEXPA22 | Box 4 | ATTAAT | 1601 | 6 | Photoresponsive element |
| PpdEXPA22 | ERE | ATTTTAAA | 312 | 8 | Ethylene responsive element |
| PpdEXPA22 | ERE | ATTTCATA | 457 | 8 | Ethylene responsive element |
| PpdEXPA22 | G-box | TACGTG | 978 | 6 | Photoresponsive element |
| PpdEXPA22 | MBS | CAACTG | 494 | 6 | Drought responsive element |
| PpdEXPA22 | MYB | CAACAG | 1080 | 6 | Photoresponsive element |
| PpdEXPA22 | MYB | TAACCA | 1271 | 6 | Photoresponsive element |
| PpdEXPA22 | Myb | CAACTG | 494 | 6 | Photoresponsive element |
| PpdEXPA22 | STRE | AGGGG | 89 | 5 | Stress Response Element |
| PpdEXPA22 | STRE | AGGGG | 758 | 5 | Stress Response Element |
| PpdEXPA22 | STRE | AGGGG | 1403 | 5 | Stress Response Element |
| PpdEXPA22 | TCA | TCATCTTCAT | 1325 | 9 | Salicylic acid responsive element |
| PpdEXPA22 | TCA-element | CCATCTTTTT | 152 | 9 | Salicylic acid responsive element |
| PpdEXPA22 | TC-rich repeats | ATTCTCTAAC | 1350 | 9 | Defense and stress responsive element |
| PpdEXPA22 | TGA-element | AACGAC | 119 | 6 | Auxin responsive element |
| PpdEXPA22 | TGA-element | AACGAC | 1075 | 6 | Auxin responsive element |
| PpdEXPA22 | TGA-element | AACGAC | 1238 | 6 | Auxin responsive element |
| PpdEXPA23 | ARE | AAACCA | 1541 | 6 | Abscisic acid responsive element |
| PpdEXPA23 | ARE | AAACCA | 1820 | 6 | Abscisic acid responsive element |
| PpdEXPA23 | Box 4 | ATTAAT | 134 | 6 | Photoresponsive element |
| PpdEXPA23 | GATA-motif | GATAGGA | 1476 | 7 | Photoresponsive element |
| PpdEXPA23 | LTR | CCGAAA | 915 | 6 | Low temperature responsive element |
| PpdEXPA23 | LTR | CCGAAA | 1286 | 6 | Low temperature responsive element |
| PpdEXPA23 | MBS | CAACTG | 1010 | 6 | Drought responsive element |
| PpdEXPA23 | Myb | TAACTG | 812 | 6 | Photoresponsive element |
| PpdEXPA23 | Myb | CAACTG | 1010 | 6 | Photoresponsive element |
| PpdEXPA23 | Myb | TAACTG | 1785 | 6 | Photoresponsive element |
| PpdEXPA23 | MYB | CAACCA | 313 | 6 | Photoresponsive element |
| PpdEXPA23 | MYB | CAACCA | 725 | 6 | Photoresponsive element |
| PpdEXPA23 | MYB | TAACCA | 836 | 6 | Photoresponsive element |
| PpdEXPA23 | MYB | CAACCA | 1305 | 6 | Photoresponsive element |
| PpdEXPA23 | MYB | CAACCA | 1815 | 6 | Photoresponsive element |
| PpdEXPA23 | MYB recognition site | CCGTTG | 1292 | 6 | Photoresponsive element |
| PpdEXPA23 | STRE | AGGGG | 1359 | 5 | Stress Response Element |
| PpdEXPA23 | TCA | TCATCTTCAT | 1415 | 9 | Salicylic acid responsive element |
| PpdEXPA23 | TC-rich repeats | ATTCTCTAAC | 830 | 9 | Defense and stress responsive element |
| PpdEXPA23 | TC-rich repeats | GTTTTCTTAC | 1326 | 9 | Defense and stress responsive element |
| PpdEXPA23 | TCT-motif | TCTTAC | 50 | 6 | Photoresponsive element |
| PpdEXPA23 | TCT-motif | TCTTAC | 326 | 6 | Photoresponsive element |
| PpdEXPA23 | TCT-motif | TCTTAC | 676 | 6 | Photoresponsive element |
| PpdEXPA23 | TGA-element | AACGAC | 1297 | 6 | Auxin responsive element |
| PpdEXPA23 | WUN-motif | AAATTTCTT | 1183 | 9 | Wound responsive element |
| PpdEXPA24 | ABRE | ACGTG | 758 | 5 | Abscisic acid responsive element |
| PpdEXPA24 | ABRE | TACGTGTC | 982 | 8 | Abscisic acid responsive element |
| PpdEXPA24 | ABRE | ACGTG | 983 | 5 | Abscisic acid responsive element |
| PpdEXPA24 | ABRE | GCAACGTGTC | 1544 | 9 | Abscisic acid responsive element |
| PpdEXPA24 | ABRE | ACGTG | 1547 | 5 | Abscisic acid responsive element |
| PpdEXPA24 | ABRE3a | TACGTG | 982 | 6 | Abscisic acid responsive element |
| PpdEXPA24 | ABRE4 | CACGTA | 982 | 6 | Abscisic acid responsive element |
| PpdEXPA24 | AE-box | AGAAACAA | 853 | 8 | Photoresponsive element |
| PpdEXPA24 | AE-box | AGAAACAA | 1348 | 8 | Photoresponsive element |
| PpdEXPA24 | AE-box | AGAAACAA | 1760 | 8 | Photoresponsive element |
| PpdEXPA24 | Box 4 | ATTAAT | 84 | 6 | Photoresponsive element |
| PpdEXPA24 | Box 4 | ATTAAT | 143 | 6 | Photoresponsive element |
| PpdEXPA24 | Box 4 | ATTAAT | 165 | 6 | Photoresponsive element |
| PpdEXPA24 | Box 4 | ATTAAT | 676 | 6 | Photoresponsive element |
| PpdEXPA24 | Box 4 | ATTAAT | 1127 | 6 | Photoresponsive element |
| PpdEXPA24 | ERE | ATTTTAAA | 783 | 8 | Ethylene responsive element |
| PpdEXPA24 | ERE | ATTTTAAA | 1099 | 8 | Ethylene responsive element |
| PpdEXPA24 | ERE | ATTTTAAA | 1185 | 8 | Ethylene responsive element |
| PpdEXPA24 | G-box | TACGTG | 982 | 6 | Photoresponsive element |
| PpdEXPA24 | G-Box | CACGTT | 758 | 6 | Photoresponsive element |
| PpdEXPA24 | G-Box | CACGTT | 1546 | 6 | Photoresponsive element |
| PpdEXPA24 | GT1-motif | GTGTGTGAA | 729 | 9 | Photoresponsive element |
| PpdEXPA24 | GT1-motif | GGTTAA | 959 | 6 | Photoresponsive element |
| PpdEXPA24 | LTR | CCGAAA | 28 | 6 | Low temperature responsive element |
| PpdEXPA24 | LTR | CCGAAA | 313 | 6 | Low temperature responsive element |
| PpdEXPA24 | LTR | CCGAAA | 321 | 6 | Low temperature responsive element |
| PpdEXPA24 | LTR | CCGAAA | 469 | 6 | Low temperature responsive element |
| PpdEXPA24 | LTR | CCGAAA | 476 | 6 | Low temperature responsive element |
| PpdEXPA24 | LTR | CCGAAA | 512 | 6 | Low temperature responsive element |
| PpdEXPA24 | LTR | CCGAAA | 1001 | 6 | Low temperature responsive element |
| PpdEXPA24 | MYB recognition site | CCGTTG | 521 | 6 | Photoresponsive element |
| PpdEXPA24 | W box | TTGACC | 1202 | 6 | Adversity response component，developmental regulation |
| PpdEXPA24 | WUN-motif | AAATTACTA | 1902 | 9 | Wound responsive element |
| PpdEXPB1 | ABRE | ACGTG | 690 | 5 | Abscisic acid responsive element |
| PpdEXPB1 | ABRE3a | TACGTG | 690 | 6 | Abscisic acid responsive element |
| PpdEXPB1 | ABRE4 | CACGTA | 690 | 6 | Abscisic acid responsive element |
| PpdEXPB1 | AE-box | AGAAACAA | 1822 | 8 | Photoresponsive element |
| PpdEXPB1 | ARE | AAACCA | 919 | 6 | Abscisic acid responsive element |
| PpdEXPB1 | as-1 | TGACG | 1169 | 5 | Drought responsive element |
| PpdEXPB1 | as-1 | TGACG | 1321 | 5 | Drought responsive element |
| PpdEXPB1 | as-1 | TGACG | 1748 | 5 | Drought responsive element |
| PpdEXPB1 | as-1 | TGACG | 1756 | 5 | Drought responsive element |
| PpdEXPB1 | Box 4 | ATTAAT | 66 | 6 | Photoresponsive element |
| PpdEXPB1 | Box 4 | ATTAAT | 899 | 6 | Photoresponsive element |
| PpdEXPB1 | Box 4 | ATTAAT | 1846 | 6 | Photoresponsive element |
| PpdEXPB1 | CGTCA-motif | CGTCA | 1169 | 5 | MeJA responsive element |
| PpdEXPB1 | CGTCA-motif | CGTCA | 1321 | 5 | MeJA responsive element |
| PpdEXPB1 | CGTCA-motif | CGTCA | 1748 | 5 | MeJA responsive element |
| PpdEXPB1 | CGTCA-motif | CGTCA | 1756 | 5 | MeJA responsive element |
| PpdEXPB1 | ERE | ATTTTAAA | 909 | 8 | Ethylene responsive element |
| PpdEXPB1 | G-box | TACGTG | 690 | 6 | Photoresponsive element |
| PpdEXPB1 | G-box | TAACACGTAG | 734 | 9 | Photoresponsive element |
| PpdEXPB1 | MYB | CAACAG | 1869 | 6 | Photoresponsive element |
| PpdEXPB1 | MYB recognition site | CCGTTG | 1092 | 6 | Photoresponsive element |
| PpdEXPB1 | STRE | AGGGG | 621 | 5 | Stress Response Element |
| PpdEXPB1 | TCA-element | TCAGAAGAGG | 1162 | 9 | Salicylic acid responsive element |
| PpdEXPB1 | TGACG-motif | TGACG | 1169 | 5 | MeJA responsive element |
| PpdEXPB1 | TGACG-motif | TGACG | 1321 | 5 | MeJA responsive element |
| PpdEXPB1 | TGACG-motif | TGACG | 1748 | 5 | MeJA responsive element |
| PpdEXPB1 | TGACG-motif | TGACG | 1756 | 5 | MeJA responsive element |
| PpdEXPB1 | TGA-element | AACGAC | 1009 | 6 | Auxin responsive element |
| PpdEXPB1 | TGA-element | AACGAC | 1073 | 6 | Auxin responsive element |
| PpdEXPB1 | WUN-motif | CCATTTCAA | 430 | 9 | Wound responsive element |
| PpdEXPB2 | ABRE | CGCACGTGTC | 1488 | 9 | Abscisic acid responsive element |
| PpdEXPB2 | AE-box | AGAAACAA | 143 | 8 | Photoresponsive element |
| PpdEXPB2 | AE-box | AGAAACTT | 1057 | 8 | Photoresponsive element |
| PpdEXPB2 | ARE | AAACCA | 255 | 6 | Abscisic acid responsive element |
| PpdEXPB2 | ARE | AAACCA | 1357 | 6 | Abscisic acid responsive element |
| PpdEXPB2 | ARE | AAACCA | 1362 | 6 | Abscisic acid responsive element |
| PpdEXPB2 | ARE | AAACCA | 1367 | 6 | Abscisic acid responsive element |
| PpdEXPB2 | ARE | AAACCA | 1372 | 6 | Abscisic acid responsive element |
| PpdEXPB2 | ARE | AAACCA | 1377 | 6 | Abscisic acid responsive element |
| PpdEXPB2 | as-1 | TGACG | 894 | 5 | Drought responsive element |
| PpdEXPB2 | as-1 | TGACG | 1593 | 5 | Drought responsive element |
| PpdEXPB2 | as-1 | TGACG | 1791 | 5 | Drought responsive element |
| PpdEXPB2 | Box 4 | ATTAAT | 833 | 6 | Photoresponsive element |
| PpdEXPB2 | CGTCA-motif | CGTCA | 894 | 5 | MeJA responsive element |
| PpdEXPB2 | CGTCA-motif | CGTCA | 1593 | 5 | MeJA responsive element |
| PpdEXPB2 | CGTCA-motif | CGTCA | 1791 | 5 | MeJA responsive element |
| PpdEXPB2 | ERE | ATTTCATA | 411 | 8 | Ethylene responsive element |
| PpdEXPB2 | ERE | ATTTCATA | 632 | 8 | Ethylene responsive element |
| PpdEXPB2 | G-Box | TCCACATGGCA | 1660 | 10 | Photoresponsive element |
| PpdEXPB2 | G-box | CACGAC | 1495 | 6 | Photoresponsive element |
| PpdEXPB2 | LTR | CCGAAA | 1610 | 6 | Low temperature responsive element |
| PpdEXPB2 | MBS | CAACTG | 694 | 6 | Drought responsive element |
| PpdEXPB2 | MYB | TAACCA | 444 | 6 | Photoresponsive element |
| PpdEXPB2 | MYB | CAACCA | 546 | 6 | Photoresponsive element |
| PpdEXPB2 | MYB | TAACCA | 672 | 6 | Photoresponsive element |
| PpdEXPB2 | MYB | TAACCA | 1043 | 6 | Photoresponsive element |
| PpdEXPB2 | MYB | TAACCA | 1168 | 6 | Photoresponsive element |
| PpdEXPB2 | MYB | CAACAG | 1908 | 6 | Photoresponsive element |
| PpdEXPB2 | Myb | TAACTG | 25 | 6 | Photoresponsive element |
| PpdEXPB2 | Myb | CAACTG | 694 | 6 | Photoresponsive element |
| PpdEXPB2 | MYB recognition site | CCGTTG | 682 | 6 | Photoresponsive element |
| PpdEXPB2 | TC-rich repeats | GTTTTCTTAC | 336 | 9 | Defense and stress responsive element |
| PpdEXPB2 | TCT-motif | TCTTAC | 1084 | 6 | Photoresponsive element |
| PpdEXPB2 | TGACG-motif | TGACG | 894 | 5 | MeJA responsive element |
| PpdEXPB2 | TGACG-motif | TGACG | 1593 | 5 | MeJA responsive element |
| PpdEXPB2 | TGACG-motif | TGACG | 1791 | 5 | MeJA responsive element |
| PpdEXPB2 | TGA-element | AACGAC | 663 | 6 | Auxin responsive element |
| PpdEXPB2 | WRE3 | CCACCT | 750 | 6 | Wound response，Drought responsive element |
| PpdEXPB3 | ABRE | AACCCGG | 573 | 7 | Abscisic acid responsive element |
| PpdEXPB3 | AE-box | AGAAACTT | 843 | 8 | Photoresponsive element |
| PpdEXPB3 | ARE | AAACCA | 1942 | 6 | Abscisic acid responsive element |
| PpdEXPB3 | Box 4 | ATTAAT | 1712 | 6 | Photoresponsive element |
| PpdEXPB3 | Box 4 | ATTAAT | 1952 | 6 | Photoresponsive element |
| PpdEXPB3 | GATA-motif | AAGGATAAGG | 1144 | 9 | Photoresponsive element |
| PpdEXPB3 | GATA-motif | AAGGATAAGG | 1319 | 10 | Photoresponsive element |
| PpdEXPB3 | G-box | CACGAC | 434 | 6 | Photoresponsive element |
| PpdEXPB3 | GT1-motif | GGTTAA | 1213 | 6 | Photoresponsive element |
| PpdEXPB3 | I-box | AGATAAGG | 1146 | 8 | Photoresponsive element |
| PpdEXPB3 | I-box | ccttatcct | 1319 | 9 | Photoresponsive element |
| PpdEXPB3 | MYB | CAACCA | 196 | 6 | Photoresponsive element |
| PpdEXPB3 | MYB | CAACCA | 389 | 6 | Photoresponsive element |
| PpdEXPB3 | MYB | CAACAG | 1621 | 6 | Photoresponsive element |
| PpdEXPB3 | MYB | CAACAG | 1911 | 6 | Photoresponsive element |
| PpdEXPB3 | STRE | AGGGG | 754 | 5 | Stress Response Element |
| PpdEXPB3 | STRE | AGGGG | 1266 | 5 | Stress Response Element |
| PpdEXPB3 | STRE | AGGGG | 1293 | 5 | Stress Response Element |
| PpdEXPB3 | STRE | AGGGG | 1850 | 5 | Stress Response Element |
| PpdEXPB3 | W box | TTGACC | 1464 | 6 | Adversity response component，developmental regulation |
| PpdEXPB3 | WRE3 | CCACCT | 126 | 6 | Wound response，Drought responsive element |
| PpdEXPB3 | WRE3 | CCACCT | 660 | 6 | Wound response，Drought responsive element |
| PpdEXPB4 | AE-box | AGAAACAA | 588 | 8 | Photoresponsive element |
| PpdEXPB4 | ARE | AAACCA | 45 | 6 | Abscisic acid responsive element |
| PpdEXPB4 | Box 4 | ATTAAT | 572 | 6 | Photoresponsive element |
| PpdEXPB4 | Box 4 | ATTAAT | 673 | 6 | Photoresponsive element |
| PpdEXPB4 | Box 4 | ATTAAT | 901 | 6 | Photoresponsive element |
| PpdEXPB4 | Box 4 | ATTAAT | 1039 | 6 | Photoresponsive element |
| PpdEXPB4 | Box 4 | ATTAAT | 1067 | 6 | Photoresponsive element |
| PpdEXPB4 | Box 4 | ATTAAT | 1083 | 6 | Photoresponsive element |
| PpdEXPB4 | Box 4 | ATTAAT | 1087 | 6 | Photoresponsive element |
| PpdEXPB4 | Box 4 | ATTAAT | 1099 | 6 | Photoresponsive element |
| PpdEXPB4 | Box 4 | ATTAAT | 1109 | 6 | Photoresponsive element |
| PpdEXPB4 | Box 4 | ATTAAT | 1143 | 6 | Photoresponsive element |
| PpdEXPB4 | Box 4 | ATTAAT | 1209 | 6 | Photoresponsive element |
| PpdEXPB4 | Box 4 | ATTAAT | 1535 | 6 | Photoresponsive element |
| PpdEXPB4 | Box 4 | ATTAAT | 1674 | 6 | Photoresponsive element |
| PpdEXPB4 | Box 4 | ATTAAT | 1689 | 6 | Photoresponsive element |
| PpdEXPB4 | Box 4 | ATTAAT | 1772 | 6 | Photoresponsive element |
| PpdEXPB4 | ERE | ATTTTAAA | 34 | 8 | Ethylene responsive element |
| PpdEXPB4 | G-box | TAACACGTAG | 178 | 9 | Photoresponsive element |
| PpdEXPB4 | GT1-motif | GGTTAA | 1438 | 6 | Photoresponsive element |
| PpdEXPB4 | LTR | CCGAAA | 147 | 6 | Low temperature responsive element |
| PpdEXPB4 | LTR | CCGAAA | 730 | 6 | Low temperature responsive element |
| PpdEXPB4 | Myb | TAACTG | 979 | 6 | Photoresponsive element |
| PpdEXPB4 | Myb | TAACTG | 997 | 6 | Photoresponsive element |
| PpdEXPB4 | Myb | TAACTG | 1626 | 6 | Photoresponsive element |
| PpdEXPB4 | MYB | TAACCA | 1437 | 6 | Photoresponsive element |
| PpdEXPB4 | MYB | CAACCA | 1807 | 6 | Photoresponsive element |
| PpdEXPB4 | TCA-element | CCATCTTTTT | 1430 | 9 | Salicylic acid responsive element |
| PpdEXPB4 | W box | TTGACC | 833 | 6 | Adversity response component，developmental regulation |
| PpdEXPB4 | WRE3 | CCACCT | 278 | 6 | Wound response，Drought responsive element |
| PpdEXPB4 | WUN-motif | AAATTACT | 474 | 8 | Wound responsive element |
| PpdEXPB5 | ABRE | ACGTG | 1017 | 5 | Abscisic acid responsive element |
| PpdEXPB5 | ABRE | ACGTG | 1812 | 5 | Abscisic acid responsive element |
| PpdEXPB5 | ARE | AAACCA | 110 | 6 | Abscisic acid responsive element |
| PpdEXPB5 | ARE | AAACCA | 1279 | 6 | Abscisic acid responsive element |
| PpdEXPB5 | Box 4 | ATTAAT | 434 | 6 | Photoresponsive element |
| PpdEXPB5 | Box 4 | ATTAAT | 609 | 6 | Photoresponsive element |
| PpdEXPB5 | Box 4 | ATTAAT | 629 | 6 | Photoresponsive element |
| PpdEXPB5 | Box 4 | ATTAAT | 839 | 6 | Photoresponsive element |
| PpdEXPB5 | Box 4 | ATTAAT | 882 | 6 | Photoresponsive element |
| PpdEXPB5 | Box 4 | ATTAAT | 1054 | 6 | Photoresponsive element |
| PpdEXPB5 | Box 4 | ATTAAT | 1628 | 6 | Photoresponsive element |
| PpdEXPB5 | Box 4 | ATTAAT | 1685 | 6 | Photoresponsive element |
| PpdEXPB5 | ERE | ATTTTAAA | 1042 | 8 | Ethylene responsive element |
| PpdEXPB5 | GATA-motif | GATAGGA | 752 | 7 | Photoresponsive element |
| PpdEXPB5 | G-box | TAAACGTG | 1809 | 8 | Photoresponsive element |
| PpdEXPB5 | G-Box | CACGTT | 1017 | 6 | Photoresponsive element |
| PpdEXPB5 | G-Box | CACGTT | 1811 | 6 | Photoresponsive element |
| PpdEXPB5 | GT1-motif | GGTTAAT | 1623 | 7 | Photoresponsive element |
| PpdEXPB5 | I-box | TGATAATGT | 445 | 9 | Photoresponsive element |
| PpdEXPB5 | I-box | AGATAAGG | 1744 | 8 | Photoresponsive element |
| PpdEXPB5 | MYB | TAACCA | 1622 | 6 | Photoresponsive element |
| PpdEXPB5 | MYB | CAACCA | 1766 | 6 | Photoresponsive element |
| PpdEXPB5 | MYB | CAACCA | 1991 | 6 | Photoresponsive element |
| PpdEXPB5 | TC-rich repeats | GTTTTCTTAC | 766 | 9 | Defense and stress responsive element |
| PpdEXPB5 | TCT-motif | TCTTAC | 770 | 6 | Photoresponsive element |
| PpdEXPB5 | WRE3 | CCACCT | 271 | 6 | Wound response，Drought responsive element |
| PpdEXPB6 | ABRE | ACGTG | 936 | 5 | Abscisic acid responsive element |
| PpdEXPB6 | ABRE | ACGTG | 1382 | 5 | Abscisic acid responsive element |
| PpdEXPB6 | ABRE | ACGTG | 1807 | 5 | Abscisic acid responsive element |
| PpdEXPB6 | ABRE3a | TACGTG | 1381 | 6 | Abscisic acid responsive element |
| PpdEXPB6 | ABRE4 | CACGTA | 1381 | 6 | Abscisic acid responsive element |
| PpdEXPB6 | ARE | AAACCA | 1201 | 6 | Abscisic acid responsive element |
| PpdEXPB6 | Box 4 | ATTAAT | 308 | 6 | Photoresponsive element |
| PpdEXPB6 | Box 4 | ATTAAT | 328 | 6 | Photoresponsive element |
| PpdEXPB6 | Box 4 | ATTAAT | 364 | 6 | Photoresponsive element |
| PpdEXPB6 | Box 4 | ATTAAT | 974 | 6 | Photoresponsive element |
| PpdEXPB6 | Box 4 | ATTAAT | 1628 | 6 | Photoresponsive element |
| PpdEXPB6 | Box 4 | ATTAAT | 1685 | 6 | Photoresponsive element |
| PpdEXPB6 | ERE | ATTTTAAA | 961 | 8 | Ethylene responsive element |
| PpdEXPB6 | ERE | ATTTTAAA | 1553 | 8 | Ethylene responsive element |
| PpdEXPB6 | GATA-motif | AAGGATAAGG | 126 | 9 | Photoresponsive element |
| PpdEXPB6 | GATA-motif | GATAGGA | 451 | 7 | Photoresponsive element |
| PpdEXPB6 | GATA-motif | AAGATAAGATT | 558 | 10 | Photoresponsive element |
| PpdEXPB6 | G-Box | CACGTT | 936 | 6 | Photoresponsive element |
| PpdEXPB6 | G-box | TACGTG | 1381 | 6 | Photoresponsive element |
| PpdEXPB6 | G-box | CACGTC | 1806 | 6 | Photoresponsive element |
| PpdEXPB6 | GT1-motif | GGTTAAT | 1623 | 7 | Photoresponsive element |
| PpdEXPB6 | I-box | AGATAAGG | 1744 | 8 | Photoresponsive element |
| PpdEXPB6 | MYB | CAACCA | 1601 | 6 | Photoresponsive element |
| PpdEXPB6 | MYB | TAACCA | 1622 | 6 | Photoresponsive element |
| PpdEXPB6 | MYB | CAACCA | 1766 | 6 | Photoresponsive element |
| PpdEXPB6 | MYB | CAACCA | 1991 | 6 | Photoresponsive element |
| PpdEXPB6 | TCA-element | CCATCTTTTT | 138 | 9 | Salicylic acid responsive element |
| PpdEXPB6 | TCA-element | CCATCTTTTT | 171 | 9 | Salicylic acid responsive element |
| PpdEXPB6 | TC-rich repeats | GTTTTCTTAC | 465 | 9 | Defense and stress responsive element |
| PpdEXPB6 | TCT-motif | TCTTAC | 469 | 6 | Photoresponsive element |
| PpdEXPB6 | WRE3 | CCACCT | 142 | 6 | Wound response，Drought responsive element |
| PpdEXPB6 | WUN-motif | TAATTACTC | 952 | 9 | Wound responsive element |
| PpdEXPB6 | WUN-motif | AAATTACT | 1206 | 8 | Wound responsive element |
| PpdEXPB7 | ABRE | ACGTG | 487 | 5 | Abscisic acid responsive element |
| PpdEXPB7 | ABRE | ACGTG | 1836 | 5 | Abscisic acid responsive element |
| PpdEXPB7 | ABRE3a | TACGTG | 487 | 6 | Abscisic acid responsive element |
| PpdEXPB7 | ABRE4 | CACGTA | 487 | 6 | Abscisic acid responsive element |
| PpdEXPB7 | ARE | AAACCA | 878 | 6 | Abscisic acid responsive element |
| PpdEXPB7 | ARE | AAACCA | 895 | 6 | Abscisic acid responsive element |
| PpdEXPB7 | ARE | AAACCA | 1139 | 6 | Abscisic acid responsive element |
| PpdEXPB7 | ARE | AAACCA | 1447 | 6 | Abscisic acid responsive element |
| PpdEXPB7 | Box 4 | ATTAAT | 29 | 6 | Photoresponsive element |
| PpdEXPB7 | Box 4 | ATTAAT | 664 | 6 | Photoresponsive element |
| PpdEXPB7 | Box 4 | ATTAAT | 841 | 6 | Photoresponsive element |
| PpdEXPB7 | ERE | ATTTTAAA | 1007 | 8 | Ethylene responsive element |
| PpdEXPB7 | GATA-motif | GATAGGG | 824 | 7 | Photoresponsive element |
| PpdEXPB7 | GATA-motif | GATAGGA | 1510 | 7 | Photoresponsive element |
| PpdEXPB7 | G-box | TACGTG | 487 | 6 | Photoresponsive element |
| PpdEXPB7 | G-box | CACGTC | 1836 | 6 | Photoresponsive element |
| PpdEXPB7 | LTR | CCGAAA | 436 | 6 | Low temperature responsive element |
| PpdEXPB7 | MYB | CAACAG | 388 | 6 | Photoresponsive element |
| PpdEXPB7 | MYB | TAACCA | 419 | 6 | Photoresponsive element |
| PpdEXPB7 | MYB | CAACAG | 1940 | 6 | Photoresponsive element |
| PpdEXPB7 | STRE | AGGGG | 1455 | 5 | Stress Response Element |
| PpdEXPB7 | TCA-element | CCATCTTTTT | 644 | 9 | Salicylic acid responsive element |
| PpdEXPB7 | TCA-element | CCATCTTTTT | 790 | 9 | Salicylic acid responsive element |
| PpdEXPB7 | WUN-motif | AAATTACT | 467 | 8 | Wound responsive element |
| PpdEXPB8 | ABRE | AACCCGG | 35 | 7 | Abscisic acid responsive element |
| PpdEXPB8 | ABRE | GACACGTGGC | 98 | 9 | Abscisic acid responsive element |
| PpdEXPB8 | ABRE | AACCCGG | 240 | 7 | Abscisic acid responsive element |
| PpdEXPB8 | ABRE | CGTACGTGCA | 1010 | 9 | Abscisic acid responsive element |
| PpdEXPB8 | ABRE | ACGTG | 1013 | 5 | Abscisic acid responsive element |
| PpdEXPB8 | as-1 | TGACG | 285 | 5 | Drought responsive element |
| PpdEXPB8 | as-1 | TGACG | 442 | 5 | Drought responsive element |
| PpdEXPB8 | Box 4 | ATTAAT | 687 | 6 | Photoresponsive element |
| PpdEXPB8 | Box 4 | ATTAAT | 884 | 6 | Photoresponsive element |
| PpdEXPB8 | Box 4 | ATTAAT | 1453 | 6 | Photoresponsive element |
| PpdEXPB8 | Box 4 | ATTAAT | 1685 | 6 | Photoresponsive element |
| PpdEXPB8 | CGTCA-motif | CGTCA | 285 | 5 | MeJA responsive element |
| PpdEXPB8 | CGTCA-motif | CGTCA | 442 | 5 | MeJA responsive element |
| PpdEXPB8 | ERE | ATTTTAAA | 9 | 8 | Ethylene responsive element |
| PpdEXPB8 | ERE | ATTTTAAA | 1085 | 8 | Ethylene responsive element |
| PpdEXPB8 | GATA-motif | AAGGATAAGG | 760 | 9 | Photoresponsive element |
| PpdEXPB8 | G-box | CACGTC | 1012 | 6 | Photoresponsive element |
| PpdEXPB8 | GT1-motif | GGTTAA | 1267 | 6 | Photoresponsive element |
| PpdEXPB8 | I-box | gGATAAGGTG | 762 | 10 | Photoresponsive element |
| PpdEXPB8 | I-box | GTATAAGGCC | 1118 | 9 | Photoresponsive element |
| PpdEXPB8 | LTR | CCGAAA | 620 | 6 | Low temperature responsive element |
| PpdEXPB8 | Myb | TAACTG | 587 | 6 | Photoresponsive element |
| PpdEXPB8 | Myb | TAACTG | 1904 | 6 | Photoresponsive element |
| PpdEXPB8 | MYB | CAACCA | 1018 | 6 | Photoresponsive element |
| PpdEXPB8 | MYB recognition site | CCGTTG | 869 | 6 | Photoresponsive element |
| PpdEXPB8 | MYB recognition site | CCGTTG | 1299 | 6 | Photoresponsive element |
| PpdEXPB8 | STRE | AGGGG | 560 | 5 | Stress Response Element |
| PpdEXPB8 | TC-rich repeats | GTTTTCTTAC | 1176 | 9 | Defense and stress responsive element |
| PpdEXPB8 | TCT-motif | TCTTAC | 570 | 6 | Photoresponsive element |
| PpdEXPB8 | TGACG-motif | TGACG | 285 | 5 | MeJA responsive element |
| PpdEXPB8 | TGACG-motif | TGACG | 442 | 5 | MeJA responsive element |
| PpdEXPB8 | TGA-element | AACGAC | 1073 | 6 | Auxin responsive element |
| PpdEXPB8 | TGA-element | AACGAC | 1203 | 6 | Auxin responsive element |
| PpdEXPB8 | W box | TTGACC | 30 | 6 | Adversity response component，developmental regulation |
| PpdEXPB8 | W box | TTGACC | 167 | 6 | Adversity response component，developmental regulation |
| PpdEXPB8 | W box | TTGACC | 236 | 6 | Adversity response component，developmental regulation |
| PpdEXPB8 | WRE3 | CCACCT | 91 | 6 | Wound response，Drought responsive element |
| PpdEXPB8 | WRE3 | CCACCT | 299 | 6 | Wound response，Drought responsive element |
| PpdEXPB8 | WRE3 | CCACCT | 313 | 6 | Wound response，Drought responsive element |
| PpdEXPB8 | WRE3 | CCACCT | 349 | 6 | Wound response，Drought responsive element |
| PpdEXPB8 | WRE3 | CCACCT | 741 | 6 | Wound response，Drought responsive element |
| PpdEXPB8 | WUN-motif | TTATTACAT | 40 | 9 | Wound responsive element |
| PpdEXPB8 | WUN-motif | TTATTACAT | 245 | 9 | Wound responsive element |
| PpdEXPB8 | WUN-motif | TAATTACTC | 1634 | 9 | Wound responsive element |
| PpdEXPB9 | ABRE | ACGTG | 685 | 5 | Abscisic acid responsive element |
| PpdEXPB9 | ABRE | TACGGTC | 1219 | 7 | Abscisic acid responsive element |
| PpdEXPB9 | ABRE | CACGTG | 1563 | 6 | Abscisic acid responsive element |
| PpdEXPB9 | ABRE | ACGTG | 1564 | 5 | Abscisic acid responsive element |
| PpdEXPB9 | ARE | AAACCA | 1764 | 6 | Abscisic acid responsive element |
| PpdEXPB9 | Box 4 | ATTAAT | 36 | 6 | Photoresponsive element |
| PpdEXPB9 | Box 4 | ATTAAT | 510 | 6 | Photoresponsive element |
| PpdEXPB9 | Box 4 | ATTAAT | 835 | 6 | Photoresponsive element |
| PpdEXPB9 | Box 4 | ATTAAT | 1294 | 6 | Photoresponsive element |
| PpdEXPB9 | G-Box | CACGTT | 685 | 6 | Photoresponsive element |
| PpdEXPB9 | G-Box | CACGTG | 1563 | 6 | Photoresponsive element |
| PpdEXPB9 | G-box | CACGTG | 1563 | 6 | Photoresponsive element |
| PpdEXPB9 | GT1-motif | GGTTAA | 1109 | 6 | Photoresponsive element |
| PpdEXPB9 | MBS | CAACTG | 182 | 6 | Drought responsive element |
| PpdEXPB9 | Myb | CAACTG | 182 | 6 | Photoresponsive element |
| PpdEXPB9 | Myb | TAACTG | 526 | 6 | Photoresponsive element |
| PpdEXPB9 | MYB recognition site | CCGTTG | 1537 | 6 | Photoresponsive element |
| PpdEXPB9 | STRE | AGGGG | 426 | 5 | Stress Response Element |
| PpdEXPB9 | STRE | AGGGG | 1317 | 5 | Stress Response Element |
| PpdEXPB9 | TCA | TCATCTTCAT | 1140 | 9 | Salicylic acid responsive element |
| PpdEXPB9 | TGA-element | AACGAC | 1820 | 6 | Auxin responsive element |
| PpdEXPB9 | TGA-element | AACGAC | 1827 | 6 | Auxin responsive element |
| PpdEXPB9 | W box | TTGACC | 474 | 6 | Adversity response component，developmental regulation |
| PpdEXPB9 | WRE3 | CCACCT | 1902 | 6 | Wound response，Drought responsive element |
| PpdEXPB9 | WUN-motif | AAATTACT | 590 | 8 | Wound responsive element |
| PpdEXPB9 | WUN-motif | AAATTACT | 1459 | 8 | Wound responsive element |
| PpdEXPLA1 | ABRE | ACGTG | 627 | 5 | Abscisic acid responsive element |
| PpdEXPLA1 | ABRE | ACGTG | 899 | 5 | Abscisic acid responsive element |
| PpdEXPLA1 | ABRE | ACGTG | 909 | 5 | Abscisic acid responsive element |
| PpdEXPLA1 | AE-box | AGAAACTT | 50 | 8 | Photoresponsive element |
| PpdEXPLA1 | ARE | AAACCA | 957 | 6 | Abscisic acid responsive element |
| PpdEXPLA1 | Box 4 | ATTAAT | 261 | 6 | Photoresponsive element |
| PpdEXPLA1 | Box 4 | ATTAAT | 454 | 6 | Photoresponsive element |
| PpdEXPLA1 | Box 4 | ATTAAT | 556 | 6 | Photoresponsive element |
| PpdEXPLA1 | Box 4 | ATTAAT | 654 | 6 | Photoresponsive element |
| PpdEXPLA1 | Box 4 | ATTAAT | 777 | 6 | Photoresponsive element |
| PpdEXPLA1 | Box 4 | ATTAAT | 1651 | 6 | Photoresponsive element |
| PpdEXPLA1 | Box 4 | ATTAAT | 1698 | 6 | Photoresponsive element |
| PpdEXPLA1 | Box 4 | ATTAAT | 1995 | 6 | Photoresponsive element |
| PpdEXPLA1 | ERE | ATTTCATA | 126 | 8 | Ethylene responsive element |
| PpdEXPLA1 | ERE | ATTTCATA | 1001 | 8 | Ethylene responsive element |
| PpdEXPLA1 | GATA-motif | GATAGGG | 1068 | 7 | Photoresponsive element |
| PpdEXPLA1 | G-Box | CACGTT | 627 | 6 | Photoresponsive element |
| PpdEXPLA1 | G-Box | CACGTT | 909 | 6 | Photoresponsive element |
| PpdEXPLA1 | G-box | CACGTC | 899 | 6 | Photoresponsive element |
| PpdEXPLA1 | GT1-motif | GGTTAA | 534 | 6 | Photoresponsive element |
| PpdEXPLA1 | LTR | CCGAAA | 1306 | 6 | Low temperature responsive element |
| PpdEXPLA1 | LTR | CCGAAA | 1457 | 6 | Low temperature responsive element |
| PpdEXPLA1 | LTR | CCGAAA | 1500 | 6 | Low temperature responsive element |
| PpdEXPLA1 | Myb | TAACTG | 1028 | 6 | Photoresponsive element |
| PpdEXPLA1 | MYB | TAACCA | 382 | 6 | Photoresponsive element |
| PpdEXPLA1 | MYB | TAACCA | 535 | 6 | Photoresponsive element |
| PpdEXPLA1 | MYB | CAACAG | 698 | 6 | Photoresponsive element |
| PpdEXPLA1 | MYB | TAACCA | 1147 | 6 | Photoresponsive element |
| PpdEXPLA1 | STRE | AGGGG | 1071 | 5 | Stress Response Element |
| PpdEXPLA1 | STRE | AGGGG | 1864 | 5 | Stress Response Element |
| PpdEXPLA1 | TCA-element | CCATCTTTTT | 1043 | 9 | Salicylic acid responsive element |
| PpdEXPLA1 | TCT-motif | TCTTAC | 1744 | 6 | Photoresponsive element |
| PpdEXPLA1 | W box | TTGACC | 1331 | 6 | Adversity response component，developmental regulation |
| PpdEXPLA1 | W box | TTGACC | 1352 | 6 | Adversity response component，developmental regulation |
| PpdEXPLA2 | ARE | AAACCA | 132 | 6 | Abscisic acid responsive element |
| PpdEXPLA2 | ARE | AAACCA | 727 | 6 | Abscisic acid responsive element |
| PpdEXPLA2 | as-1 | TGACG | 1000 | 5 | Drought responsive element |
| PpdEXPLA2 | as-1 | TGACG | 1171 | 5 | Drought responsive element |
| PpdEXPLA2 | as-1 | TGACG | 1205 | 5 | Drought responsive element |
| PpdEXPLA2 | as-1 | TGACG | 1329 | 5 | Drought responsive element |
| PpdEXPLA2 | as-1 | TGACG | 1348 | 5 | Drought responsive element |
| PpdEXPLA2 | as-1 | TGACG | 1366 | 5 | Drought responsive element |
| PpdEXPLA2 | as-1 | TGACG | 1456 | 5 | Drought responsive element |
| PpdEXPLA2 | as-1 | TGACG | 1488 | 5 | Drought responsive element |
| PpdEXPLA2 | Box 4 | ATTAAT | 353 | 6 | Photoresponsive element |
| PpdEXPLA2 | Box 4 | ATTAAT | 1570 | 6 | Photoresponsive element |
| PpdEXPLA2 | CGTCA-motif | CGTCA | 1000 | 5 | MeJA responsive element |
| PpdEXPLA2 | CGTCA-motif | CGTCA | 1171 | 5 | MeJA responsive element |
| PpdEXPLA2 | CGTCA-motif | CGTCA | 1205 | 5 | MeJA responsive element |
| PpdEXPLA2 | CGTCA-motif | CGTCA | 1329 | 5 | MeJA responsive element |
| PpdEXPLA2 | CGTCA-motif | CGTCA | 1348 | 5 | MeJA responsive element |
| PpdEXPLA2 | CGTCA-motif | CGTCA | 1366 | 5 | MeJA responsive element |
| PpdEXPLA2 | CGTCA-motif | CGTCA | 1456 | 5 | MeJA responsive element |
| PpdEXPLA2 | CGTCA-motif | CGTCA | 1488 | 5 | MeJA responsive element |
| PpdEXPLA2 | GATA-motif | GATAGGG | 1675 | 7 | Photoresponsive element |
| PpdEXPLA2 | LTR | CCGAAA | 760 | 6 | Low temperature responsive element |
| PpdEXPLA2 | MYB | TAACCA | 84 | 6 | Photoresponsive element |
| PpdEXPLA2 | MYB | TAACCA | 456 | 6 | Photoresponsive element |
| PpdEXPLA2 | MYB | CAACCA | 594 | 6 | Photoresponsive element |
| PpdEXPLA2 | MYB | TAACCA | 1780 | 6 | Photoresponsive element |
| PpdEXPLA2 | MYB recognition site | CCGTTG | 151 | 6 | Photoresponsive element |
| PpdEXPLA2 | STRE | AGGGG | 267 | 5 | Stress Response Element |
| PpdEXPLA2 | STRE | AGGGG | 277 | 5 | Stress Response Element |
| PpdEXPLA2 | STRE | AGGGG | 311 | 5 | Stress Response Element |
| PpdEXPLA2 | STRE | AGGGG | 1678 | 5 | Stress Response Element |
| PpdEXPLA2 | STRE | AGGGG | 1864 | 5 | Stress Response Element |
| PpdEXPLA2 | TGACG-motif | TGACG | 1000 | 5 | MeJA responsive element |
| PpdEXPLA2 | TGACG-motif | TGACG | 1171 | 5 | MeJA responsive element |
| PpdEXPLA2 | TGACG-motif | TGACG | 1205 | 5 | MeJA responsive element |
| PpdEXPLA2 | TGACG-motif | TGACG | 1329 | 5 | MeJA responsive element |
| PpdEXPLA2 | TGACG-motif | TGACG | 1348 | 5 | MeJA responsive element |
| PpdEXPLA2 | TGACG-motif | TGACG | 1366 | 5 | MeJA responsive element |
| PpdEXPLA2 | TGACG-motif | TGACG | 1456 | 5 | MeJA responsive element |
| PpdEXPLA2 | TGACG-motif | TGACG | 1488 | 5 | MeJA responsive element |
| PpdEXPLA2 | TGA-element | AACGAC | 1398 | 6 | Auxin responsive element |
| PpdEXPLA2 | W box | TTGACC | 1227 | 6 | Adversity response component，developmental regulation |
| PpdEXPLA2 | WRE3 | CCACCT | 591 | 6 | Wound response，Drought responsive element |
| PpdEXPLB1 | ABRE | ACGTG | 353 | 5 | Abscisic acid responsive element |
| PpdEXPLB1 | ABRE | ACGTG | 616 | 5 | Abscisic acid responsive element |
| PpdEXPLB1 | ABRE | ACGTG | 622 | 5 | Abscisic acid responsive element |
| PpdEXPLB1 | ABRE3a | TACGTG | 352 | 6 | Abscisic acid responsive element |
| PpdEXPLB1 | ABRE4 | CACGTA | 352 | 6 | Abscisic acid responsive element |
| PpdEXPLB1 | ARE | AAACCA | 1398 | 6 | Abscisic acid responsive element |
| PpdEXPLB1 | ARE | AAACCA | 1414 | 6 | Abscisic acid responsive element |
| PpdEXPLB1 | as-1 | TGACG | 596 | 5 | Drought responsive element |
| PpdEXPLB1 | Box 4 | ATTAAT | 1459 | 6 | Photoresponsive element |
| PpdEXPLB1 | CGTCA-motif | CGTCA | 596 | 5 | MeJA responsive element |
| PpdEXPLB1 | GATA-motif | GATAGGA | 148 | 7 | Photoresponsive element |
| PpdEXPLB1 | G-Box | CACGTT | 621 | 6 | Photoresponsive element |
| PpdEXPLB1 | G-box | TACGTG | 352 | 6 | Photoresponsive element |
| PpdEXPLB1 | G-box | CACGTC | 615 | 6 | Photoresponsive element |
| PpdEXPLB1 | G-box | GCCACGTGGA | 1788 | 9 | Photoresponsive element |
| PpdEXPLB1 | GT1-motif | GGTTAA | 506 | 6 | Photoresponsive element |
| PpdEXPLB1 | Myb | TAACTG | 236 | 6 | Photoresponsive element |
| PpdEXPLB1 | Myb | TAACTG | 1422 | 6 | Photoresponsive element |
| PpdEXPLB1 | MYB | TAACCA | 270 | 6 | Photoresponsive element |
| PpdEXPLB1 | MYB | CAACAG | 911 | 6 | Photoresponsive element |
| PpdEXPLB1 | MYB | CAACCA | 1124 | 6 | Photoresponsive element |
| PpdEXPLB1 | MYB | CAACAG | 1325 | 6 | Photoresponsive element |
| PpdEXPLB1 | MYB recognition site | CCGTTG | 478 | 6 | Photoresponsive element |
| PpdEXPLB1 | MYB recognition site | CCGTTG | 701 | 6 | Photoresponsive element |
| PpdEXPLB1 | MYB recognition site | CCGTTG | 725 | 6 | Photoresponsive element |
| PpdEXPLB1 | STRE | AGGGG | 190 | 5 | Stress Response Element |
| PpdEXPLB1 | STRE | AGGGG | 806 | 5 | Stress Response Element |
| PpdEXPLB1 | TCA-element | CCATCTTTTT | 967 | 9 | Salicylic acid responsive element |
| PpdEXPLB1 | TCT-motif | TCTTAC | 471 | 6 | Photoresponsive element |
| PpdEXPLB1 | TCT-motif | TCTTAC | 1914 | 6 | Photoresponsive element |
| PpdEXPLB1 | TGACG-motif | TGACG | 596 | 5 | MeJA responsive element |
| PpdEXPLB1 | W box | TTGACC | 1345 | 6 | Adversity response component，developmental regulation |
| PpdEXPLB1 | WRE3 | CCACCT | 1822 | 6 | Wound response，Drought responsive element |
| PpdEXPLB2 | AE-box | AGAAACAA | 311 | 8 | Photoresponsive element |
| PpdEXPLB2 | ARE | AAACCA | 1287 | 6 | Abscisic acid responsive element |
| PpdEXPLB2 | as-1 | TGACG | 193 | 5 | Drought responsive element |
| PpdEXPLB2 | Box 4 | ATTAAT | 1370 | 6 | Photoresponsive element |
| PpdEXPLB2 | CGTCA-motif | CGTCA | 193 | 5 | MeJA responsive element |
| PpdEXPLB2 | ERE | ATTTCATA | 1172 | 8 | Ethylene responsive element |
| PpdEXPLB2 | ERE | ATTTTAAA | 1445 | 8 | Ethylene responsive element |
| PpdEXPLB2 | GT1-motif | GGTTAAT | 568 | 7 | Photoresponsive element |
| PpdEXPLB2 | Myb | TAACTG | 112 | 6 | Photoresponsive element |
| PpdEXPLB2 | Myb | TAACTG | 131 | 6 | Photoresponsive element |
| PpdEXPLB2 | Myb | TAACTG | 1257 | 6 | Photoresponsive element |
| PpdEXPLB2 | MYB | TAACCA | 567 | 6 | Photoresponsive element |
| PpdEXPLB2 | MYB | TAACCA | 1907 | 6 | Photoresponsive element |
| PpdEXPLB2 | STRE | AGGGG | 40 | 5 | Stress Response Element |
| PpdEXPLB2 | STRE | AGGGG | 385 | 5 | Stress Response Element |
| PpdEXPLB2 | STRE | AGGGG | 1045 | 5 | Stress Response Element |
| PpdEXPLB2 | STRE | AGGGG | 1095 | 5 | Stress Response Element |
| PpdEXPLB2 | TCA | TCATCTTCAT | 88 | 9 | Salicylic acid responsive element |
| PpdEXPLB2 | TCA-element | TCAGAAGAGG | 1047 | 9 | Salicylic acid responsive element |
| PpdEXPLB2 | TCT-motif | TCTTAC | 1334 | 6 | Photoresponsive element |
| PpdEXPLB2 | TGACG-motif | TGACG | 193 | 5 | MeJA responsive element |
| PpdEXPLB2 | WRE3 | CCACCT | 1892 | 6 | Wound response，Drought responsive element |
| PpdEXPLB2 | WUN-motif | AAATTACTA | 1700 | 9 | Wound responsive element |
| PpdEXPLB2 | WUN-motif | AAATTACT | 1701 | 8 | Wound responsive element |
| PpdEXPLB3 | AE-box | AGAAACTT | 1638 | 8 | Photoresponsive element |
| PpdEXPLB3 | AE-box | AGAAACAA | 1745 | 8 | Photoresponsive element |
| PpdEXPLB3 | AE-box | AGAAACAA | 1817 | 8 | Photoresponsive element |
| PpdEXPLB3 | ARE | AAACCA | 627 | 6 | Abscisic acid responsive element |
| PpdEXPLB3 | ARE | AAACCA | 799 | 6 | Abscisic acid responsive element |
| PpdEXPLB3 | ARE | AAACCA | 1776 | 6 | Abscisic acid responsive element |
| PpdEXPLB3 | as-1 | TGACG | 1138 | 5 | Drought responsive element |
| PpdEXPLB3 | Box 4 | ATTAAT | 1222 | 6 | Photoresponsive element |
| PpdEXPLB3 | CGTCA-motif | CGTCA | 1138 | 5 | MeJA responsive element |
| PpdEXPLB3 | ERE | ATTTCATA | 100 | 8 | Ethylene responsive element |
| PpdEXPLB3 | ERE | ATTTTAAA | 1233 | 8 | Ethylene responsive element |
| PpdEXPLB3 | GT1-motif | GGTTAAT | 1073 | 7 | Photoresponsive element |
| PpdEXPLB3 | GT1-motif | GGTTAA | 1074 | 6 | Photoresponsive element |
| PpdEXPLB3 | GT1-motif | GGTTAA | 1404 | 6 | Photoresponsive element |
| PpdEXPLB3 | I-box | gGATAAGGTG | 599 | 9 | Photoresponsive element |
| PpdEXPLB3 | LTR | CCGAAA | 1158 | 6 | Low temperature responsive element |
| PpdEXPLB3 | LTR | CCGAAA | 1555 | 6 | Low temperature responsive element |
| PpdEXPLB3 | LTR | CCGAAA | 1588 | 6 | Low temperature responsive element |
| PpdEXPLB3 | MYB | CAACAG | 438 | 6 | Photoresponsive element |
| PpdEXPLB3 | MYB | CAACCA | 522 | 6 | Photoresponsive element |
| PpdEXPLB3 | MYB | CAACCA | 589 | 6 | Photoresponsive element |
| PpdEXPLB3 | MYB | TAACCA | 1403 | 6 | Photoresponsive element |
| PpdEXPLB3 | MYB | CAACAG | 1769 | 6 | Photoresponsive element |
| PpdEXPLB3 | MYB | CAACCA | 1926 | 6 | Photoresponsive element |
| PpdEXPLB3 | Myb | TAACTG | 374 | 6 | Photoresponsive element |
| PpdEXPLB3 | Myb | TAACTG | 414 | 6 | Photoresponsive element |
| PpdEXPLB3 | Myb | TAACTG | 1457 | 6 | Photoresponsive element |
| PpdEXPLB3 | STRE | AGGGG | 1317 | 5 | Stress Response Element |
| PpdEXPLB3 | TCA | TCATCTTCAT | 193 | 10 | Salicylic acid responsive element |
| PpdEXPLB3 | TCA-element | TCAGAAGAGG | 1958 | 9 | Salicylic acid responsive element |
| PpdEXPLB3 | TCT-motif | TCTTAC | 129 | 6 | Photoresponsive element |
| PpdEXPLB3 | TGACG-motif | TGACG | 1138 | 5 | MeJA responsive element |
| PpdEXPLB3 | W box | TTGACC | 897 | 6 | Adversity response component，developmental regulation |
| PpdEXPLB3 | W box | TTGACC | 952 | 6 | Adversity response component，developmental regulation |
| PpdEXPLB3 | W box | TTGACC | 1566 | 6 | Adversity response component，developmental regulation |
| PpdEXPLB3 | WRE3 | CCACCT | 1728 | 6 | Wound response，Drought responsive element |
| PpdEXPLB3 | WUN-motif | AAATTACT | 27 | 8 | Wound responsive element |
| PpdEXPLB4 | ABRE | ACGTG | 1783 | 5 | Abscisic acid responsive element |
| PpdEXPLB4 | ARE | AAACCA | 307 | 6 | Abscisic acid responsive element |
| PpdEXPLB4 | ARE | AAACCA | 672 | 6 | Abscisic acid responsive element |
| PpdEXPLB4 | ARE | AAACCA | 966 | 6 | Abscisic acid responsive element |
| PpdEXPLB4 | ARE | AAACCA | 1696 | 6 | Abscisic acid responsive element |
| PpdEXPLB4 | ARE | AAACCA | 1701 | 6 | Abscisic acid responsive element |
| PpdEXPLB4 | as-1 | TGACG | 1451 | 5 | Drought responsive element |
| PpdEXPLB4 | CGTCA-motif | CGTCA | 1451 | 5 | MeJA responsive element |
| PpdEXPLB4 | G-Box | CACGTT | 1782 | 6 | Photoresponsive element |
| PpdEXPLB4 | LTR | CCGAAA | 1692 | 6 | Low temperature responsive element |
| PpdEXPLB4 | MYB | CAACCA | 1705 | 6 | Photoresponsive element |
| PpdEXPLB4 | STRE | AGGGG | 58 | 5 | Stress Response Element |
| PpdEXPLB4 | STRE | AGGGG | 794 | 5 | Stress Response Element |
| PpdEXPLB4 | STRE | AGGGG | 1218 | 5 | Stress Response Element |
| PpdEXPLB4 | STRE | AGGGG | 1346 | 5 | Stress Response Element |
| PpdEXPLB4 | TCA | TCATCTTCAT | 1989 | 9 | Salicylic acid responsive element |
| PpdEXPLB4 | TCA-element | CCATCTTTTT | 344 | 9 | Salicylic acid responsive element |
| PpdEXPLB4 | TCT-motif | TCTTAC | 79 | 6 | Photoresponsive element |
| PpdEXPLB4 | TCT-motif | TCTTAC | 887 | 6 | Photoresponsive element |
| PpdEXPLB4 | TGACG-motif | TGACG | 1451 | 5 | MeJA responsive element |
| PpdEXPLB4 | W box | TTGACC | 1068 | 6 | Adversity response component，developmental regulation |
| PpdEXPLB4 | WUN-motif | TTATTACAT | 893 | 9 | Wound responsive element |
| PpdEXPLB4 | WUN-motif | CAATTACAT | 1071 | 9 | Wound responsive element |
